# Oral vs Extended-Release Injectable Naltrexone for Hospitalized Patients With Alcohol Use Disorder: A Randomized Clinical Trial

**DOI:** 10.1001/jamainternmed.2025.0522

**Published:** 2025-04-21

**Authors:** Kara M. Magane, Kimberly A. Dukes, Sarah Fielman, Tibor P. Palfai, Daniel Regan, Debbie M. Cheng, Henri Lee, Kevin L. Kraemer, Matthew J. Bullard, Clara A. Chen, Jeffrey H. Samet

**Affiliations:** 1 Department of Community Health Sciences, Boston University School of Public Health, Boston, Massachusetts; 2 Department of Biostatistics, Boston University School of Public Health, Boston, Massachusetts; 3 Department of Psychological and Brain Sciences, Boston University, Boston, Massachusetts; 4 South Shore Hospital, South Weymouth, Massachusetts; 5 Center for Health Data Science, Boston University School of Public Health, Boston, Massachusetts; 6 Section of General Internal Medicine, Department of Medicine, Boston University Chobanian and Avedisian School of Medicine, Boston Massachusetts; 7 Section of General Internal Medicine, Department of Medicine, Boston Medical Center, Boston, Massachusetts; 8 Division of General Internal Medicine, Department of Medicine, University of Pittsburgh School of Medicine, Pittsburgh, Pennsylvania; 9 Grayken Center for Addiction, Boston Medical Center, Boston, Massachusetts

## Abstract

**Importance:**

Alcohol use disorder (AUD) is common in hospital patients. AUD medications are not typically initiated in that setting. The comparative effectiveness between initiation of oral naltrexone and extended-release injectable naltrexone in the hospital is not known.

**Objective:**

To compare the effectiveness of initiating oral naltrexone vs extended-release injectable naltrexone on reduction in alcohol use and health care utilization among medical inpatients with AUD.

**Design, Setting, and Participants:**

The Alcohol Disorder Hospital Treatment (ADOPT) study is a randomized clinical trial conducted at an urban teaching hospital in the US, with enrollment between June 2016 and March 2020. Inpatients were screened for eligibility, and those with AUD and recent heavy drinking (defined as 5 or more drinks for men and 4 or more drinks for women) were enrolled. Outcomes were assessed at 3-month follow-up; assessors were not blinded to treatment assignment. Data were analyzed from May 2021 to September 2023.

**Interventions:**

Participants received either daily oral naltrexone or monthly extended-release injectable naltrexone. All received medical management with a research nurse who specialized in addiction.

**Main Measures and Outcomes:**

The primary outcome was change in percentage of heavy drinking days (HDDs) over the past 30 days from baseline to 3-month follow-up, assessed by validated instrument. The secondary outcome was any acute health care utilization (emergency department or hospitalization) at 3-month follow-up over the past 90 days.

**Results:**

Of 248 participants, 199 (80.2%) were male, and the mean (SD) age was 49.4 (10.4) years. The baseline median (IQR) percentage of HDDs in the past 30 days was 80.0% (43.3-100). At 3-month follow-up, the mean percentage of HDDs in the past 30 days was reduced in both groups (oral naltrexone: baseline, 66.7% HDDs; 3-month follow-up, 27.4% HDDs; difference, −38.4 percentage points; 95% CI, −125.0 to 48.2; extended-release injectable naltrexone: baseline, 70.7% HDDs; 3-month follow-up, 23.8% HDDs; difference, −46.4 percentage points; 95% CI, −123.4 to 30.6; *P* = .14). At follow-up, 59 of 109 in the oral naltrexone arm (54.1%) and 66 of 108 in the extended-release injectable naltrexone arm (61.1%) reported acute health care utilization in the prior 3 months; the odds of this utilization were not significantly different between groups (adjusted odds ratio, 1.34; 95% CI, 0.77-2.33).

**Conclusions and Relevance:**

In this randomized clinical trial, when initiated at hospital discharge, oral and extended-release injectable naltrexone did not differ in effectiveness. Participants had substantial reductions in HDDs in both treatment groups; however, there was not a significant difference in the reduction of percentage of HDDs in the past 30 days or acute health care utilization between groups. Hospitalization represents an opportunity to start AUD pharmacotherapy; choice of oral naltrexone vs extended-release injectable naltrexone should be directed by factors such as patient preference and insurance.

**Trial Registration:**

ClinicalTrials.gov Identifier: NCT02478489

## Key Points

**Question:**

What is the comparative effectiveness of initiating oral vs extended-release injectable naltrexone on heavy drinking (defined as 5 or more drinks for men and 4 or more drinks for women) among hospitalized patients with alcohol use disorder (AUD)?

**Findings:**

In this randomized clinical trial including 248 adults, there was not a statistically significant difference between groups in the 3-month change in percentage of heavy drinking days in the past 30 days (38.4–percentage point decrease in the oral naltrexone group vs 46.4–percentage point decrease in the extended-release injectable naltrexone group).

**Meaning:**

Hospitalization represents an opportunity to start AUD pharmacotherapy; given their similar effectiveness, choice of oral vs extended-release injectable naltrexone should be directed by practical factors, such as patient preference and affordability.

## Teaser

This randomized clinical trial compares the effectiveness of initiating oral naltrexone vs extended-release injectable naltrexone on reduction in alcohol use and health care utilization among medical inpatients with alcohol use disorder.

## Introduction

Nearly 8% of people 12 years or older in the US have alcohol use disorder (AUD).^1^ Unfortunately, 94% of those with AUD do not receive medication or counseling.^2^ A setting in which to expand AUD treatment is the hospital, in which 1 survey found 19% of inpatients have AUD.^3^ Few are discharged with medication^4^ or referrals to treatment,^5^ increasing acute care readmission.^6^

Pharmacotherapies and manualized psychotherapies are the main AUD treatments.^7,8^ Given moderate benefit for AUD medication,^9^ optimizing its delivery could improve outcomes. Naltrexone, the most commonly used AUD medication,^4,10^ is available as an oral daily tablet or monthly injection. Both formulations should be given with medical management (ie, review of alcohol consumption, counseling, assessment of liver enzymes, and medication adverse effects [AEs]). Extended-release injectable naltrexone obviates the need for daily pill taking, potentially an advantage over oral naltrexone. However, patients may prefer daily pill taking to monthly injections due to time and effort required for injection visits, negative attitudes toward injections, and greater control over use of naltrexone.^11^ Extended-release injectable naltrexone is more costly ($1064.00 for 1 injection^12^ vs $38.10 for 30 days of oral naltrexone^13^), although cost-effectiveness could be similar if extended-release injectable naltrexone were more effective with less health care utilization.

Initiating naltrexone at hospital discharge is feasible,^14^ yet to our knowledge, no published randomized clinical trial has compared oral naltrexone and extended-release injectable naltrexone. Therefore, we conducted a randomized clinical trial comparing oral naltrexone and extended-release injectable naltrexone at discharge among hospital patients with AUD. We hypothesized that extended-release injectable naltrexone would result in less alcohol consumption and health care utilization compared with oral naltrexone.

## Methods

### Study Design

The Alcohol Disorder Hospital Treatment (ADOPT) study was an open-label randomized clinical trial comparing the effectiveness of oral naltrexone with extended-release injectable naltrexone initiated in the hospital for patients with AUD with a 3-month follow-up research assessment. A detailed trial protocol is available in Supplement 1. Participants provided written informed consent. The Boston University Medical Campus Institutional Review Board approved the study, including follow-up of incarcerated participants. The study had a Certificate of Confidentiality from National Institutes of Health. Participants were compensated ($75 each for baseline and 3-month follow-up). This trial followed the Consolidated Standards of Reporting Trials (CONSORT) reporting guideline.

### Participants

Participants were recruited from June 29, 2016, to March 4, 2020, from an urban academic hospital in the US. Recruitment methods included clinician referral (12.5%) by hospital primary medical team or Addiction Consult Service and medical record review (87.5%), wherein research assistants reviewed inpatient medical records. Research assistants approached potentially eligible inpatients and completed AUD screening using *Diagnostic and Statistical Manual of Mental Disorders* (Fifth Edition) criteria and Alcohol Use Disorder and Associated Disabilities Interview Schedule.^15^ Patients with AUD were further evaluated by a research nurse (D.R.) regarding additional eligibility criteria.

Inclusion criteria were AUD; 1 or more heavy drinking days (HDDs; defined as 5 or more drinks for men and 4 or more drinks for women) in the 30 days prior to hospitalization; general medical hospital inpatients; age of 18 years or older; English speaking; and willingness to name 2 or more contacts to assist with study follow-up. Exclusion criteria were pregnancy (determined by urine testing); current breastfeeding; self-reported opioid use in the past week for long-acting opioids or in the past 24 hours for short-acting opioids; positive result on urine dipstick testing for any opioid; planned discharge prescription for opioids; need for opioids for anticipated painful event or surgery in the next 3 months; current suicidality or psychosis; cognitive dysfunction precluding informed consent; alanine aminotransferase or aspartate aminotransferase levels more than 5 times the upper limit of normal; acute hepatitis or liver failure; platelet count less than 50 × 10^3^/μL; coagulopathy; body habitus precluding intramuscular injection; naltrexone hypersensitivity; plans to leave the area within 1 year; and enrollment in a study involving a pharmaceutical with potential naltrexone interactions.

### Assessments and Measures

Assessments occurred at baseline (prior to randomization) and 3-month follow-up. Research assistants completed a standardized research interview collecting self-reported sociodemographic characteristics, including sex, age, race (including American Indian or Alaskan Native, Asian, Black or African American, Native Hawaiian or Pacific Islander, White, or more than 1 race), Hispanic ethnicity, highest level of education, marital status, unhoused (1 or more nights on the street or in a shelter in the past 3 months), and prior 3-month incarceration. Race and ethnicity data were collected as required by the National Institutes of Health and because previous research suggested possible interactions between Black race and naltrexone on efficacy outcomes.^16^ Participants reported alcohol and substance use, with related problems and motivations including number of drinks consumed on each day in the past 30 days using the Timeline Followback calendar method^17^ (used to generate alcohol abstinence in the 7 days prior to hospitalization, World Health Organization drinking risk level,^18^ and HDDs); AUD severity (mild, moderate, or severe) assessed via the Alcohol Use Disorder and Associated Disabilities Interview Schedule^19^; the Alcohol, Smoking, and Substance Involvement Screening Test drug use items^20^; the Short Inventory of Problems Revision 2^21^; the Readiness Ruler assessing readiness to change^22^; the Penn Alcohol Craving Scale^23^; the Drinking Motives Questionnaire^24^; and the Reasons For Quitting scale.^25^ Health-related characteristics, including depressive and anxiety symptoms, stress, recent injury, general health and quality of life, pain, tobacco use, health insurance, and diagnoses (used to generate Charlson Comorbidity Index score^26^), were assessed and are detailed in eMethods 1 in Supplement 2.

We modified questions from the Form 90 Alcohol Intake Revised^27^ to collect self-report of health care and addiction treatment utilization in the prior 3 months, including nights spent in the hospital, alcohol-related nights spent in the hospital, emergency department (ED) visits, alcohol-related ED visits, nights spent in an inpatient treatment facility, alcohol-related nights spent in an inpatient treatment facility, outpatient visits, alcohol-related outpatient visits, medications taken for AUD, and participation in mutual help groups (eg, Alcoholics Anonymous).

Liver enzymes (alanine aminotransferase and aspartate aminotransferase) were assessed at study entry and at 3-month follow-up. Blood specimens collected at follow-up were analyzed for alcohol biomarkers (ie, γ-glutamyltransferase [GGT], disialo-carbohydrate–deficient transferrin [dCDT], and phosphatidylethanol) and naltrexone use (6-β-naltrexol).

### Randomization and Interventions

Participants were randomly assigned in a 1:1 ratio to either oral naltrexone or extended-release injectable naltrexone, using random block sizes of 2 and 4. Randomization was stratified by sex and race (dichotomized as Black or other race). Allocation, generated by the trial’s data coordinating center, was concealed from study staff until the moment of assignment. Given the nature of the interventions, blinding was not possible.

Participants randomized to oral naltrexone received a bottle with 30 tablets on the day of discharge and 1 and 2 months later during real-world medical management visits (eMethods 2 in Supplement 2). Participants were instructed to take naltrexone, 25 mg, daily for 3 days, then naltrexone, 50 mg, daily. Based on tolerability and not reaching goals, the dosage was increased to 100 mg per day; dose reductions and symptomatic therapies were considered if AEs occurred. Participants randomized to extended-release injectable naltrexone received 1 intramuscular gluteal injection of extended-release naltrexone, 380 mg, during hospitalization on the day of hospital discharge (if weekend discharge was scheduled, injection was administered on the most proximal weekday prior to discharge). The 3-month follow-up window began on the date that the first dose of study medication was dispensed. Participants received extended-release injectable naltrexone 1 and 2 months later. Both arms received real-world medical management from the study nurse (D.R.; eMethods 2 in Supplement 2) after randomization and 1, 2, and 3 months subsequently as outpatients.

### Outcomes

The primary outcome was change in percentage of HDDs in the past 30 days from baseline to 3-month follow-up. The secondary outcome was any self-reported acute health care utilization (ED visits or hospitalizations) over the prior 3 months^27^ at 3-month follow-up. We performed a comparison between self-reported utilization (ED visits or hospitalizations) and data obtained from participants’ electronic medical records from the hospital system at which the study occurred (eTable 1 in Supplement 2). Other self-reported outcomes assessed at 3-month follow-up referencing the prior 3 months included any alcohol-related nights spent in a hospital, any alcohol-related nights spent in a treatment facility, any alcohol-related ED visits, any alcohol-related outpatient visits in the prior 3 months,^27^ alcohol-related problems,^21^ medication adherence (high adherence to oral naltrexone, defined as taking oral naltrexone 90 of the past 90 days and medium or low adherence as taking oral naltrexone 0 to 89 days; for extended-release injectable naltrexone, high adherence was defined as receiving 3 extended-release injectable naltrexone injections from the study and medium or low adherence as 0 to 2 injections), and health care costs (eMethods 3 in Supplement 2).

### Statistical Analysis

This study had 80% power to detect a 16% difference between treatment groups in the change between baseline and 3-month follow-up percentage of HDDs in the past 30 days, assuming a standard deviation of 41, 2-sided testing, and an α of .05, with 208 anticipated evaluable participants (those randomized who completed 3-month follow-up) and 248 randomized (intention-to-treat [ITT] participants). Due to the COVID-19 pandemic and higher than expected retention and follow-up, recruitment and enrollment concluded in March 2020, with 248 randomized prior to reaching the original target of 260 randomized. Follow-up data collection for primary and secondary outcomes concluded in July 2020, with 217 evaluable participants.

**Figure 1.**
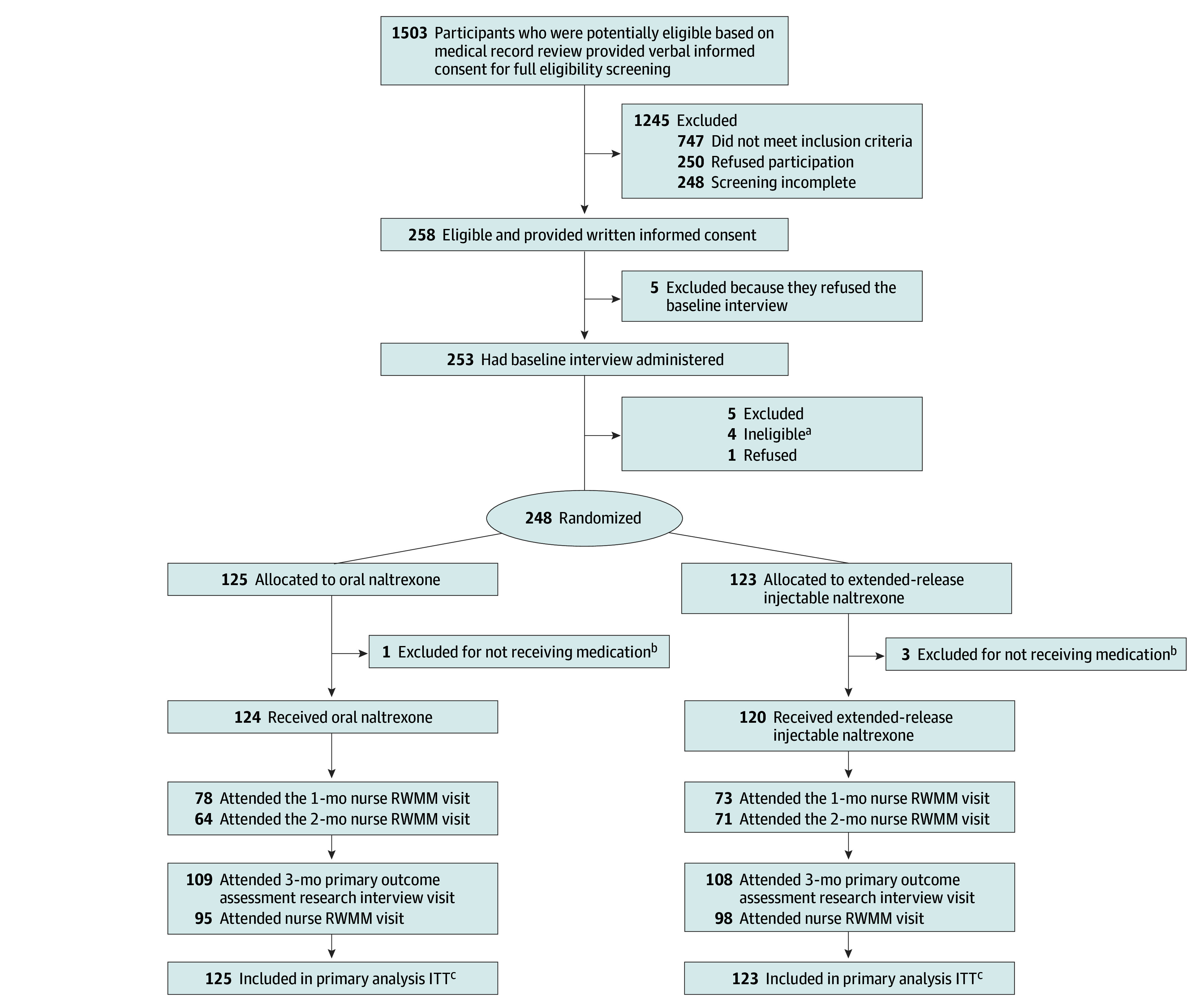
CONSORT Diagram ITT indicates intention to treat; RWMM, real-world medication management. ^a^Four participants were found to be ineligible after the baseline interview (prior to randomization) because they were not fluent in English (n = 1), prescribed anticoagulant medication (n = 1), had a potential future need for opioids (n = 1), and had acute severe psychiatric illness (n = 1). ^b^Four randomized participants did not receive medications due to further health concerns (n = 2) or refusal to receive medication (n = 2). ^c^The ITT population includes all participants who were randomized after baseline assessment.

Participant disposition was summarized in Figure 1. The ITT population included all participants who both completed the baseline assessment and were randomized. The ITT population was used for analysis of the primary outcome and the secondary outcome, with multiple imputation applied only to account for missing data at 3-month follow-up for these 2 outcomes. For imputation, each outcome was associated with participant baseline characteristics using monotone missing data pattern regression with 20 iterations. Rubin rules were applied to combine the results from the 20 imputations, accounting for both within-imputation and between-imputation variability, to produce pooled estimates and correct SEs. A complete case sensitivity analysis was conducted for both the primary and secondary outcome (eTable 2 in Supplement 2). For all other outcomes, multiple imputation was not performed.

Descriptive statistics were computed by treatment group. For statistical inference, 95% CIs and *P* values were presented, based on a 2-sided test with an α less than .05; no adjustment was made for multiple testing. For all outcomes (except cost, which was only analyzed descriptively), multivariable regression methods were used to test the association between treatment group and each outcome, adjusting for randomization stratification factors, sex, and race to improve efficiency. Linear (continuous outcomes) and logistic (binary outcomes) regression models were used as appropriate.

Additionally, we compared self-reported heavy drinking (1 or more HDDs) and alcohol biomarkers as dichotomous variables: phosphatidylethanol (20 ng/mL or greater)^28,29^; GGT (more than 35 U/L; to convert to μkat/L, multiply by 0.0167); and dCDT (1.7% or greater). This comparison is presented as 2 × 2 tables of 1 or more HDDs by alcohol biomarker at the aforementioned thresholds. An exploratory analysis was performed to examine whether the primary outcome analysis (regression models with treatment arm predicting percentage of HDDs in the past 30 days adjusting for sex and race) results would be replicated when the percentage of HDDs in the past 30 days was replaced with different alcohol biomarkers (phosphatidylethanol, GGT, and dCDT) as the outcome. In the oral naltrexone group, we assessed the proportion of participants with detectable 6-β-naltrexol in plasma among those who self-reported high medication adherence. Phosphatidylethanol was not examined as a continuous variable as it was not normally distributed, even with log transformation.

Descriptive statistics characterized frequency and number of participants affected by adverse events (AEs), serious AEs, and AEs by system organ class. Statistical analyses were produced using SAS version 9.4 (SAS Institute).

## Results

Of 1503 patients screened for eligibility, 258 were eligible and enrolled (Figure 1). Of those 258, 9 were found ineligible after enrollment (prior to randomization), and 1 refused randomization after enrollment, resulting in 248 participants randomized (125 to the oral naltrexone group and 123 to the extended-release injectable naltrexone group), forming the ITT population. Of 248 participants, 217 (87.5%) completed 3-month follow-up, including 109 in the oral naltrexone group and 108 in the extended-release injectable naltrexone group. There were no missing data for the baseline stratification variables sex and race. A total of 199 (80.2%) were male, and the mean (SD) age was 49.4 (10.4) years (Table 1).

**Table 1.** Baseline Characteristics of Hospitalized Patients With Alcohol Use Disorder

Characteristic	No. (%)	
	Oral naltrexone (n = 125)	Extended-release injectable naltrexone (n = 123)
**Demographic characteristics** ^a^		
Sex^b^		
Female	25 (20.0)	24 (19.5)
Male	100 (80.0)	99 (80.5)
Race		
Black	64 (51.2)	61 (49.6)
White	54 (43.2)	47 (38.2)
Other race^c^	7 (5.6)	15 (12.2)
Ethnicity		
Hispanic	19 (15.2)	20 (16.3)
Non-Hispanic	106 (84.8)	103 (83.7)
Age, mean (SD), y	49.3 (10.5)	49.4 (10.3)
Education		
No high school degree	26 (20.8)	32 (26.0)
≥High school degree	99 (79.2)	91 (74.0)
Never married	57 (45.6)	68 (55.3)
Unhoused (≥1 night in past 3 mo)	58 (46.4)	58 (47.2)
Primary health insurance		
Medicaid	80 (64.0)	88 (71.5)
Medicare	34 (27.2)	24 (19.5)
Private	8 (6.4)	10 (8.1)
Uninsured	3 (2.4)	1 (0.8)
Incarcerated in past 3 mo, No./total No. (%)	16/125 (12.8)	11/121 (9.1)
**Health and substance use characteristics**		
Charlson Comorbidity Index score, median (IQR)^d^	1.0 (1.0-3.0)	1.0 (0-3.0)
Pain intensity, median (IQR)^e^	6.0 (4.0-8.0)	6.0 (4.0-8.0)
Pain interference, median (IQR)^f^	6.0 (3.0-8.0)	5.0 (3.0-8.0)
Smoking status, No./total No. (%)		
Current smoker	74/118 (62.7)	81/116 (69.8)
Former smoker	13/118 (11.0)	16/116 (13.8)
Never smoker	31/118 (26.3)	19/116 (16.4)
Alcohol use disorder severity by AUDADIS		
Mild	3 (2.4)	6 (4.9)
Moderate	6 (4.8)	8 (6.5)
Severe	116 (92.8)	109 (88.6)
Alcohol abstinent for 7 d prior to hospitalization^g^	10 (8.0)	5 (4.1)
World Health Organization drinking risk level		
Low risk	14 (11.2)	16 (13.0)
Moderate risk	9 (7.2)	11 (8.9)
High risk	24 (19.2)	15 (12.2)
Very high risk	78 (62.4)	81 (65.9)
PACS craving score, mean (SD)^h^	15.4 (8.2)	15.6 (8.4)
Moderate or severe anxiety symptoms^i^	67 (53.6)	65 (52.8)
Moderate or severe depressive symptoms^j^	71 (56.8)	78 (63.4)
Probable diagnosis of PTSD by PC-PTSD, No./total No. (%)	63/124 (50.8)	53/123 (43.1)
General health status		
Very dissatisfied or dissatisfied	74 (59.2)	78 (63.4)
Neither satisfied nor dissatisfied	23 (18.4)	17 (13.8)
Satisfied or very satisfied	28 (22.4)	28 (22.8)
Overall quality of life		
Very poor or poor	52 (41.6)	52 (42.3)
Neither poor nor good	38 (30.4)	39 (31.7)
Good or very good	35 (28.0)	32 (26.0)
WHOQOL BREF, mean (SD)^k^		
Enviromental health score^k^	53.0 (17.4)	52.0 (19.6)
Physical health score^k^	53.1 (19.3)	49.8 (19.3)
Pyschological health score^k^	54.3 (19.7)	51.7 (21.7)
Social health score^k^	49.9 (24.8)	49.7 (26.3)
EQ-5D Index score, mean (SD)^l^	0.57 (0.3)	0.56 (0.3)
EQ VAS score, mean (SD)^m^	52.6 (21.8)	52.3 (22.4)
Injury requiring medical attention in past 3 mo	39 (31.2)	42 (34.1)
**Psychosocial characteristics**		
Readiness to change		
Precontemplation	1 (0.8)	6 (4.9)
Contemplation	15 (12.0)	9 (7.3)
Determination	16 (12.8)	17 (13.8)
Action	93 (74.4)	91 (74.0)
Reasons for quitting, median (IQR)^n^		
Extrinsic motivation	1.6 (0.8-2.4)	1.8 (0.9-2.7)
Health concerns	3.0 (2.0-3.8)	3.0 (2.3-4.0)
Intrinsic motivation	3.0 (2.2-3.7)	3.3 (2.3-4.0)
Legal concerns	1.0 (0-2.7)	1.3 (0-3.0)
Self-concept	3.0 (2.0-4.0)	3.3 (2.3-4.0)
Social influence	2.0 (1.2-3.0)	2.2 (1.2-3.3)
Alcohol use motives, mean (SD)^o^		
Coping	2.9 (0.7)	2.8 (0.8)
Enhancement	2.8 (0.8)	2.8 (0.8)
Social	2.6 (0.8)	2.6 (0.9)
Stressful Life Events Scale, median (IQR)^p^	4.0 (2.0-6.0)	4.0 (2.0-6.0)
Perceived Stress Scale 4, mean (SD)^q^	8.3 (2.7)	8.6 (3.1)
AUD treatments in the past 3 mo		
Any pharmacotherapy for AUD	21 (16.8)	19 (15.4)
Oral naltrexone taken for AUD	15 (12.0)	11 (8.9)
Extended-release injectable naltrexone taken for AUD	1 (0.8)	3 (2.4)
Acamprosate taken for AUD	8 (6.4)	8 (6.5)
Disulfiram taken for AUD	2 (1.6)	0
Participated in mutual help group	37 (29.6)	40 (32.5)

Abbreviations: AUD, alcohol use disorder; AUDADIS, Alcohol Use Disorder and Associated Disabilities Interview Schedule; EQ-5D, European Quality of Life Questionnaire 5-Dimensation; EQ VAS, European Quality of Life Questionnaire Visual Analogue Scale; PACS, Penn Alcohol Craving Scale; PC-PTSD, Primary Care Post-Traumatic Stress Disorder Screen; PTSD, posttraumatic stress disorder; WHOQOL BREF, World Health Organization Quality of Life Questionnaire Brief.

^a^ Demographic characteristics, including race and ethnicity, were self-reported by participants.

^b^ One participant reported being a transgender woman; the participant was categorized as male.

^c^ The other race category includes Asian, American Indian or Alaska Native, Native Hawaiian or Other Pacific Islander, or more than 1 race; these were combined because of low numbers.

^d^ Scores range from 0 to 33, with higher scores representing a greater burden of illness and poorer health status.

^e^ Scores range from 0 to 10, with higher scores representing greater pain intensity.

^f^ Scores range from 0 to 10, with higher scores representing greater pain interference with daily activities.

^g^ Derived from Timeline Followback calendar method^17^ and indicates if the participant did not drink any alcohol in the 7 days prior to baseline hospitalization.

^h^ Scores range from 0 to 30, with higher scores representing higher craving to drink alcohol in the past week.

^i^ Anxiety was measured using the General Anxiety Disorder-7 with a cutoff point of 10 points or higher.

^j^ Depression was measured using the Patient Health Questionnaire-8, with a cutoff point of 10 points or higher.

^k^ Scores range from 0 to 30, with higher scores representing better health-related quality of life.

^l^ Scores range from −0.11 to 1.00, with higher scores representing better self-reported health.

^m^ Scores range from 0 to 100, with higher scores representing better overall health.

^n^ Scores range from 0 to 4, with higher scores representing greater level of motivation on that dimension.

^o^ Scores range from 0 to 4, with higher scores indicating higher frequency of alcohol use for reasons within the subscale.

^p^ Scores range from 0 to 12, with higher scores indicating experiencing more stressful life events in the past 12 months.

^q^ Scores range from 0 to 16, with higher scores indicating higher perceived stress.

### Primary Outcome

**Figure 2.**
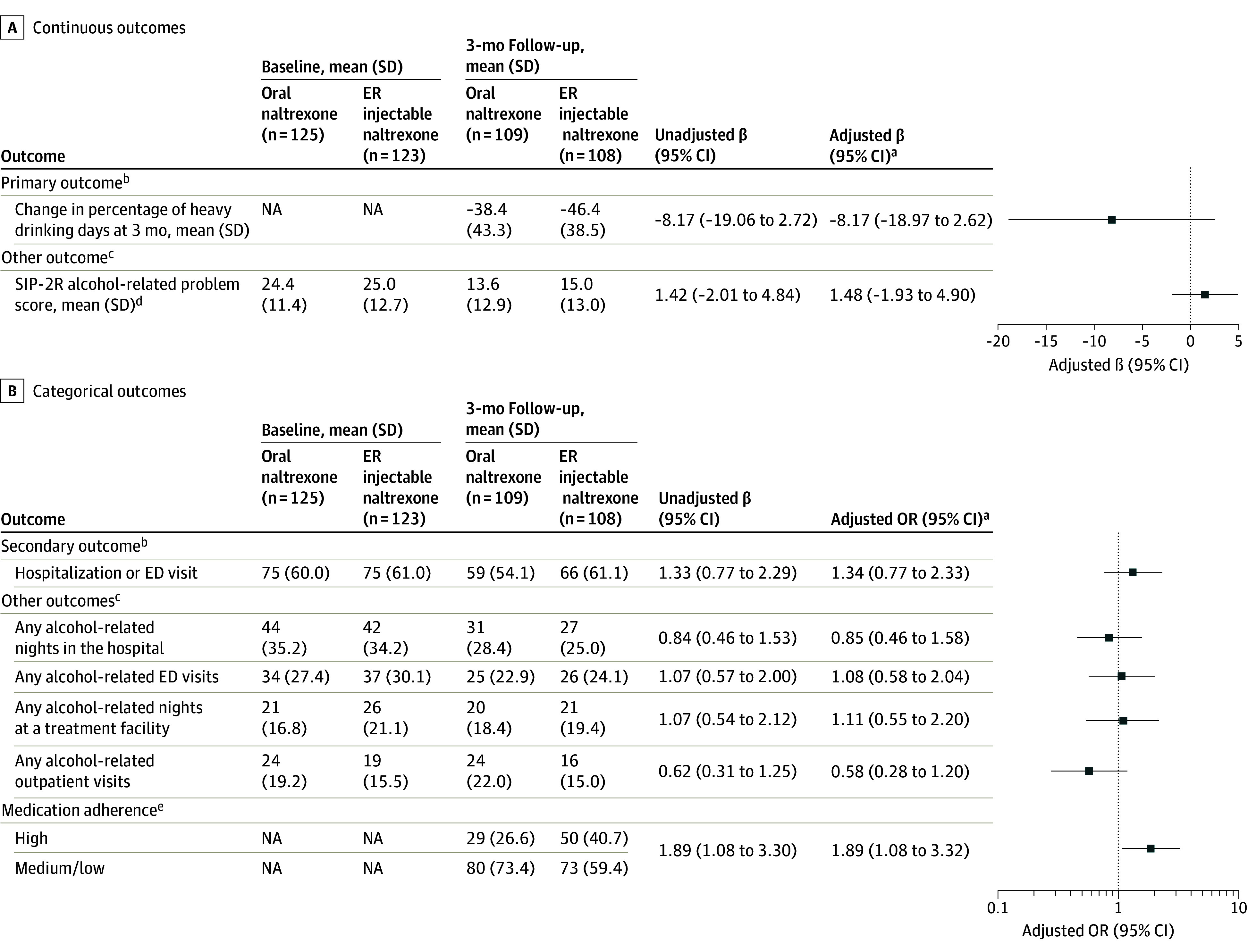
Outcomes for Oral vs Extended-Release (ER) Injectable Naltrexone Among Patients With Alcohol Use Disorder (N = 248) Baseline data were not collected for percentage of HDDs in the past 30 days and medication adherence. The reference group for unadjusted and adjusted βs was the oral naltrexone group. Heavy drinking was defined as 5 or more drinks for men and 4 or more drinks for women. ED indicates emergency department; HDD, heavy drinking day; NA, not applicable; OR, odds ratio; SIP-2R, Short Inventory of Problems Revision 2. ^a^Models adjusted for sex and race. ^b^Models analyzing the primary outcome (change in percentage of HDDs in the prior 30 days) and the secondary outcome (hospitalization or ED visit) use the intention-to-treat population (all participants who completed baseline and were randomized; N = 248) with multiple imputation used to impute outcomes if missing at 3-month follow-up. Baseline characteristics used to carry out the multiple imputation for both the primary and secondary outcomes were the following: treatment group, sex, race (dichotomized as Black or other race [American Indian or Alaskan Native, Asian, Native Hawaiian or Pacific Islander, White, or more than 1 race]), age, Charlson Comorbidity Index score, any acute care utilization in the past 3 months, unhoused, depressive symptoms, anxiety symptoms, any lifetime substance (cannabis, cocaine, amphetamine-type stimulants, opioid, sedatives, hallucinogens, or inhalants) use, abstinence in the 7 days prior to baseline hospitalization (from Timeline Followback calendar method^17^), World Health Organization drinking risk levels, alcohol use motives (specifically, coping motive), and Alcohol, Smoking, and Substance Involving Screening Test substance-specific risk level scores for cannabis and cocaine and highest level of risk for any substance in the Alcohol, Smoking, and Substance Involving Screening Test. ^c^Models analyzing other outcomes use the evaluable population (participants randomized with evaluable outcome data [n = 217]). No other outcomes were imputed if missing. ^d^The SIP-2R theoretical range is between 0 and 45, with a higher score indicating more alcohol-related consequences. ^e^High adherence to ER injectable naltrexone was defined as administration of injectable naltrexone by the study nurse 3 times (this adherence measure did not require research follow-up assessment). Medium or low adherence was defined as 1 or 2 injections. For oral naltrexone, high adherence was defined as participant self-report of taking oral naltrexone for 90 of the past 90 days at the 3-month follow-up assessment, and medium or low adherence was defined as participant self-report of taking oral naltrexone for 0 to 89 of the past 90 days at the 3-month follow-up assessment. A total of 232 patients were evaluable for medication adherence.

The mean percentage of HDDs in the 30 days prior decreased in both treatment arms from baseline to 3-month follow-up. Specifically, in the oral naltrexone arm, the mean (SD) percentage of HDDs in the past 30 days at baseline was 66.7% (31.5) and at follow-up was 27.4% (37.0). In the extended-release injectable naltrexone group, the mean (SD) percentage of HDDs in the past 30 days at baseline was 70.7% (31.5) and at follow-up was 23.8% (32.8) (Table 2). At 3-month follow-up, the mean percentage of HDDs in the past 30 days was reduced in both groups (oral naltrexone: baseline, 66.7% HDDs; 3-month follow-up, 27.4% HDDs; difference, −38.4 percentage points; 95% CI, −125.0 to 48.2; extended-release injectable naltrexone: baseline, 70.7% HDDs; 3-month follow-up, 23.8% HDDs; difference, −46.4 percentage points; 95% CI, −123.4 to 30.6; *P* = .14) (Figure 2). In adjusted analysis, the difference in the change in percentage of HDDs in the past 30 days between treatment groups was −8.17 percentage points (95% CI, −18.97 to 2.62), with the extended-release injectable naltrexone group experiencing greater reduction in percentage of HDDs compared with oral naltrexone, although this difference between groups was not significant (Figure 2).

**Table 2.** Baseline and 3-Month Follow-Up Alcohol Use, Other Substance Use and Risk, Laboratory Tests, and Health Care Utilization Among Patients With Alcohol Use Disorder

Variable	No./total No. (%)			
	Baseline (N = 248)		3-mo Follow-up (n = 217)	
	Oral naltrexone (n = 125)	Extended-release injectable naltrexone (n = 123)	Oral naltrexone (n = 109)	Extended-release injectable naltrexone (n = 108)
HDDs in past 30 d				
Median (IQR), No.	22.0 (13.0-30.0)	25.0 (14.0-30.0)	1.0 (0-16.0)	1.5 (0-12.0)
Mean (SD), No.	20.0 (9.5)	21.2 (9.4)	8.2 (11.1)	7.1 (9.9)
Median (IQR), %	73.3 (43.3-100)	83.3 (46.7-100)	3.3 (0-53.3)	5.0 (0-40.0)
Mean (SD), %	66.7 (31.5)	70.7 (31.5)	27.4 (37.0)	23.8 (32.8)
Any lifetime substance use^a^	109/125 (87.2)	117/123 (95.1)	94/108 (87.0)	104/108 (96.3)
ASSIST substance-specific risk score^b^				
Low risk (0-3)	57/125 (45.6)	53/123 (43.1)	62/108 (57.4)	54/108 (50.0)
Moderate risk (4-26)	63/125 (50.4)	59/123 (48.0)	40/108 (37.0)	51/108 (47.2)
High risk (≥27)	5/125 (4.0)	11/123 (8.9)	6/108 (5.6)	3/108 (2.8)
Past 3-mo substance use^c^				
Cannabis	61/125 (48.8)	62/123 (50.4)	36/108 (33.3)	36/108 (33.3)
Cocaine	27/125 (21.6)	37/123 (30.1)	16/108 (14.8)	13/108 (12.0)
Amphetamine-type stimulants	1/125 (0.8)	2/123 (1.6)	2/108 (1.9)	0/108
Opioids	5/125 (4.0)	8/122 (6.6)	3/108 (2.8)	1/107 (0.9)
Sedatives	3/125 (2.4)	7/123 (5.7)	1/108 (0.9)	4/108 (3.7)
Hallucinogens	0/125	1/123 (0.8)	0/108	0/108
Inhalants	1/125 (0.8)	0/123	1/108 (0.9)	0/108
Laboratory testing				
GGT				
Total, No.	NA	NA	79	84
Median (IQR), U/L	NA	NA	61.0 (29.0-187.0)	51.0 (26.0-109.5)
dCDT				
Abnormal level (≥1.7%)	NA	NA	40/77 (51.9)	34/80 (42.5)
Normal level (≤1.6%)	NA	NA	37/77 (48.1)	46/80 (57.5)
Phosphatidylethanol				
<20 ng/mL	NA	NA	17/80 (21.3)	22/82 (26.8)
≥20 ng/mL	NA	NA	63/80 (78.8)	60/82 (73.2)
ALT, median (IQR), U/L				
Total, No.	125	123	86	92
Median (IQR), U/L	32.0 (17.0-55.0)	33.0 (19.0-48.0)	24.0 (16.0-38.0)	24.0 (16.0-35.5)
AST, median (IQR), U/L				
Total, No.	125	123	86	92
Median (IQR), U/L	45.0 (29.0-69.0)	40.0 (25.0-67.0)	29.5 (20.0-47.0)	27.0 (20.0-46.5)
ALT abnormal (5-fold upper limit or greater)	NA	NA	0/86	2/92 (2.2)
AST abnormal (5-fold upper limit or greater)	NA	NA	0/86	4/92 (4.3)
Health care utilization				
Any nights spent in the hospital	55/125 (44.0)	52/123 (42.3)	45/109 (41.3)	46/108 (42.6)
Any days in emergency department	53/124 (42.7)	57/123 (46.3)	40/109 (36.7)	41/108 (38.0)
Any nights at an inpatient treatment facility	22/125 (17.6)	27/123 (22.0)	21/109 (19.3)	21/108 (19.4)
Outpatient visits	52/125 (41.6)	40/123 (32.5)	61/109 (56.0)	47/107 (43.9)

Abbreviations: ALT, alanine aminotransferase; ASSIST, Alcohol, Smoking, and Substance Involving Screening Test; AST, aspartate aminotransferase; dCDT, disialo-carbohydrate–deficient transferrin; GGT, γ-glutamyltransferase; HDD, heavy drinking day (defined as 5 or more drinks for men and 4 or more drinks for women); NA, not applicable.

SI conversion factors: To convert GGT to μkat/L, multiply by 0.0167; ALT to μkat/L, multiply by 0.0167; AST to μkat/L, multiply by 0.0167.

^a^ Self-report of any lifetime use of cannabis, cocaine, amphetamine-type stimulants, inhalants, sedatives, hallucinogens, and opioids.

^b^ The ASSIST substance-specific score creates a risk score for each substance for each participant. Participants are categorized into 1 of 3 categories (low, moderate, or high risk) based on the highest score they received among all substances. Participants with no lifetime use at baseline and no use in the prior 3 months at follow-up were scored as 0.

^c^ Data come from the ASSIST measure.

### Alcohol Biomarkers

Phosphatidylethanol of 20 ng/mL or greater was found in 123 of 162 participants (75.9%) who had phosphatidylethanol results at 3-month follow-up, with phosphatidylethanol of 20 ng/mL or greater in 63 of 80 in the oral naltrexone group (78.8%) and 60 of 82 in the extended-release injectable naltrexone group (73.2%) (Table 2). More than three-quarters of those with phosphatidylethanol results (125 of 162 [77.2%]) had phosphatidylethanol values consistent with their self-report of heavy drinking (eTable 3 in Supplement 2). A minority of the sample (35 of 162 [21.6%]) had phosphatidylethanol of 20 ng/mL or greater (a value consistent with averaging 2 to 4 drinks per day for several days per week) while reporting no HDDs at follow-up, suggesting possible underreporting of heavy drinking. In adjusted regression models with treatment arm predicting each alcohol biomarker (phosphatidylethanol, dCDT, and GGT) separately (instead of self-reported percentage of HDDs in the past 30 days), results remained consistent with primary outcome analysis (eTable 4 in Supplement 2).

### Secondary Outcome

At baseline, 75 of 125 in the oral naltrexone arm (60.0%) and 75 of 123 in the extended-release injectable naltrexone (61.0%) reported a hospitalization or ED visit in the prior 3 months (Figure 2). At follow-up, 59 of 109 in the oral naltrexone arm (54.1%) and 66 of 108 in the extended-release injectable naltrexone (61.1%) reported a hospitalization or ED visit in the prior 3 months. We did not detect a difference in the odds of hospitalization or ED visit in adjusted analyses (adjusted odds ratio, 1.34; 95% CI, 0.77-2.33) for extended-release injectable naltrexone vs oral naltrexone. The comparison between participants’ hospital medical records and self-reported ED visit or hospitalization revealed consistent findings between these 2 sources of utilization data (eTable 1 in Supplement 2).

### Other Outcomes

In adjusted models, the odds of alcohol-related health care utilization outcomes at 3-month follow-up were not statistically significant by treatment group (Figure 2). Mean Short Inventory of Problems Revision 2 score (alcohol-related problems) was lower in both treatment groups at follow-up compared with baseline; the difference between groups was not significant. Only 29 of 109 participants (26.6%) had high adherence to naltrexone in the oral naltrexone group compared with 50 of 123 participants (40.7%) in the extended-release injectable naltrexone group. In adjusted analysis, the odds of high medication adherence were 1.89-fold (95% CI, 1.08-3.32) higher for extended-release injectable naltrexone compared with oral naltrexone. Only 5 of 26 participants who self-reported high oral naltrexone adherence and had 6-β-naltrexol test results did not have 6-β-naltrexol detectable in their plasma (data not shown).

Median (IQR) health care costs (inclusive of study medication and nurse time, clinical laboratory testing, and self-reported health care utilization) during the 3-month study duration was $3925.44 ($1142.06-$17 108.49) per participant for the overall sample. The median (IQR) cost was lower in the oral naltrexone group ($1630.17 [$327.03-$17 367.85]) than the extended-release injectable naltrexone group ($5208.29 [$3397.89-$16 849.13]).

### AEs

Serious AEs related or possibly related to study medication were rare, experienced by 6 patients (2.4%; in the oral naltrexone group, suicide attempt, alcohol withdrawal, palpitations, and urinary tract infection; in the extended-release injectable naltrexone group, suicide attempt and injection site abscess) (eTable 5 in Supplement 2).

## Discussion

The ADOPT comparative effectiveness study sought to determine if an extended-release formulation of an AUD medication (extended-release injectable naltrexone) resulted in less alcohol consumption compared with daily oral medication (oral naltrexone) when initiated in hospitalized patients. As in other studies,^30,31,32^ both naltrexone formulations reduced HDDs; however, we did not detect a difference in reduction of HDDs between the oral naltrexone and extended-release injectable naltrexone groups. Additionally, acute and alcohol-related health care utilization were not significantly different by treatment arm.

This study elucidated the real-world comparative effectiveness of treatment between 2 formulations of an effective AUD medication initiated in the hospital, a comparison heretofore not examined, to our knowledge. This question is clinically pragmatic as hospitalization presents an opportunity to deliver either treatment. Importantly, although the active ingredient in oral naltrexone and extended-release injectable naltrexone is the same, medication cost, mode of administration, and dosing frequency vary. In this study, the hypothesized benefits of the extended-release medication were not realized. Nonetheless, both medications saw substantial reductions in percentage of HDDs in the past 30 days from baseline to 3-month follow-up.

Poorer adherence with oral naltrexone compared with extended-release injectable naltrexone has been found previously.^30,33,34,35^ By definition, individuals administered extended-release injectable naltrexone have medication in their system for an extended time. Conversely, it is well-known why people may not adhere to daily pill formulations.^34^ We found higher adherence with extended-release injectable naltrexone, a finding consistent with a secondary analysis of this ADOPT study examining adherence over a 3-month period at a lower threshold (ie, 66% adherence [60 or more days for oral naltrexone and 2 or more injections for extended-release injectable naltrexone]).^36^ In a real-world setting, adherence may improve with flexibility in patient choice of treatment. In clinical practice, the most effective medication may vary based on patient preference, availability, cost, and postdischarge follow-up logistics.

The case to use a potentially more effective but more expensive medication (extended-release injectable naltrexone) was not demonstrated in this comparative effectiveness trial. Although we did not perform a formal cost-effectiveness analysis, we found oral naltrexone, given its reduced costs and similar effectiveness, was the higher-value approach for hospitalized patients with AUD. Although the data suggests this interpretation, patient characteristics, preferences, and insurance may influence the optimal approach for individual patients. For example, patients concerned about adherence to daily medication or with intermittent ambivalence about alcohol cessation may be more confident with a monthly injection.

Notably, the study found it feasible to initiate both naltrexone formulations prior to discharge. By addressing AUD in hospitalized patients, care can potentially yield better overall health outcomes. Additionally, since both medications were similarly effective, decisions on which medication may produce the best patient outcomes may be determined by considering patient preferences, costs, insurance, and access to outpatient follow-up. Finally, given the evidence that AUD medication provided in the hospital helps to reduce HDDs, future trials should assess optimal implementation of this strategy from clinician, patient, and payer perspectives.

### Strengths and Limitations

The study has notable strengths. It tested the effectiveness of 2 approaches to naltrexone delivery, yielding important data for clinical guidelines development. Another strength was the use of medical management to maximize benefits of pharmacological AUD treatment (ie, monthly appointments with the study nurse [D.R.] who used a psychosocial framework focusing on clinically relevant issues, mental well-being, and social and environmental factors that influence AUD).

This study has several limitations. It occurred at a single urban hospital and included a large proportion of men and unhoused individuals, which potentially limits generalizability. Another limitation is the self-report nature of much of the data, which is subject to recall and social desirability bias. Mitigating this concern are alcohol biomarker findings, which bolster results. Effectiveness findings might have been strengthened by a no treatment comparison; however, this was not pursued given naltrexone’s known efficacy and the feasibility implications of adding a third arm.

## Conclusions

In this randomized clinical trial, oral and injectable naltrexone initiated at hospital discharge did not differ in their effectiveness for decreasing HDDs; both medications were associated with alcohol use reductions. Hospitalization represents an excellent opportunity to start AUD pharmacotherapy.

## Notes

Supplement 1. Trial Protocol

Supplement 2. **eMethods 1.** Detailed Methods for the Assessment of Health-Related Characteristics of Participants in the Alcohol Disorder Hospital Treatment (ADOPT) Trial **eMethods 2.** Details of Real-World Medical Management Provided to Participants in the Alcohol Disorder Hospital Treatment (ADOPT) Trial **eMethods 3.** Detailed Methods for the Calculation of Health Care Costs During the 3-Month Study Duration for Participants in the Alcohol Disorder Hospital Treatment (ADOPT) Trial **eTable 1.** Comparison of Self-Reported Acute Health Care Utilization (Hospitalization or Emergency Department Visit) and Data Obtained From Participants’ Electronic Medical Records From the Hospital System at Which the ADOPT Study Occurred **eTable 2.** Sensitivity Complete Case Analysis of the Primary Outcome and the Secondary Outcome for Oral Naltrexone Compared With Injectable Naltrexone Among Patients With Alcohol Use Disorder in the ADOPT Trial **eTable 3.** Self-Reported Heavy Drinking Days (TLFB) and Alcohol Biomarkers (PEth, dCDT, and GGT) Among Participants With Alcohol Use Disorder in the Alcohol Disorder Hospital Treatment (ADOPT) Trial **eTable 4.** Associations Between Treatment Group and Alcohol Biomarkers for Participants With Alcohol Use Disorder in the ADOPT Trial: Exploratory Analyses Replacing the Primary Outcome (Self-Reported %HDDs) With PEth, dCDT, GGT **eTable 5.** Adverse Events and Serious Adverse Events in the ADOPT Trial
eReferences.

Supplement 3. Data Sharing Statement
