# Supplementary material for: Oral vs Extended-Release Injectable Naltrexone for Hospitalized Patients With Alcohol Use Disorder: A Randomized Clinical Trial
Source: JAMA Intern Med. 2025 Apr 21;185(6):635–45. doi: 10.1001/jamainternmed.2025.0522 (PMC12013356; doi:10.1001/jamainternmed.2025.0522)
Supplement: Supplement 1. — Trial Protocol [file jamainternmed-e250522-s001.pdf]

Boston University Medical Campus

Alcohol Disorder hosPital Treatment (ADOPT) Study Protocol

Principal Investigator: Richard Saitz, MD, MPH

NIH/NIAAA Award Number: R01AA021335
IRB Protocol Number: H-32911

Protocol Version: May 12, 2021

ADOPT PROTOCOL CONTENTS

ADOPT PROTOCOL CONTENTS 2
1. INTRODUCTION 6
1.1. SUMMARY 6
1.2. SIGNIFICANCE 6
1.2.A. WHY COMPARE XR-NTX AND PO-NTX? 7
2. OVERVIEW OF STUDY DESIGN 7
2.1. STUDY AIMS 7
2.2. STUDY HYPOTHESIS 7
2.3. STUDY OUTCOMES 8
2.4. STUDY DESIGN 8
2.5. STUDY SITES 10
2.7. PARTICIPANT ELIGIBILITY 10
2.8. EXCLUSION CRITERIA 10
2.9. RECRUITMENT GOALS 11
3. INTERVENTION AND CONTROL 11
3.1. METHODS OVERVIEW 11
3.2. RANDOMIZATION 11
3.3. INTERVENTION 12
3.3.A. XR-NTX 12
3.3.B. PO-NTX 12
3.3.C. MEDICAL MANAGEMENT 13
3.4. SCHEDULE OF DATA COLLECTION 16
3.4.A. VISIT WINDOWS 16
3.5. DATA SOURCES 17
3.5.A. URINE 17
3.5.B. BLOOD 17
3.6. OBSERVATIONAL COHORT 19

4. Research Assistants (RAs) Procedures 19
4.1. Introduction 19
4.2. Recruitment 20
4.2.a. Recruitment Process Overview 20
4.2.b. Preparing to Screen 21
4.2.c. Screening 21
4.3. Informed Consent 22
4.3.a. Observational Cohort 22
4.3.B. Medical Management Recordings 22
4.4. Interview Protocol 23
4.4.a. Participant Interactions 23
4.4.b. Compensation 23
4.4.c. Baseline Assessment Procedures 23
4.4.d. Randomization 25
4.4.e. Follow-up Assessments 25
4.5. Participant Retention 26
4.5. Inpatient Medical Service Responsibilities 26
4.6.a. Interacting with Clinical Staff 27
4.6.b. Using the EMR 27
4.7. Office Responsibilities 27
4.7.a Quality Assurance 27
5. Investigational Pharmacy Services (IPS) 27
5.1. XR-NTX 28
5.2. PO-NTX 29
6. Assessments: Measurements and Data Collection Process 29
6.1. Baseline Assessment 29
6.2. Follow-up Assessment 29
6.3. Quality Assurance 29

7. Participant Safety 30
7.1. Protocol Review and Study Monitoring 30
7.3. Potential Risks 30
7.3.a. Psychological stress 30
7.3.b. Loss of Confidentiality 30
7.3.c. Phlebotomy Associated Risks 31
7.3.d. Biohazard Containment 31
7.3.e. Psychological Stress from Medication 31
7.3.f. Genetic Risks 31
7.3.g. Medication Risks 32
7.4. Risk Assessment 33
7.5. Adverse Event Reporting and Discontinuation of Study Treatment 33
7.5.a. Adverse Events 33
7.5.b. Recording of Unanticipated Problems and Adverse Events 36
7.5.c. Reporting of Unanticipated Problems to the IRB 36
7.5.d. Reporting of Adverse Events to the IRB 36
7.5.e. Blinding 37
7.6. Stopping Rules 37
8. Data Management 37
8.1. Data Collection 37
8.2. Quality Control Process 38
8.5. Data Security and Confidentiality 38
8.5.a. Coded Data 38
8.5.b. SSL Encryption 38
8.6. Web Systems 39
8.6.A. Screening System 39
8.6.B. Tracking System 39
8.6.C. REDCap System 40

9. STATISTICAL ANALYSIS 40
9.1. OUTCOMES 40
9.2. HYPOTHESES 40
9.3. PRELIMINARY ANALYSES 40
10. STAFF TRAINING 41
11. NIAAA 41
11.1. FUNDING STATEMENTS 41
11.2. CLINICALTRIALS.GOV REGISTRATION 41
Appendix 1.0: statistical analysis plan 42

1. Introduction

1.1. Summary

As many as 40% of general medical hospital admissions are linked to alcohol, and alcohol use disorder (AUD) is a risk factor for readmission (a marker of poor quality of care for which hospitals are financially penalized). Yet most inpatients with AUD receive no effective treatment, leaving them vulnerable to both alcohol-specific consequences, and the deleterious effects of untreated AUD on medical conditions and related healthcare utilization. Pharmacotherapy for AUD (oral tablet naltrexone, PO-NTX; extended-release injectable naltrexone, XR-NTX) has efficacy but unlike treatments for other medical conditions routinely started at hospital discharge it is rarely prescribed. In addition, little is known about the effectiveness of pharmacotherapy under real-world conditions compared to other clinically available options, and clinicians have little evidence upon which to base decisions to prescribe a costly (per dose) monthly injection or a daily tablet (that may be less adhered to). Therefore, this study aims to test the effectiveness of beginning XR-NTX and PO-NTX for AUD in medical inpatients at the time of hospital discharge in a pragmatic comparative effectiveness randomized trial. In the Alcohol Disorder hosPital Treatment (ADOPT) study, inpatients will receive counseling in the hospital and at follow-up that is feasible in medical settings, and be randomly assigned to monthly XR-NTX or daily PO-NTX. The primary outcome is percent heavy drinking days; additional outcomes of interest include alcohol consequences, medical healthcare utilization (hospital and emergency department), AUD treatment utilization, and costs. The main hypothesis is that XR-NTX will have greater effectiveness than PO-NTX on drinking, consequences and utilization. Cost-effectiveness analysis will compare the costs and outcomes of the two alternatives. Patient factors that moderate effectiveness will be explored. The study is innovative in its design as a pragmatic comparative effectiveness trial, and because it addresses a question that is unanswered but highly relevant. It is timely in that a national accreditation body recently implemented quality performance measures of AUD treatment at hospital discharge despite the absence of evidence for effectiveness in that circumstance. The significance of the study is that results will provide guidance for clinicians and policymakers regarding initiation of pharmacotherapy for AUD for patients at high risk for costly health consequences who largely receive no effective care at the time of hospital discharge.

1.2. Significance

Addressing AUD in medical hospital inpatients is rarely done, and there is great potential to reduce morbidity and cost. AUD is common and costly in the general population, and is even more common and costly among hospitalized medical patients. Yet AUD is often not recognized among patients in medical settings and even when it is identified, patients rarely receive any effective treatments. The risk for re-hospitalization with its attendant costs and morbidity increases substantially. The risk and cost for preventable re-hospitalization from any cause are currently receiving national attention. As such, studies of effective treatments for AUD in this setting are significant.

AUD pharmacotherapy is dramatically under-utilized; critical questions of clinical relevance to patients, clinicians and policymakers remain unanswered about real world effectiveness and costs in medical settings. Medications, including naltrexone (NTX), have efficacy for AUD, particularly when prescribed to patients with several days' abstinence, as would be the case in inpatients. But they are very underutilized, even in settings where prescribers are in great supply like general hospitals. One of the main reasons physicians do not prescribe NTX is that they perceive it to have low effectiveness. A pragmatic study comparing alternatives available to clinicians in a medical setting showing clinically important effects could lead to wider dissemination. In addition to the need for evidence of real world effectiveness of NTX, clinicians and patients are faced with a choice between a costly monthly injection ($1100 retail, $869 wholesale, acquisition cost $695) and a less costly daily tablet ($104 per month). No efficacy randomized controlled trial (RCT) has compared these alternatives; however, an efficacy study would not be as informative as a pragmatic (effectiveness) trial, because the question of relevance is how these will be taken with attention to adherence and follow-up that is feasible in medical settings.

If pharmacotherapy is effective, and if XR-NTX is more effective than PO-NTX, the study results will support adoption of pharmacotherapy, changing existing clinical practices.

1.2.A. Why Compare XR-NTX and PO-NTX?

NTX has known efficacy for AUD. PO-NTX is taken once a day or XR-NTX is taken once a month and both are well-tolerated. Other medications approved for AUD are: acamprosate, which is three times a day, and disulfiram, which has many contraindications and is only efficacious when given with close monitoring.

Both XR-NTX and PO-NTX are treatment options but one costs much more than the other (for the medication alone) and medication adherence likely differs. Adherence is associated with efficacy. XR-NTX holds particular promise in this regard and is appealing for use at hospital discharge because of the potential time gap before follow-up with a clinician or treatment program. Baser et al. published a retrospective analysis based on claims data that found use of an FDA-approved medication for alcohol dependence (AD) was associated with fewer admissions and (despite the cost of medication itself) lower total healthcare costs ($8,134 vs. $11,677) for 6 months following medication or non-medication alcohol treatment initiation (analyses used propensity score matched risk adjustment). Total costs were similar for XR-NTX, PO-NTX and disulfiram, but significantly higher for acamprosate. Hospital admissions were significantly lower in the medication versus no medication groups and in the XR-NTX treated group compared to all other medications. The XR-NTX group also had significantly higher refill rates and days of medication received ("persistence") (good proxies for adherence since if medication cannot be taken if it is not refilled). Six-month "continuous medication possession" was 21% for XR-NTX but only 11% for PO-NTX. Similarly, Kranzler et al. found that only 14% of patients who filled 1 PO-NTX prescription filled prescriptions for ≥80% of a 6-month treatment period, and such persistence was associated with less high-cost healthcare utilization. These are rates one can anticipate in an effectiveness study such as the current proposal (in contrast to 85% rates found in efficacy studies). And regardless of refills, XR-NTX has early effects and remains effective for a month with no further dosing. Another study of Aetna insurance claims had similar findings, and a third national claims analysis found medication to be associated with less alcohol-related health utilization with greater effects for XR-NTX. In Massachusetts, the Massachusetts Behavioral Health Partnership (MBHP) manages substance abuse services for Medicaid. MBHP studied XR-NTX (and no counseling) in 40 adults (6 women) with AD receiving acute treatment services (medical detoxification); all but one had prior detoxification episodes and 40% had >5. Of the 40, 32 received >1 injection and 47% received a second one. In this case series, the 30-day readmission rate was 5% (2 of 40) (compared to 33% for other plan members in that circumstance).

2. Overview of Study Design

2.1. Study Aims

The specific aims of this pragmatic (effectiveness, not efficacy) RCT are to compare initiating extended-release injectable naltrexone (XR-NTX) or oral tablet naltrexone (PO-NTX) at the time of discharge from a medical hospitalization for patients with an alcohol use disorder on: 1) alcohol consumption and consequences, and 2) acute healthcare utilization (including hospital readmission and emergency visits) and cost-effectiveness. 3) In a 3rd, exploratory aim, we will assess moderators of medication effects including demographic, behavioral, and genetic factors. All receive real world medical management monthly, and all complete 3-month research interviews and contribute utilization data for 1 year. Although the active ingredient is the same the medications differ in costs, mode of administration and dosing frequency, which will likely lead to different real world effectiveness and cost-effectiveness.

2.2. Study Hypothesis

The study will test the main hypotheses that XR-NTX will have greater effectiveness (and cost-effectiveness) than PO-NTX on drinking, consequences and healthcare utilization. In addition, factors (e.g., sex, abstinence) may be associated with medication effectiveness.

2.3. Study Outcomes

Aim#1 is to determine the effectiveness of XR- vs. PO-NTX for alcohol consumption and consequences. Hypothesis #1 is that participants randomized to treatment with XR-NTX will have lower alcohol consumption and consequences than those randomized to PO-NTX. Aim #2 is to determine effectiveness for acute healthcare utilization (dichotomous emergency visit or hospitalization over 3 months). Hypothesis #2 is that participants randomized to treatment with XR-NTX will have lower acute healthcare utilization than those randomized to PO-NTX. The primary outcome of the study is percent heavy drinking days (%HDDs) over the past 30 days assessed at 3 month follow-up by the TLFB. %HDDs is the most likely (and most sensitive) to be affected by NTX and is clinically important (any reduction means less risk of harm). We chose self-report because biological testing is not sufficiently valid for detecting drinking levels and changes of clinical importance. The self-report tool is valid, particularly so when staff are well trained, and when the context for the participant is: they are informed they are being tested for consumption (breath alcohol and CDT), there are no consequences related to consumption levels, and that results are being recorded confidentially. Also, psychosocial intervention is the same in the 2 groups, minimizing and equalizing social desirability bias.

Other hypothesis #1 outcomes are 90-day %HDDs, percent days any drinking, alcohol consequences (SIP score), 30-day drinks/drinking day, drinking risky amounts (>7 for women/elderly on average per week [>14 for men] or >3 or >4, respectively, in a day), percentage of participants with no heavy drinking days (PSNHDDs), 113 and the biomarkers serum disialo-carbohydrate deficient transferrin (CDT) and gamma-glutamyl transferase (GGT) (continuous and dichotomized). Although PSNHDDs is designated as acceptable by the FDA for phase 3 trials we did not choose it as the primary outcome because an important difference in %HDDs may be present when there is no difference in PSNHDDs. Though not a main focus, we examine adverse.

The secondary outcome is any acute hospital utilization (emergency department visit or inpatient stay) over the past 90 days assessed at 3-month follow-up (Form 90). Self-report for short-term high impact, memorable health utilization such as emergency and hospital stays is valid and can measure utilization at any site. Using the clinical data warehouse at one site, Boston Medical Center (BMC) and statewide data, we will compare self-report to the utilization databases. But, self-report data will remain primary, with databases used to provide a descriptive frame of reference. Other outcomes for hypothesis #2 are number of emergency department visits at 30 and 90 days, any emergency department visits (90 days), number of hospitalizations at 30 and 90 days, hospitalizations (90 days), and any emergency department or inpatient stay at 30 days, and all of the noted constructs at 12 months (from the state data).

Additional outcomes of interest: Medication adherence is not an outcome of the intervention of interest per se. But because adherence is likely to be associated with outcome and may be the mechanism by which XR-NTX is more effective than PO-NTX, we will assess whether the participant takes/receives the assigned medication. High adherence to XR-NTX will be defined as administration of XR-NTX by the study nurse 3 times. Medium adherence will be defined as 1 or 2 injections. For PO-NTX, high adherence will be defined as participant self-report of taking PO-NTX for 90 out of the past 90 days at the 3 month study visit, and medium adherers as participant self-report of taking PO-NTX for 30-89 out of the past 90 days at the 3 month study visit (Form 90). We will also assess the presence of detectable 6-β-naltrexol to confirm reports of recent tablet taking. We will also assess adherence to study RW-MM visits and exposure to mutual help (e.g., 12-step) groups and specialty alcohol treatment (Form 90). Self-report of outside specialty treatment will also be assessed using statewide utilization databases but self-report is of primary interest because it captures a wider range of services (e.g., medication, mutual help groups, counseling from non-specialists, and employee assistance programs (EAPs)). Craving has been reduced in prior studies of naltrexone. We will assess this outcome for descriptive purposes but not as a primary analysis.

2.4. Study Design

The study is a randomized, controlled trial (RCT). We will:

- Identify, through screening, hospitalized patients with an alcohol use disorder
- Enroll up to 320 screen-positive participants to randomize 260 and follow them for 3 months
- Randomly assign participants to one of the two study conditions after the baseline assessment:
    - i. Monthly XR-NTX (380 mg) and
    - ii. Daily PO-NTX (50 mg, up to 100 mg if heavy drinking continues).
- Assess all participants at study entry and 3 months later to determine alcohol consumption and consequences, including healthcare utilization.
- Examine the effectiveness of initiating XR-NTX or PO-NTX at the time of discharge from a medical hospitalization for patients with an alcohol use disorder on:
    - alcohol consumption and consequences, and
    - acute healthcare utilization (including hospital readmission and emergency visits) and cost-effectiveness.
    - In a 3rd, exploratory aim, we will assess moderators of medication effects including demographic, behavioral, and genetic factors.

Randomization will occur after enrollment, informed consent, completion of baseline assessments, and when it is time for hospital discharge, participants will be randomized to 1 of the 2 study conditions. This methodology will minimize bias by assuring allocation concealment at the time of the baseline interview. Randomization will be stratified by 2 key factors: sex and race (African American/Black vs. not), variables expected to be associated with effectiveness. Stratification will ensure a comparable distribution of sex and race across randomized groups. To ensure balance with respect to the number of participants in each group, the permuted blocks strategy (using random block sizes [of 2 and 4]) will be used.

Study staff will use a web-based system for randomization assignments. Study assessment forms will not indicate randomized group.

Although the active ingredient is the same the medications differ in costs, mode of administration and dosing frequency, which will likely lead to different real world effectiveness and cost-effectiveness. Poor adherence to alcohol medications is well-known, and adherence is the key to improved outcomes; adherence is poorer for medications that need to be taken daily or more than for a monthly medication, particularly for the first month. One might expect efficacy to be the same for these medications if given under ideal circumstances (including the best achievable adherence), but the circumstances are not achievable in real world practice. Research must address this question of relevance.

Both groups receive real world medical management monthly, and all complete 3-month research interviews and contribute utilization data for 1 year. An initial medical management session (20-30 minutes, feasible in hospitalized patients) includes review of the alcohol use disorder diagnosis and alcohol consequences, a recommendation to abstain, medication information, adherence strategies and support group referral. Monthly, in-person follow-up visits (15-25 minutes) address drinking, medication adherence, overall functioning, and group attendance.

We also plan to review medical records (observational cohort) of people with an alcohol use disorder, age 18 or over, and speak English, but who do not enroll in the trial. This would give us the ability to examine all the same healthcare records as those in the trial (current/past and future medical records at BMC, healthcare utilization including addiction treatment, diagnoses, prescription history, health outcomes and behaviors including those related to substance use). As with the trial, we will also review statewide utilization (emergency, hospital and addiction treatment) data from the Center for Health Information and Analysis (CHIA) and the Massachusetts Bureau of Substance Abuse Services. This examination will allow us to compare people who enrolled and received either of the two study treatments to those who also have an alcohol use disorder but did not enroll and receive these treatments. This is an exploratory analysis that will use statistical adjustment for any potential confounders, but our hypothesis is

that people not enrolled in trial will have worse utilization outcomes (more ED visits and hospitalizations and acute substance use care) and worse other outcomes than those enrolled.

2.5. Study Sites

Patients will be recruited from the inpatient services at BMC.

For a previous study, on the inpatient service at BMC we approached 354 admissions per month; 17% of patients had unhealthy alcohol use, and 81% of those enrolled had an alcohol use disorder; 65% of those eligible were enrolled in a year-long study of brief intervention (with some looser and some more stringent entry criteria) at a rate of 3 per week. The health status of patients enrolled will be typical of patients hospitalized on the medical service, ranging from patients with less severe alcohol use disorder (e.g., endorsement of fewer alcohol-related problems and co-morbidities) to those with multiple medical, psychiatric and social ills.

Follow-up visits will occur on the Boston University Medical Campus (BUMC).

2.7. Participant Eligibility

- Alcohol use disorder (based on DSM-5 criteria)
- At least 1 heavy drinking day (at least 5 standard drinks [4 for women] in a day) in the last month prior to hospitalization
- Inpatient on a hospital general medical service
- Adult (age 18 years or greater)
- Ability to speak English (fluency)
- Willingness to name at least 2 contacts for follow-up purposes

2.8. Exclusion Criteria

- Children <18 years of age
- Pregnancy (urine testing if childbearing potential)
- Currently breast feeding
- Urine expanded panel drug test (dipstick) positive for opiates, semi-synthetic or synthetic opioids
- Opioid use (self-report and verification in medical record) in the past 7 days for long acting opioids
- Opioid use in the past 24 hours for short acting opioids
- Discharge prescription for opioids
- Future (next 3 months) need for opioids for an anticipated painful event or surgery
- Known hypersensitivity to NTX
- Acute severe psychiatric illness (currently suicidal or psychotic)
- Cognitive dysfunction that precludes informed consent or RA assessment that participant cannot understand interview questions
- Alanine aminotransferase or aspartate aminotransferase >5 times the upper limit of normal
- Acute hepatitis
- Liver failure
- Known severe thrombocytopenia (<50K)
- Coagulopathy
- Coagulation disorder
- Body habitus that precludes intramuscular injection

- Plans to leave the Boston area in less than one year
- Enrollment in a research study which involves taking a pharmaceutical agent that is expected to interact with naltrexone.

2.9. Recruitment Goals

We plan to enroll 320 adult participants with alcohol use disorder (AUD) and randomize 260 in the ADOPT study (1-3 participants per week for 37 months). On the inpatient service at BMC we previously approached 354 admissions per month; 17% of patients had unhealthy alcohol use, and 81% of those enrolled had an alcohol use disorder; 65% of those eligible were enrolled in a year-long study of brief intervention (with some looser and some more stringent entry criteria) at a rate of 3 per week. There is no upper age limit, though the mean age of those enrolled is anticipated to reflect medical inpatients with AUD at BMC (45 years). Their health status will be typical of patients hospitalized on the medical service, ranging from patients with less severe alcohol use disorder (e.g., endorsement of fewer alcohol-related problems and co-morbidities) to those with multiple medical, psychiatric and social ills. All enrolled in the study will have AUD; some are likely to have co-occurring drug use or dependence (though not opioid use or dependence, as this is an exclusion criterion).

3. Intervention and Control

3.1. Methods Overview

In this trial, human participants are medical inpatients at BMC with an alcohol use disorder and recent heavy drinking. Patients interested in the study, if they agree, will be screened by interview/questionnaire by a research assistant (RA) for eligibility. If the patient is eligible based on initial information available (self-report and existing records), the RA will then seek written informed consent to further assess eligibility (screening laboratory tests, e.g., urine testing, and AST, ALT if not already available). If eligible, he/she will be offered participation in the ADOPT trial. All eligible patients will be informed about the trial and their written consent will be sought. Patients who are ineligible or decline participation will be asked if they would like to participate in the observational cohort and advised to speak with their hospital clinicians regarding treatment options. Those who consent to the trial will be enrolled; they will then complete a baseline research interview, will have blood drawn (if required tests are not available in the medical record), and finally, will be randomized to one of the two study conditions. Blood samples will be stored for future study. This study is a pragmatic (effectiveness, not efficacy) RCT that will compare initiating XR-NTX to PO-NTX at the time of discharge from a medical hospitalization for patients with an alcohol use disorder on: 1) alcohol consumption and consequences, and 2) acute healthcare utilization (including hospital readmission and emergency visits) and cost-effectiveness. 3) In a 3rd, exploratory aim, we will assess moderators of medication effects including demographic, behavioral, and genetic factors. All receive real world medical management monthly, and all complete 3-month research interviews and contribute utilization data for 1 year. Although the active ingredient is the same, the medications differ in costs, mode of administration and dosing frequency, which will likely lead to different real world effectiveness and cost-effectiveness.

3.2. Randomization

After enrollment, informed consent, completion of baseline assessments, and prior to hospital discharge, participants will be randomized to 1 of the 2 study conditions (XR-NTX or PO-NTX). This methodology will minimize bias by assuring allocation concealment at the time of the baseline interview. Randomization will be stratified by 2 key factors: sex and race (Africa America/Black vs not), variables expected to be associated with effectiveness. Stratification will ensure comparable distribution of sex and race across randomized groups. To ensure balance with respect to the number of participants in each group, the permuted blocks strategy (using random block sizes [of 2 and 4]) will be

used. Study staff will use a web-based system for randomization assignments. Study assessment forms will not indicate randomized group.

3.3. INTERVENTION

Participants will receive either monthly XR-NTX (380 mg) or daily PO-NTX (50 mg, up to 100 mg if heavy drinking continues). One month is considered to be 28 days so as to allow follow-up visits to consistently fall on weekdays; however, there is some flexibility to accommodate participants' schedules (see Section 3.4.A. for specified visit windows).

All participants will receive medical management (MM), now available in a generic (i.e., not specific to a study) version adapted for real world medical settings (RW-MM).

3.3.A. XR-NTX

Monthly XR-NTX (380 mg): Participants randomized to XR-NTX will receive one intramuscular gluteal injection (in accordance with the FDA approved label/package insert) of 380 mg of NTX for extended-release injectable suspension at study entry (in the hospital prior to discharge), and 1 and 2 months later (outpatient in BUMC at no charge to the patient), in alternating buttocks. For a missed dose, the participant is encouraged to come as soon as possible to receive the injection, with the subsequent dose a month later. If participants experience adverse effects (such as injection site complications, nausea, and headache), dose reductions of XR-NTX are not possible. This can lead to medication discontinuation or to the use of symptomatic treatments (e.g., acetaminophen).

3.3.A.1. ADMINISTRATION

XR-NTX (trade name Vivitrol) will be prepared and administered by the study nurse. Prior to initiating XR-NTX, an opioid-free duration of a minimum of 7-10 days is recommended for patients, to avoid precipitation of opioid withdrawal that may be severe enough to require hospitalization. The recommended dose of XR-NTX is 380 mg delivered intramuscularly every 4 weeks or once a month. The injection will be administered as an intramuscular (IM) gluteal injection, alternating buttocks for each subsequent injection, using the carton components provided. The needles provided in the carton are customized needles. XR-NTX must not be injected using any other needle. The needle lengths (either 1.5 or 2 inches) may not be adequate in every patient because of body habitus. Body habitus will be assessed prior to administering the screener (part of the exclusion criteria for the trial). For patients with a larger amount of subcutaneous tissue overlying the gluteal muscle, the administering healthcare provider may utilize the supplied 2-inch needle with needle protection device to help ensure that the injectate reaches the intramuscular mass. For very lean patients, the 1.5-inch needle may be appropriate to prevent the needle contacting the periosteum. Either needle may be used for patients with average body habitus. The study nurse will ensure that the XR-NTX injection is given correctly. XR-NTX will not be administered intravenously or subcutaneously. If a patient misses a dose, he/she will be instructed to receive the next dose as soon as possible.

3.3.B. PO-NTX

Daily PO-NTX (50 mg, up to 100 mg if heavy drinking continues): Participants randomized to PO-NTX will receive a bottle containing a 1-month supply (30 days' worth of tablets to allow for 1-2 tablets being lost or having to reschedule an appointment) of oral naltrexone (first one in the hospital at discharge, and then 1 and 2 months later at no charge to the patient, paid for by the study and prepared by the research pharmacist) to be taken once daily (from the patient's perspective, this is how the prescription would be obtained in real clinical practice). The patient will be instructed to take 25 mg a day for 3 days, then to increase to 50 mg a day. If the patient has a prior history of taking PO-NTX and tolerating it well, the study clinicians may start the participant at 50 mg a day from their first dose.

At any RW-MM visit with the study nurse, if the patient continues to have heavy drinking, the dose will be increased to 100 mg daily. Dose reductions and symptomatic therapies (e.g., bismuth subsalicylate) will be considered if side effects occur/persist (nausea, vomiting, abdominal discomfort, fatigue, and headache).

3.3.C. MEDICAL MANAGEMENT

Medical management (MM) itself (without active medication) has efficacy (as much as more specialized therapies) and is cost-effective. Of note, MM addresses patients not taking medication with "Medical Attention" during sessions; the issues below are discussed with the exception of medication. We implement real world medical management (RW-MM) based on the concepts in MM but in contrast, it does not involve extensive training, close adherence to a manual, or the use of the 75 pages of forms and assessments in the MM manual. A nurse will implement RW-MM because these are the most likely clinicians to support AUD pharmacotherapy in hospitals and primary care patient-centered medical homes. The nurse will receive an initial training session from Drs. Saitz and Palfai including content and role play/skills practice to achieve competence. Drs. Saitz and Palfai will be in contact with the nurse regularly and available for additional consultation (mimicking medical setting circumstances). The study nurse will ask about drinking, medication use and side effects, and provide support for abstinence, drinking less, for medication adherence and addressing side effects, and for accessing mutual help groups (e.g., 12-step). Referrals for specialized counseling may be provided if patients request it. Similar to those recommended by NIAAA, standard medical progress notes will be used to support visits, recording vital signs, laboratory results (e.g., liver enzymes), alcohol-related medical conditions, recent alcohol use and consequences, medication taking, side effects, and the clinician's assessment (e.g., drinking/not drinking, consequences/none, taking/not taking medication) and recommendations. Visits are 15-25 minutes and will occur at study entry and then monthly in the primary care clinic (coordinated with routinely scheduled post-hospital discharge medical follow-up visits when possible). If participants cannot come to BMC, including any circumstance when there are restrictions on in-person research activity (e.g. COVID19), these visits can occur in community locations, electronically, over the phone, or over a confidential (HIPAA compliant) video conference. Subsequently medication can be mailed to them upon request (PO-NTX only). The initial visit is slightly longer (feasible in the hospital) because much of the information on diagnosis and treatment is new.

We will audiotape a randomly selected 5% subset of the 1040 possible MM sessions to be able to describe the MM delivered at the end of the study.

3.3.C.1. ADHERENCE DOCUMENTATION

Since this is an effectiveness trial comparing two treatment options, adherence is of specific importance.

Adherence is likely to be associated with outcome and may be the mechanism by which one treatment option is more effective than the other, so we will assess whether the participant takes/receives the assigned medication.

XR-NTX:

- High adherence to XR-NTX will be defined as administration of XR-NTX by the study nurse 3 times. Medium adherence will be defined as 1 or 2 injections.

PO-NTX:

- High adherence will be defined as participant self-report of taking PO-NTX for 90 out of the past 90 days at the 3 Month study visit.
- Medium adherers will be defined by participant self-report of taking PO-NTX for 30-89 out of the past 90 days at the 3 Month study visit.

- We will also assess the presence of detectable 6-β-naltrexol in laboratory assessments (see Section 3.5.B) to confirm reports of recent tablet taking, and will perform a pill count if the participant brings in any unused medication.

We will also assess adherence to study RW-MM visits and exposure to mutual help (e.g., 12-step) groups and specialty alcohol treatment (Form 90). Self-report of outside specialty treatment will also be assessed using statewide utilization databases but self-report is of primary interest because it captures a wider range of services (e.g., medication, mutual help groups, counseling from non-specialists, and employee assistance programs (EAPs)).

3.3.C.2. NON-ADHERENT PARTICIPANTS

There are four potential ways a participant can be considered non-adherent:

1. XR-NTX: fewer than 1 or 2 injections
2. PO-NTX: participant self-report of taking oral naltrexone for 0-29 out of the past 90 days at the 3 Month study visit
3. Not attending medical management meetings
4. Not attending study visits

Participants will not be asked to leave the study for any of these reasons. However, the PI reserves the right to withhold/discontinue medication from a participant if they do not adhere to safety assessments.

If a participant does not attend medical management meetings with the research nurse or does not complete study assessments with the research assistant, they will not receive medication until they complete these visits as clinically appropriate, determined by a study clinician. For example, these visits can be completed electronically, by phone, or by confidential (HIPAA compliant) video conference with the study nurse (as clinically appropriate). When/if they begin to attend visits once again, in-person or remotely (as clinically appropriate), their medication and assessments will resume.

3.3.C.3. MISSED APPOINTMENTS

Baseline Visit

- If a participant is discharged prior to the receipt of their baseline naltrexone dose, the study nurse will arrange for the participant to return within the visit window (see Section 3.4.A) to receive the study drug (injection or 30 days of tablets). The subsequent 1 Month and 2 Month doses will then be administered/dispensed 28 days and 56 days (respectively) after the first dose. The 3 Month follow-up research appointment will be rescheduled for 28 days from the 2 Month visit.
- If a participant does not receive their baseline dose prior to their discharge from the hospital, and is unable to arrange to receive it within the baseline visit window, then naltrexone will not be administered/dispensed until the scheduled 1 Month visit.

1 Month Visit

- If a participant's 1 Month visit occurs after the target visit date, but before the visit window closes, then naltrexone will be administered/dispensed (30 days of tablets) at the time of the visit, and a subsequent dose for the 2 Month visit will be administered/dispensed (30 days of tablets) 28 days later. The 3 Month follow-up research appointment will be rescheduled for 28 days from the 2 Month visit.
- If a participant is unable to come in for the 1 Month visit within the designated visit window, then naltrexone will not be administered/dispensed until the scheduled 2 Month visit.

2 Month Visit

- If a participant's 2 Month visit occurs after the target visit date, but before the visit window closes, then naltrexone will be administered/dispensed (30 days of tablets) at the time of the visit. The 3 Month follow-up research appointment will be rescheduled for 28 days from the 2 Month visit.
- If a participant is unable to come in for the 2 Month visit within the designated visit window, then naltrexone will not be administered/dispensed for this visit and no subsequent doses will be provided.

Should participants in the PO group have tablets remaining at the final study visit, the nurse will determine in his/her medical management assessment whether or not the participant should continue or discontinue the medication. The nurse will discuss this with the participant as part of the transition plan.

3.3.C.4. ABNORMAL LAB VALUES

The study nurse will review all safety lab values (ALT, AST, and GGT). If a participant presents with abnormal lab values, these results will also be reviewed by the principal investigator or a co-investigator. The test(s) should ONLY be repeated if the values are extreme and indicate a serious medical problem. These repeat tests will be performed at the next clinical study visit (i.e., no additional appointments will be made unless directly specified by the study physician).

3.3.C.5. LEFTOVER MEDICATION

When possible, participants will be instructed to bring in all unused study medication at each study visit. The study nurse will conduct a pill-count when applicable, and appropriately review adherence with the participant. A new supply of study medication will be dispensed to the participant to cover them until the next scheduled visit.

3.3.C.6. INTOXICATED PARTICIPANTS

Visits will still take place if a participant has a positive BAC unless, in the judgment of the study staff, the patient is unable to participate due to intoxication. If unable to proceed, then the visit will be considered incomplete and the next visit will be scheduled as per the schedule of visits (at 2 or 3 months). Participants will receive the same dose of study medication; no dose change will be administered to a participant as a result of their intoxication status.

If a participant presents as clinically intoxicated or has a BAC above 0.08, study staff will advise the patient not to drive from the appointment, and discuss and facilitate options to get home such as being escorted/picked-up by family/friends, or study staff will provide assistance with public transportation or taxi or other car service.

If a participant refuses assistance and there is a serious concern of safety, study staff may discuss potential adverse consequences and risks with the participant, and may notify participants that they will contact BUMC security for support.

3.3.C.7. OBSERVED INGESTION

When possible, patients will be reminded not to take their medication on the day of the study appointment, to facilitate observed ingestion (unless it is recommended otherwise by the study physician).

If a patient arrives for the study appointment and has not taken their dose that day, observed ingestion will take place and be documented by the study nurse. If the patient has already taken their medication dose or does not bring in their medication (including on Visit 1, at trial entry), the observed ingestion section of the assessment will be waived for that visit. For future visits, the patient will be advised not to take their medication on the day of the visit before their study appointment.

3.4. Schedule of Data Collection

	Entry<br>Inpatient	Month 1 Visit<br>Outpatient	Month 2 Visit<br>Outpatient	Month 3 Visit<br>Outpatient
**Screening**	*Screener Interview Form A:* RA<br>*Medical Record Review Form B*: RA or Nurse<br>*Clinician Assessment Form C:* Nurse			
**Informed Consent**	RA			
**Research Interview**	*Baseline Interview:* RA			*Follow-Up Interview:* RA
**Urine Tests**	*Pregnancy Test:* RA or Nurse<br>*Urine Tox. Dipstick:* RA or Nurse			
**Blood Tests**	*ALT and AST:* Phlebotomist/Nurse	*ALT and AST:* Phlebotomist/ Nurse<br>*6-β-naltrexol (for PO-NTX participants):* Phlebotomist/ Nurse		*ALT and AST:* Phlebotomist/ Nurse<br>*GGT:* Phlebotomist/ Nurse<br>*dCDT:* Phlebotomist/ Nurse<br>*6-β-naltrexol:* Phlebotomist/ Nurse<br>*PEth:* Phlebotomist/ Nurse<br>*Plasma, serum, and genetics samples:* Phlebotomist/ Nurse
**XR-NTX**	*Injection:* IPS/Nurse	*Injection:* IPS/Nurse	*Injection*: IPS/Nurse	
**PO-NTX**	*30-Day Prescription:* IPS/Nurse	*30-Day Prescription:* IPS/Nurse	*30-Day Prescription:* IPS/Nurse	
**RW-MM**	Nurse	Nurse	Nurse	Nurse
**State Utilization Data**	RA	RA	RA	RA

3.4.A. Visit Windows

Baseline Visit

- Baseline window close: Discharge date + 14 days

1 Month Visit

- 1 Month window open: Date 1st dose dispensed + 28 days
- 1 Month target visit date: Date 1st dose dispensed + 28 days
- 1 Month window close: Date 1st dose dispensed + 55 days

2 Month Visit

- 2 Month window open: Date 1st dose dispensed + 56 days
- 2 Month target visit date: Date 1st dose dispensed + 56 days
- 2 Month window close: Date 1st dose dispensed + 83 days

3 Month Visit

- 3 Month window open: Date 1st dose dispensed + 84 days
- 3 Month target visit date: Date 1st dose dispensed + 84 days
- 3 Month window close: Date 1st dose dispensed + 168 days

3.5. Data Sources

3.5.a. Urine

1. Pregnancy test (qualitative urine β-Human Chorionic Gonadotropin (HCG) in women)
    a. Performed by the research assistant or study nurse at the point of care.
    b. The result of this test will be disclosed to the research participant at the point of care but will not be documented in participants' medical records.
    c. This test may be repeated during the course of the study if a participant of child-bearing potential reports a possible pregnancy which has not yet been otherwise confirmed.
2. Urine dipstick test (for opiates, buprenorphine, methadone and oxycodone) on all patients who are interested and otherwise eligible to participate in this trial at study entry
    a. Performed by the research assistant or study nurse.
    b. The results of this test will not be documented in participants' medical records, but because it informs eligibility, the participant will be made aware if the test led to their ineligibility.

Both tests are done solely for research purposes of determining eligibility.

Any remaining sample will be destroyed after testing.

Sample will not be released to any third parties.

3.5.b. Blood

1. Alanine aminotransferase (ALT) and aspartate aminotransferase (AST)
    a. Routine clinical tests for patients with AUD, if not in medical record, to determine eligibility and to assess potential adverse effects of NTX.
    b. These tests are routine, clinically indicated safety tests for patients taking naltrexone, so these results will be reported in participants' medical records.
2. 6-β-naltrexol
    a. As a measure of adherence, we will collect and store blood samples at 1 Month from the 130 participants assigned to the PO-NTX arm in order to batch and test for quantitative 6-β-naltrexol levels. These samples will be held until study completion, when a select number will be tested at Worldwide Clinical Trials based on participant report of adherence (those that reported non-adherence will not be tested).
    b. Budget permitting, we will also collect and store blood samples from XR-NTX participants at 1 Month and from all participants at the final study visit (3 Months) to batch and test for quantitative 6-β-naltrexol levels. The priority for testing decisions based on budget is 1) PO-NTX participants who report adherence at 1 Month; 2) PO-NTX participants who report non-adherence at 1 Month; 3) all

participants at 3 Months (except PO-NTX participants who report non-adherence); 4) PO-NTX participants who report non-adherence at 3 Months; 5) XR-NTX participants at 1 Month.

c. Samples sent for testing will be labeled with a unique study ID (coded). The lab will never be given access to the mastercode/key, which will be maintained by BUMC investigators.
d. These results will not be reported in participants' medical records.

3. Gamma-glutamyl transferase (GGT)

    a. Performed at the final (3 Month) assessment for all study participants, if not in medical record.
    b. This test is a routine, clinically indicated test for patients who are being treated for AUD, so this result will be reported in participants' medical records.

4. Disialo carbohydrate-deficient transferrin (dCDT)

    a. Collected during the final (3 Month) assessment for all study participants, and tested at the Medical University of South Carolina.
    b. We will batch samples and send them out in several shipments. Samples sent for testing will be labeled with a unique study ID (coded). The lab will never be given access to the mastercode/key, which will be maintained by BUMC investigators.
    c. These results will not be reported in participants' medical records.

5. Phosphatidylethanol (PEth)

    a. Collected at the final (3 Month) assessment for all study participants, and tested at the United States Drug Testing Laboratories, Inc. (USDTL).
    b. We will batch samples and send them out in several shipments. Samples sent for testing will be labeled with a unique study ID (coded). The lab will never be given access to the mastercode/key, which will be maintained by BUMC investigators.
    c. These results will not be reported in participants' medical records.

Blood specimens from all participants (who specifically consent) will be stored for future laboratory and genetic testing; participants will be informed of this and provide written consent (or opt out—opting in is not required for trial participation). This sample will be collected at the 3-month study visit.

3.5.B.1. Detailed Blood Collection Timeline

Entry (5 mL whole blood):

- ALT and AST (if results from within 7 days of screening are not available in medical record)

1 Month (9 mL whole blood):

- ALT and AST (if results from within 7 days of study visit are not available in medical record)
- 6-β-naltrexol

3 Month (25 mL whole blood):

- ALT and AST (if results from within 14 days of study visit are not available in medical record)
- Gamma-glutamyl transferase (GGT) (if results from within 14 days of study visit are not available in medical record)
- 6-β-naltrexol
- Disialo-carbohydrate deficient transferrin (CDT)

- Phosphatidylethanol (PEth)
- Plasma and serum samples to store for future research

Please note, blood collections procedures will be adjusted as needed to protect the safety and well-being of participants when there are restrictions on in-person research activity (e.g. COVID19). We have outlined these modifications below in sections 4.4.B and 4.4.E.

3.6. Observational Cohort

There will be an observational cohort study of those who are not eligible or who do not choose to enroll in the trial but have an alcohol use disorder, are age 18 or over, and speak English. This group will allow us to compare people who enrolled and received either of the two study treatments to those who also have an alcohol use disorder but did not enroll and receive these treatments. Participants will sign an informed consent that will give us the ability to review current and future medical records at BMC to examine all the same healthcare records as those in the trial (healthcare utilization including addiction treatment, diagnoses, prescription history, health outcomes and behaviors including those related to substance use). As with the trial, we will also review statewide utilization (emergency, hospital and addiction treatment) data from the Center for Health Information and Analysis (CHIA) and the Massachusetts Bureau of Substance Abuse Services.

4. Research Assistants (RAs) Procedures

4.1. Introduction

The research assistants will be responsible for informed consent, enrollment of participants, and the research assessment of up to 320 participants at study entry to randomize 260 and follow them for three months at which time they will have a 3-month follow-up visit. They will participate in finalizing study instruments, attend weekly research meetings with the investigators and more frequent staff research meetings, and have extensive training prior to enrolling participants. They will also carry out the follow-up tracking contacts and assessments on an intensive schedule. These latter assessments will require tracking, contacting, and meeting directly with participants. Prior to follow-up interviews, participants will be contacted by the research assistants to confirm appointments and update information. The research assistants will also screen thousands of potential participants, explain the study, answer questions concerning the study, and obtain informed consent.

4.2. Recruitment

4.2.a. Recruitment Process Overview

Identification of potential patient participants

- Medical inpatient at BMC identified with an alcohol use disorder and recent heavy drinking using:
    a. Clinician referral
    b. Clinical Data Warehouse hotlist
    c. RA room to room screening
- RA reviews medical chart for eligibility

↓

Screening process

- Patient screened by the RA for eligibility for the trial with Form A
- If patient eligible based on screener Form A, RA performs medical record review with Form B
- If the patient is eligible based on the self-report and existing records and interested in the study, the RA will seek written informed consent to further assess eligibility with laboratory tests
- If the patient is eligible based on laboratory testing, as assessed by the Research Nurse with Form C, he/she will be offered participation in the ADOPT study
- If the participant is found to be ineligible at any point, he/she will be asked if they are interested in participating in the observational cohort

↓

Enrollment

- RA describes the study
- If patient is interested, RA will obtain informed consent from patient, collect urine sample for toxicology and pregnancy (if necessary) tests, and have blood drawn for ALT/AST tests (if not already in medical record)
- RA will record contact information
- If the participant is available to continue, the RA will administer baseline assessment, and randomize to a treatment group (following baseline assessment)

4.2.B. Preparing to Screen

- Research assistants (RAs) or staff trained to do clinical screening will screen patients who agree in their rooms.
- Study staff will have face to face contact with clinical teams to learn if there are any specific patients that should not be approached, and also to learn of patients who the team wishes to specifically refer, i.e., those most likely to be eligible.
- Records will be reviewed to identify potential participants based on age, absence of current opioid prescriptions, screening laboratory tests, receipt of treatment for alcohol withdrawal or AUD, or other indications that they may not meet eligibility.

4.2.C. Screening

We requested a waiver of documentation of informed consent for screening, to check the medical record for current opioid prescription, recent use or future need, and verification of screening laboratory tests. Additionally, we will confirm self-report of heavy drinking days in the past 30 days and an alcohol use disorder during a face to face visit. Research assistants will advise participants of the purpose of the research screening questions (to identify eligibility for a study), that they may choose not to participate, that their care will not be affected if they choose not to participate, and that there will be no cost, compensation for, or benefit to them of the screening. They will provide their oral informed consent (informed by a review of the elements of consent relevant to the screener). We will record these data anonymously until, and only if, participants sign written informed consent for the ADOPT Study. We will retain anonymous screening data that is collected specifically for ADOPT for participants who do not enroll in the ADOPT trial (i.e., ineligible or eligible but do not consent to participate) so that comparisons can be made related to the representativeness of the sample that does enroll. Written informed consent will be obtained prior to conducting liver enzyme and pregnancy tests and urine test (dipstick) for opioid use for participants for whom these tests are indicated.

Screening will occur in clinical space, typically an inpatient room. Eligible participants will then be informed regarding the study and will provide written consent if they wish to participate in the trial. For those who do not wish to participate in the trial, they will be asked if they would like to participate in the observational cohort.

4.2.C.1. Data Collected During Screening

In order to assess eligibility, we will collect data on recent alcohol use using questions asking about heavy drinking days, and alcohol use disorder (based on DSM-5 criteria assessed using the Alcohol Use Disorder and Associated Disabilities Interview Schedule (AUDADIS)), and liver enzymes (ALT, AST) will be checked either by reviewing the participant's medical record or by blood draw if no results are observed for the prior 7-day period. Women of childbearing age will be tested for pregnancy. Participants who require blood and/or urine tests to determine eligibility will only do so after written informed consent is obtained. The participant's prescription record will also be reviewed. The following additional exclusion items will be assessed to determine eligibility: Pregnancy or breastfeeding, urine expanded panel drug test (dipstick) positive for opiates, semi-synthetic or synthetic opioids/past 7 days exposure to long acting opioids or past 24 hour exposure to short acting opioids, future (next 3 months) need for opioids for anticipated painful event or surgery, discharge prescription for opioids, known hypersensitivity to NTX, acute severe psychiatric illness (currently suicidal or psychotic), cognitive dysfunction that precludes informed consent or RA assessment that participant cannot understand interview questions), acute hepatitis or liver failure, known severe thrombocytopenia (<50K), coagulopathy or coagulation disorder, body habitus that precludes intramuscular injection, plans to leave the Boston area in less than one year, enrollment in a research study which involves taking a pharmaceutical agent that is expected to interact with naltrexone.

4.2.C.1.A. Tests Performed During Screening

- Blood: ALT and AST at study entry (if not already in medical record)
- Urine opioids (dipstick) and pregnancy (for women of childbearing potential) at study entry

Tests are performed to confirm patient eligibility.

4.2.C.1.B. DATA RETAINED DURING SCREENING

All data collected from the ADOPT trial screening assessment will be retained in accordance with the study's protocol. We will retain anonymous screening data that is collected for participants who do not enroll in the ADOPT trial (i.e., ineligible or eligible but do not consent to participate) so that comparisons can be made related to the representativeness of the sample that does enroll.

4.2.C.2. SCREENING OUT

It is possible that some participants will be disqualified after they have provided written informed consent as pregnancy tests (for female participants), liver enzyme tests, and urine tests for opiates, will be conducted post-consent. Such instances could result in exclusion of participants who screen positive for opiates. For example, female participants who screen positive for pregnancy and any participant with ALT and AST levels greater than 5x the upper limit of normal will be excluded. Regarding their data, we retain it in the study database given that they have provided written informed consent.

An observational cohort study will be created from those who are not eligible or who do not choose to enroll in the trial but have an AUD, are age 18 or over, and speak English (see Section 3.6.).

4.3. INFORMED CONSENT

If a patient is eligible and agrees to participate, an RA will discuss the study in detail and obtain the participant's written informed consent. Patients will be given as much time as they need to decide on (and ask questions about) enrollment and provision of consent. RAs will explain all study procedures in language understandable to the study population, will stress the voluntary nature of research, and will provide an opportunity for participants to review the informed consent form (ICF) in full before agreeing to participate. If a patient is illiterate or otherwise unable to read the ICF, the RA will ensure a third party is present to witness the entire informed consent process, and will obtain the witness' signature on the ICF. Patients who choose not to participate in eligibility screening or in the trial will return to care by their inpatient clinicians. At enrollment, participants are specifically asked for permission to contact them should they become incarcerated. Research staff learns that participants are incarcerated by notification from the participant or participant's contact person, or by contacting correctional facilities directly. Incarcerated participants will be presented with an additional informed consent form specifically for incarcerated participants which will give participants the opportunity to accept or decline study participation during incarceration. Incarcerated participants will be informed that participation in the study is not related to decisions about parole. Participants will also be asked during enrollment for their written consent to contact them about future studies for which they may be eligible.

4.3.A. OBSERVATIONAL COHORT

If the patient is ineligible for the trial or is eligible for the trial but explicitly expressed disinterest in participating, the RA will offer participation in the observational cohort. The RA will explain the difference between trial and cohort (there will be no future contact with participant, only record review). If the participant is interested, the RA will continue with the informed consent process (a separate ICF from the trial will be provided).

4.3.B. MEDICAL MANAGEMENT RECORDINGS

When a participant is selected to have their MM session recorded, the RA will explain to the participant that their session was randomly selected to be recorded and will obtain informed consent for doing so. The participant will be informed that recording their MM session is voluntary, and that refusing the recording will not affect their participation in the study. A separate ICF form for these recordings will be provided.

4.4. Interview Protocol

4.4.a. Participant Interactions

After the screening procedures, the RAs will conduct the baseline assessment with the patients who are eligible for the trial. RAs will also administer follow-up assessments at 3 months post-baseline. The study nurse will conduct the RW-MM at study entry, and at 1 and 2 months later.

Please note, although most visits will take place in-person, if a participant cannot come to BMC, including when there are restrictions for in-person research activity (COVID-19), we will conduct necessary visits electronically, by phone or through confidential (HIPAA compliant) video conference as needed and when clinically appropriate for patient safety and well-being.

To expand, we will communicate with participants via phone, BMC Zoom, BU Zoom Meetings for HIPAA, or BU Teams, Epic's patient portal, My Chart, for communication if the subject uses it, and when using BMC email, we will type "secure" in the subject line, which creates a secure messaging system, or for BU email we will use the BU SecureMail email service. We note that since technology changes frequently, it is possible that BMC and BU's secure email systems could change and if they do we will follow BU or BMC IT procedures for such emails. Similarly, if a new secure platform recommended for such communications becomes supported by BU and BMC and/or recommended by the IRB we will use those platforms. One problem that arises with secure email is that it is very difficult for participants to use, particularly the most vulnerable subjects, who have less advanced technology available to them. Thus, if the patient or research subject has agreed, we shall use non-secure communications. In those cases, communication will generally be limited to the minimal detail necessary, and participants will be informed that they should delete messages and take care to keep their information not visible to others on their device. Please note this language is based on BU IRB recommendations, and applies to all other sections describing participant contact, including but not limited to sections 3.3.C and 4.4.C.

4.4.b. Compensation

Participants will be compensated $75 for the initial interview assessment and $75 for the 3 month follow-up interview assessment. There is no compensation for clinical follow-up visits or medication adherence.

In the event there are restrictions on in-person research activity (e.g. COVID19), the blood collection portion of the 3-month study visit may occur at a later date. In this case, additional compensation of $50 will be provided for the extra visit.

4.4.c. Baseline Assessment Procedures

The baseline interview will be conducted immediately following the screening and informed consent process (when possible) or at a time that is acceptable for the patient during the hospitalization. Assessments will be completed before randomization.

Interview: Participants will be assessed as part of this study using well-validated interview instruments covering the following topics:

- Demographics (age, sex, race, education, health insurance, living situation, social supports).

- Alcohol use by Timeline Followback (TLFB) calendar method with a 30-day assessment.
- Drug use by Alcohol, Smoking and Substance Involvement Screening Test (ASSIST) use items.
- Alcohol consequences by the Short Inventory of Problems (SIP-2R).
- Readiness to change alcohol use by brief questionnaire.
- Craving by the Penn Alcohol Craving Scale (PACS).
- Depressive symptoms by the well-validated PHQ-8; it is also sensitive and specific for depressive disorders and responsive to change over time.
- Anxiety by the validated Generalized Anxiety Disorder 7-item (GAD-7) scale.
- Post-traumatic stress disorder (PTSD) by the validated Primary Care PTSD Screen (PC-PTSD).
- Health care utilization. Utilization will be measured primarily by self-report (as was implemented in the COMBINE study of AD pharmacotherapy) because we are interested in any health care received by participants and because they may receive care from many different sources. Self-reported health care utilization is valid. We will use items from the Form 90 Alcohol Intake Revised/Economic Development (AIR/ED) used in COMBINE. They cover mutual-help groups, alcohol- or drug-related nights in hospital, and emergency department and outpatient visits. We supplement these with similarly designed items to capture the range of specialty treatment available locally (e.g., residential treatment, halfway house, medications) as we have in prior studies. Although self-report is primary, we will collect emergency, hospital utilization and medical comorbidity from the BMC Data Warehouse, and for statewide utilization (emergency, hospital and addiction treatment), data from the Center for Health Information and Analysis and the Massachusetts Bureau of Substance Abuse Services. In our prior study, using identifier algorithms, we matched 100% of participants. These data will be used to provide information on the validity of self-report.
- Injury (i.e., any gunshot wound, stab wound, accidents or falls requiring medical attention).
- Health-related quality of life (HRQL) by the EQ-5D (EuroQoL), a questionnaire that describes health status in five dimensions divided into three levels. Its rating scale can be transformed into utilities for use in cost-effectiveness analyses. In addition, we will use questions from the World Health Organization Quality of Life (WHOQOL) assessment and Visual Analog Scale.
- Pain. Participants will be asked to subjectively rate body pain they may be experiencing, indicate the level of interference on daily activities, and to report the use of substances to treat pain/discomfort.
- Objective stress as assessed by the Stressful Life Events questions in the Alcohol Use Disorder and Associated Disabilities Interview Schedule (AUDADIS).
- Perceived stress by the Perceived Stress Scale-4 (PSS4) of the AUDADIS.
- Reasons for drinking by the Drinking Motives Questionnaire (DMQ), validated by Gilson et al. (2013).
- Motivation to quit drinking by the Reasons for Quitting Questionnaire, as modified by Downey et al. (2001).

4.4.C.1. CONTACT INFORMATION

The RA will obtain extensive contact information at study entry (including cell phones, email, Facebook and significant other information). RAs record aliases, names, and contact information of parents, close relatives, employers, friends, shelters, and local treatment programs likely to be able to reach them. Participants will be asked permission to contact these sources if they themselves cannot be reached. The same RA will track a participant over the course of the study, will schedule the follow-up at baseline, and will maintain consistent contact with participants throughout the study (including a research contact in the first month as a reminder for the 3-month follow-up research interview). The follow-up research visit will be preceded by letter, phone, and text reminders to the participant and/or contacts. No shows are pursued by phone and mail (including registered and express) and contact with those listed above. RAs will utilize the hospital electronic scheduling system to identify when a participant is coming in for a clinical visit if a research appointment is missed (RAs will not do this for study clinical visits). If participants cannot come to BMC, assessments can occur in community locations, over the phone, or by confidential (HIPAA compliant) video conference. If incarcerated or hospitalized, interviews are carried out at those locations (approved by the IRB and Department of Health and Human Services (HHS)).

4.4.D. Randomization

See Section 3.2.

4.4.E. Follow-up Assessments

At the final 3 month outpatient visit, a repeat assessment (similar to the baseline) will be completed comprised of demographics, alcohol consumption and consequences, drug use, readiness to change, craving, depression, anxiety, PTSD, healthcare utilization, injury, HRQL, pain, stressful life events, perceived stress, reasons for quitting, and adverse events.

As noted above (4.4.B), in the event of restrictions that limit in-person research visits, we will adjust our procedures for the safety and well-being of participants. The 3-month study visit can be divided into several parts for participant safety. If a participant completes the 3-month research interview and the 3-month medication management remotely, the participant may come in to complete the 3-month blood collection portion of the visit when relevant research restrictions have been lifted. This may occur outside a participant's window.

4.4.E.1. Participant Tracking

The Data Coordinating Center (DCC) will generate reports to assist in participant tracking. An application will be developed that merges information from electronic medical records (appointment scheduling and hospital admission registration) and the research participant tracking system to locate participants in real-time who are either inpatients or have ambulatory clinic appointments at BMC. Two reports will be generated from BMC data: current inpatients and all upcoming outpatient appointments. Data are then compared with the study tracking system database. Potential matches are reported (matched on birth date, social security number, full name, and combinations of these identifiers to allow for errors in the clinical data systems). Research assistants determine correct matches and then contact participants for follow-up. The web-based participant tracking system will create follow-up data collection schedules based on the participant’s enrollment date. The DCC will maintain data management protocols and analytic plans, and will implement a participant tracking system that includes an innovative real-time crosscheck used in prior studies to prompt contact with participants when they appear for care. Because the server is part of the BUMC network, only connections from users authenticated from the domain controller are accepted, thus providing a secure environment for all data.

4.4.E.2. Follow-up With Incarcerated Participants

If a participant is incarcerated during the trial (and will be incarcerated for multiple study visits), we will do one follow-up visit at the 3 month time point. No study medications will be administered and no blood specimens will be drawn for incarcerated participants. This assessment will be similar to the baseline assessment but will be modified as necessary.

At any time, if a participant reports having a legal status, the RA will record the city, county and state where the participant has parole/a warrant/etc. as well as any upcoming court dates. The RA will confirm by calling the court(s) the day after the participant’s court date to see if participant is now incarcerated. If the participant is incarcerated, the RA will inquire about the participant’s “wrap-up” date (when they should be released). The RA will send a jail letter to the participant reminding them of their involvement in the study. If they are incarcerated or if they are especially hard to find/get to come in, the RAs will arrange to interview the participant in jail, and deposit the voucher amount into their canteen. The RAs will check canteen procedures prior to the appointment date, as some jails do not accept money orders in person, or may require cash deposits.

4.4.E.3. Follow Up with Participant In Program

Some programs and holding units (such as TSS and New Hope) do not allow participants to leave for the first weeks or months they are in the program. For these participants, the RAs will find out who their case manager is. The RAs will call the case manager and explain who we are, and that we are trying to arrange a phone interview with the participant. Often programs have payphones for clients to use. If so, check if that participant can use the phone for an extended period of time and arrange an appointment date and time to call the payphone. If the participant has access to a private line, arrange a time and date with the case manager to complete a phone interview. Sometimes, the interview must be broken up into 2 parts, as there are time limits for phone use. This is not preferable, but if it happens, the RA will arrange for 2 periods of time occurring the same day. After the interview, the RA will mail the participant a thank you letter with an enclosed money order.

4.4.E.4. Reluctant Contacts

Many circumstances lead a participant's contacts to be reticent to give out any information regarding the participant. The RAs will be trained to introduce themselves and explain that the participant has an upcoming appointment at BMC and that they would like to leave a message with the contact to remind the participant. If the participant has missed an appointment, the RAs will explain that they are trying to reschedule an appointment at BMC. If asked what department the RAs work in, the RAs will say "Internal Medicine" or "General Internal Medicine".

The RAs will also ask the contact if the participant has a different phone number or address at which they may be better reached.

Another option is to have another RA call that contact in the future as they may be more willing to give the information to a new voice and name. The RA should follow the same procedures as described above.

4.4.E.5. Lost Participants

There are two types of lost participants. The first involve people who suddenly leave a program or fall out of contact with their family, friends, etc. These participants may return to a program where they had previous success, or reach out to their family at any time. Check periodically if this has happened, and mail letters to any shelters they have frequented in the past. The other type of lost participant is the participant who has not been heard from in weeks or months, and whose contacts have not heard from the participant in a very long time. For these participants, the RAs will check once a month with contacts by calling and mailing a letter. The RAs will also mail the participant letters residing in local shelters.

4.5. Participant Retention

Follow-up clinical contacts will be scheduled monthly for clinical assessment and medication refill/administration. Follow-up in-person research interviews will be scheduled at three months post-randomization, coordinated with the final clinic visit. Clinical appointments will be at the Primary Care Center, and research interviews will be there when convenient for participants.

Several strategies will be in place to ensure as complete follow-up as possible. These strategies include appropriate compensation for time and effort of participants and obtaining extensive contact information at study entry (including cell phones, email, Facebook and significant other information) (see section 4.4.C.1 for details regarding contact information).

4.5. Inpatient Medical Service Responsibilities

4.6.a. Interacting with Clinical Staff

Clinical staff interactions are necessary for locating patients. While in the inpatient medical service at BMC, the RAs will maintain a professional demeanor. Since the patient's health care needs always come first, the RAs will integrate themselves into the existing clinical structure.

4.6.b. Using the EMR

The RAs will use the electronic medical record (EMR) system to assess eligibility for the trial during the screening process. The RAs will look to see if there is a positive urine pregnancy test (for female participants), urine toxicity screen, expanded opioid panel results (for buprenorphine, oxycodone, methadone, and fentanyl), ALT, AST, platelet count result <50K/UL, and coagulopathy.

4.7. Office Responsibilities

4.7.a Quality Assurance

The DCC will monitor the quality of the data throughout the study, maintaining vigilance for outliers and other "blips" in precision, and, when found, exert prompt corrective action. The quality control (QC) measures that the DCC will implement include detailed and unambiguous specifications for completion of each of the data collection forms, including rules for coding skipped questions, missing data, refusals, etc.; checks for out-of-range codes and internal inconsistencies; data quality, and participant enrollment and status reports; and interim incremental data reviews that compare data collectors to determine variations among observers in responses to questions on the data forms. Throughout the conduct of the study, the DCC team will be available to the research personnel by email, tele- and web-conference to clarify any questions regarding the data collection.

5. Investigational Pharmacy Services (IPS)

IPS will be responsible for ordering, receiving, storing, dispensing and destroying all investigational products. XR-NTX will be provided by Alkermes. PO-NTX will be purchased locally by the site.

Investigational Medication Prescribing

Once a participant is enrolled and randomized into the study, a prescription will be written by one of the studies' authorized prescribers and delivered to the IPS. The following information will be provided to the research pharmacy in order for the study medication to be dispensed:

1) A copy of the current, IRB-approved ICF signature page
2) The randomized treatment assignment for the participant
3) The prescription order signed by an authorized prescriber
4) If the participant is to start study drug or is being discharged outside the normal IPS business hours, study staff will arrange with IPS to have the study drug available in advance
5) The follow-up visit schedules (this will be need weekly or every other week)

Once the study medication is ready to be dispensed, the research pharmacy can deliver the medication to the nursing unit where the participant is located or the study nurse can pick them up.

Investigational Drug Accountability Recording

The receipt and dispensing of study drugs will be documented in accordance with the IPS' SOP Accountability Records for Investigational Drugs.

Dispensing of Study Medications for Inpatients

The IPS will dispense the appropriate amount of the study drug to the nursing unit where the participant is located. The IPS will label the study medication with the participant name, medical record number, study drug name, drug strength, administration instructions, PI name, protocol name, and “For Investigational Use Only” labeling.

The study medication will be loaded in the Pyxis machine on the medical inpatient service in a designated pocket/location.

Participants randomized to PO-NTX will receive a study prescription for a 1-month supply of oral NTX to be taken once daily. The participant will be instructed to take 25 mg a day for 3 days, then to increase to 50 mg a day. If the participant continues to have heavy drinking, an authorized prescriber may increase the dose to 100 mg daily. The clinical staff will communicate to the pharmacist that the dose should be increased and the pharmacist will adjust the supply accordingly.

Participants randomized to XR-NTX will receive the IM dose of NTX to be administered monthly.

Investigational Drug Storage

Study drug will be stored in accordance with the manufacturer’s labeled recommendations.

Investigational Drug Disposal

The BMC research pharmacy is responsible for proper disposal of used and unused investigational drugs according to regulatory and protocol requirements outlined by the study protocol.

- All participant-returned study drugs will be handled by the research team. The IPS may assist in the disposal of the study drug upon request.
- Any drug that is damaged or is exposed to a storage temperature excursion will be quarantined and maintained by IPS until final disposition instructions are received.
- Any drug supplies that have expired will be quarantined and maintained by IPS until final disposition instructions are received.
- Any unused drug at the close of the study will be maintained by IPS until final disposition instructions are received.

Once all drug accountability and participant returns have been properly reconciled and documented, the PI will give permission to either return investigational products to the supplier or dispose of on site.

All investigational drugs will be discarded in the pharmaceutical waste containers per Environmental Health and Safety guidelines and BMC policies.

5.1. XR-NTX

The trade name of the injectable drug is Vivitrol and the generic name is naltrexone. The drug will be supplied at no cost and will be dispensed at the investigational pharmacy. It will be administered monthly via intramuscular gluteal injection. The BMC research pharmacist/Investigational Pharmacy Service will dispense for each visit. The study nurse will prepare the injection just prior to administering.

Participants (130) randomized to the XR-NTX group will receive a 380 mg dose of XR-NTX after randomization (and before being discharged from the hospital). They will receive a second 380 mg dose at month 1 and a third and last 380 mg dose at month 2. All doses will be administered by a study nurse.

5.2. PO-NTX

The generic drug name is naltrexone hydrochloride tablet. The drug will be supplied at no cost and will be dispensed at the investigational pharmacy. It is taken once daily, orally. The BMC research pharmacist/Investigational Pharmacy Service will prepare medication to dispense at each visit.

Naltrexone hydrochloride tablets USP 50 mg is indicated in the treatment of AUD and for the blockade of the effects of exogenously administered opioids.

After randomization (and before being discharged from the hospital), participants randomized to the PO-NTX group will be instructed to take 25 mg a day for 3 days, then to increase to 50 mg a day.

- Participants in the PO-NTX group will receive a bottle containing 30 50 mg tablets at discharge (baseline) and at the 1 and 2 month visits. Participants will be instructed to take ½ tablet (25 mg) for the first 3 days at baseline, then 1 tablet (50 mg) daily.
- If the participant has a prior history of taking PO-NTX and tolerating it well, an authorized prescriber may start the participant at 50 mg a day from their first dose.

At any time during the study period, an authorized prescriber may increase the participants' oral naltrexone dose if heavy drinking continues and the participant is able to tolerate the 50 mg dose. The clinical staff will communicate to the pharmacist that the dose should be increased and by how much (up to a total of 100 mg) and the pharmacist will adjust the dose accordingly.

At any time during the study period, an authorized prescriber may reduce the participants' oral naltrexone dose if the participant reports any adverse effects typical of too high a naltrexone dose. The clinical staff will communicate to the pharmacist that the dose should be reduced and the pharmacist will adjust the monthly tablet distribution accordingly.

If a participant reports losing their medication during the study period, they will be encouraged to come in as soon as possible to receive a replacement for the remainder of their tablets for the month prior to their next study visit. If participants cannot come to BMC, including reasons related to restrictions on in-person research activity (e.g. COVID19), medication can be mailed to the participant (PO-NTX only).

6. Assessments: Measurements and Data Collection Process

6.1. Baseline Assessment

The RAs will administer an electronic version of the baseline assessment for participants who are eligible for the trial.

6.2. Follow-up Assessment

At 3 months post-baseline, the RAs will administer an electronic version of the follow-up assessment for participants who are enrolled in the trial.

6.3. Quality Assurance

Assessment forms will be reviewed to establish that they were completed correctly by the RAs and proper training will be provided prior to the start of enrollment for the study.

To assure confidentiality, each participant will receive a unique identification number and research data collection and data entry forms will be labeled only with this number, and will contain no other individual identification. Only the written informed consent forms, participant locator information, and a master list of participants and participant

study identification numbers will have the participant number and identifying information on them. There will be only one master list of names and identification numbers. These data will be kept in a secure electronic environment accessible to the principal investigator, the project manager and data managers. Tracking information will be kept similarly (accessible to research assistants). During tracking, any contacts will be told that participants are being contacted to follow-up in a health study; the nature of the study will not be disclosed.

Computer data will be password protected, and accessible only to research assistants needing the information for follow-up purposes. A Certificate of Confidentiality has been awarded by the National Institute on Alcohol Abuse and Alcoholism, and will be effective prior to enrollment, to protect from release of data even under court order or subpoena.

7. Participant Safety

7.1. Protocol Review and Study Monitoring

The study PI and project manager will be responsible for monitoring protocols for the trial, research assistants and data entry staff will be asked to monitor their own performance and report any issues that arise to supervisors. If issues are noted from any source, it will be corrected through clarification, additional training, or changes to systems as necessary.

Additionally, in the event of restrictions on in-person research (e.g. COVID19), we will adjust our procedures as needed to maintain participant safety and well-being.

7.3. Potential Risks

Potential risks for participants in the ADOPT study include psychological stress from the research interviews or from recognizing health disorders during the interviews or tests, loss of confidentiality, phlebotomy associated risks (including risks to staff associated with exposure to biohazards), and medication risks. It should be noted that alcohol withdrawal is not a risk of this study because patients are not discharged from the medical inpatient service if they are experiencing withdrawal, and patients do not have access to alcohol during hospitalization (thus they have already been abstinent during hospitalization).

7.3.a. Psychological stress

To minimize this risk, we will select participants who understand the study and are willing to participate. The PI and study physicians are available to respond to participant concerns with referrals at the study site (a full service medical center including immediate emergency psychiatric care) as needed should the participant report new psychological distress as a result of the interview.

Laboratory tests could reveal disorders that require clinical follow-up. The tests done solely for research purposes will not be shared with participants as they have no clinical relevance and will not be done in real time (i.e., 6-β-naltrexol). Other tests routinely done in the course of clinical care (i.e., ALT, AST, and GGT) can be shared with patients and will be available to their clinicians in their electronic medical record. It is a risk that patients and clinicians will act on these results, but decisions will be in the hands of patients and their clinicians, with consultation by investigators if they desire. Of note, a woman could find out she is pregnant due to urine pregnancy testing for the study. This could be a surprise and therefore psychologically stressful. Research staff will be trained to deliver the results with assistance by investigators, and patients will be immediately connected with clinical care as appropriate (psychological, and for any pregnancy related and medical issues).

7.3.b. Loss of Confidentiality

Loss of confidentiality by someone seeing the responses to interview assessments is potentially the most serious risk of the proposed study, though it is very unlikely because specific procedures will be implemented to prevent such disclosure. In addition, much of the most sensitive information (e.g., AUD diagnosis) is already in medical records and the additional increase in risk of recording it for research purposes, particularly given the protections in place for such records that is far greater than clinical records, is small. There is a risk that participants may experience loss of confidentiality when research assistants make attempts to contact them for follow-up. All participants will be informed and provide their consent for the study to reach them via contacts they provide and via BMC real-time utilization records. When research assistants attempt to make any contact, all communications will be identified as coming from a "Boston University health study," not an alcohol study. In the event study assessments are conducted via phone or confidential video conference, study staff will advise participants to take precautions to minimize the risk of a breach of confidentiality.

We have been awarded an NIH Certificate of Confidentiality to further protect against loss of confidentiality and have that protection in place prior to enrolling any participants. Suicidal ideation is an exception to confidentiality in research and requires clinical intervention. If a participant reports that he/she intends to hurt his/herself, RAs are required by law to report that. Since RAs are not trained to deal with mental health crises, the RA should refer to a study-specific protocol to determine the next steps to take in order to address this situation if it ever presents itself. Typically this includes contacting the study physician and the Project Manager. Another exception to confidentiality in research is homicidal ideation. Acute homicidal ideation is defined as thoughts or plans of harm to others. Expressions of homicidal ideation can occur at any point, therefore the RA should remain vigilant and aware throughout the research interview. Like suicidal ideation, homicidal ideation requires clinical intervention.

7.3.c. Phlebotomy Associated Risks

An additional risk is that of phlebotomy: bruising, bleeding, infection, phlebitis, and pain. Bruising is common, pain with needle stick is universal, the other risks are rare. Using experienced phlebotomists will minimize these risks. We also minimize risks by using results from tests in medical records already done for clinical purposes when they can suffice for the research.

7.3.d. Biohazard Containment

An additional risk is that of biohazards, as the transmission of blood-borne pathogens can occur through contact with contaminated needles, blood, and blood products. Using experienced phlebotomists will minimize these risks, as will having trained personnel ship and handle specimens for this study.

7.3.e. Psychological Stress from Medication

This is a minimal risk, is not likely, and will be minimized further by the methods described above (specifically, by selecting participants who understand the study and who are willing to participate). Participation is voluntary and participants can withdraw at any time.

7.3.f. Genetic Risks

The genetic testing proposed is neither diagnostic nor prognostic of a disease. It is testing that may be associated with treatment response to naltrexone. The results will not be shared with participants. Therefore it is difficult to imagine socioeconomic risks or psychological risks even if confidentiality were to be breached or the participants informed inadvertently (the latter of which is essentially impossible). If known, a participant might believe that they would or would not respond to naltrexone. If questions arise from participants the PI will address them. A genetic counseling service for this type of testing would not be relevant and is not provided.

7.3.G. Medication Risks

Participants in the trial will be randomly assigned to receive either naltrexone tablets or extended-release injectable naltrexone. Recommended dosages will be used (25-50-100 mg PO-NTX; 380 mg XR-NTX). Participants will be informed about the potential adverse effects of the medication. Proper participant selection will minimize potential risks.

7.3.G.1. Hepatocellular Injury

The most serious risk of naltrexone (both PO and XR) is the potential for hepatocellular injury when taken in excessive doses (the margin of separation between the apparently safe dose of naltrexone and the dose causing hepatic injury may be only five-fold or less).[1] The dosages of PO- and XR-NTX to be used in this study (25-50-100 mg and 380 mg, respectively) do not appear to be hepatotoxins. Liver toxicity has been seen in patients taking over 300 mg orally daily. In AUD trials, liver enzymes that are initially elevated (likely due to excessive alcohol use) decrease in participants taking naltrexone.[2] Participants will be instructed to take no more than 100 mg per day of PO-NTX. XR-NTX will be administered intramuscularly once monthly by a nurse, at a dosage level found to be safe and effective. Exposure to naltrexone can precipitate severe withdrawal if the participant is dependent on opioids. Participants will be screened for recent opioid use and dependence, and these participants will be excluded. Additional serious (potential) risks of XR-NTX include infection or sterile abscess at the injection site, with induration, cellulitis, hematoma, abscess, sterile abscess, and necrosis. Eosinophilic pneumonia was reported in 2 cases (one was confirmed) in people taking XR-NTX. It is not clear if it was related to the medication. Depression-related events (adverse events 10% vs. 5% with placebo) have been more common on XR-NTX than placebo; 1% vs. 0% respectively had events leading to treatment discontinuation and 1% vs. 0% had suicidal thoughts and behaviors.

7.3.G.2. Opioid Blockage Risks

Since naltrexone blocks opioid receptors, it can be associated with three consequences related to that blockade. First, if a patient requires opioid analgesia it will be difficult to achieve and require high doses in a monitored setting and/or other methods of analgesia. Second, if a patient chooses to try to override the opioid blockade with high doses of illicit opioid they risk potentially fatal overdose. Lastly, after a course of naltrexone treatment, a patient with prior opioid dependence may be more sensitive to opioids, which, again, if used illicitly, could lead to overdose.

7.3.G.3. Other Common Adverse Effects

Other common adverse effects of both PO-NTX and XR-NTX (>5%) versus placebo, respectively, include nausea (33% vs. 11%), vomiting (14% vs. 6%), decreased appetite (14% vs. 3%), headache (25% vs. 18%), dizziness (13% vs. 4%), asthenia (e.g., malaise and fatigue, 23% vs. 12%), anxiety (12% vs. 8%) and depressive symptoms (8% vs. 4%). Additional risks of XR-NTX are injection site reaction (including pain, tenderness, induration, swelling, erythema, bruising or pruritis), joint pain/stiffness (12% vs. 5%), and muscle aches or cramps (8% vs. 1%). These effects are generally mild and are responsive to dose reductions and/or symptomatic therapies. Any injection site reaction (vs. placebo) occurred in 69% vs. 50% but pruritis occurred in 10% vs. 0%, nodules/swelling 15% vs. 4%, pain 17% vs. 7%, induration 35% vs. 8%. Of note, in a large multi-site open-label study of oral naltrexone for alcohol use disorder, the most common new-onset adverse clinical events in the naltrexone group were nausea (9.8%) and headache (6.6%).

---

[1] Moyer VA. Screening and behavioral counseling interventions in primary care to reduce alcohol misuse: U.S. Preventative Services Task Force Recommendation Statement. Ann Intern Med. Published online 14 May 2013. doi: 10.7326/0003-4819-159-3-201308060-00652

[2] Chen CM, Yi H, Falk DE, Stinson FS, Dawson DA, Grant BF. Alcohol use and alcohol use disorders in the United States: Main findings from the 2001-2002 National Epidemiologic Survey on Alcohol and Related Conditions (NESARC). Alcohol Epidemiologic Data Reference Manual. 2006;8(1).

Naltrexone was discontinued in 15.0% of patients because of adverse events, most frequently nausea. The results of liver enzyme tests in the naltrexone group were similar to those in the reference group.

7.4. Risk Assessment

The risks above minimal risk are breach of confidentiality and risk of a serious adverse event associated with naltrexone. The risk of breach of confidentiality is best addressed by appropriate study procedures; however, the PI will be responsible for assuring that study procedures are adhered to regarding data security, transfer, and communications in tracking participants by meeting regularly with study staff, reviewing procedures and assuring quality control reviews of study forms. Serious risks from naltrexone use are possible, but uncommon, and include injection site complications and hepatic injury. These risks are not a result of experimental use of the medication, rather from use of the medication as indicated. These risks are addressed in more detail above. The risk of adverse effects of the study drug is best addressed by appropriate study procedures. The PI will be responsible for assuring that study procedures are adhered to regarding monitoring, managing, and reporting adverse effects of the study drug by meeting regularly with study staff (especially the study nurse), reviewing procedures and liver enzyme results, and assuring quality control reviews of study forms.

7.5. Adverse Event Reporting and Discontinuation of Study Treatment

If a research team member identifies or suspects an adverse event has occurred, he/she will notify the project manager and PI. Adverse events and deviations will be reported to the BUMC IRB as necessary.

Adverse events will be tracked using the comment section of the assessments and documented in the ADOPT Adverse Events Log. The research team expects a low volume of events.

7.5.a. Adverse Events

Adverse Event: any untoward or unfavorable medical occurrence in a human participant, including any abnormal sign, symptom, or disease, temporarily associated with the participant's participation in the research, whether or not considered related to the participant's participation in the research. Examples of adverse events include:

- Breach of confidentiality – participant data being shared outside of the study (or lost or stolen)
- Participant health problem occurring during the course of the study (participant death needs to be reported)
- Participant or family complaints regarding recruitment, confidentiality concerns, or conduct of the study
- Expressions of fear or emotional upset

7.5.a.1. Serious Adverse Events

Serious adverse events include any event that results in any of the following events:

- Death
- A life-threatening adverse event
- Inpatient hospitalization or prolongation of existing hospitalization
- A persistent or significant disability/incapacity
- A congenital anomaly/birth defect
- An event that requires medical or surgical intervention to prevent one of the outcomes above

7.5.A.2. UNANTICIPATED PROBLEMS

Unanticipated problems are defined as events, experiences or outcomes that occur any time during or after the research study that meet all of the following criteria:

1. Is unexpected (in terms of nature, severity, or frequency) given (a) the research procedures that are described in the protocol-related documents (such as the IRB-approved research protocol and informed consent document); and (b) the characteristics of the participant population being studied; AND
2. Is related or possibly related to participation in the research (in this guidance document, possibly related means there is a reasonable possibility that the incident, experience, or outcome may have been caused by the procedures involved in the research); AND
3. Suggests that the research places participants or others at a greater risk of harm (including physical, psychological, economic, or social harm) than was previously known or recognized OR are by definition SERIOUS.
   (Note: all incidents, experiences or outcomes that are unexpected, AND related or possibly related AND are SERIOUS are unanticipated problems.)

To be considered an unanticipated problem in this study, the occurrence must be 1) unanticipated, 2) related to the research, and 3) a new risk or serious (e.g., death, life threatening, hospitalization, disability, birth defect, or requires medical or surgical intervention to prevent any of those from happening).

7.5.A.2.A. RELATEDNESS

**Related**	**Unrelated**
Possibly, Probably, Definitely	Unlikely, not related
There is at least a reasonable possibility that the incident, experience, or outcome may have been caused by the procedures involved in the research	There is not a reasonable possibility that the incident, experience, or outcome may have been caused by the procedures involved in the research

7.5.A.2.B. EXPECTEDNESS

**Unexpected**	**Expected**
An adverse event or suspected adverse reaction is considered “unexpected” if it is not consistent with the risk information described in this protocol. “Unexpected,” as used in this definition, also refers to adverse events or suspected adverse reactions that are mentioned in this protocol as occurring with a class of drugs or as anticipated from the pharmacological properties of the drug, but are	The event is anticipated in nature, severity and frequency as listed and described in this protocol.

not specifically mentioned as occurring with the particular drug under investigation.	

7.5.A.2.C. ASSESSING AN INCIDENT

Medical vs. Not

- Adverse events must be medical occurrences; other types of events could potentially be unanticipated problems

Expectedness

- Is event expected? Does it differ in severity, frequency, or duration from what was expected?

Relatedness

- Is event related to protocol?
    - Unrelated
        - Not related
        - Unlikely related
    - Related
        - Possibly
        - Probably
        - Definitely

Severity/change in risk

- For all events: Does this incident suggest that the research places participants or others at a greater risk of harm than was previously known or recognized?
- Non-serious adverse events (medical) only: Assign severity grade.
    - Grade 1: Mild; asymptomatic or mild symptoms; clinical or diagnostic observations only; intervention not indicated.
    - Grade 2: Moderate; minimal, local or noninvasive intervention indicated; limiting age-appropriate instrumental ADL (preparing meals, shopping for groceries or clothes, using the telephone, managing money, etc.).
    - Grade 3: Severe or medically significant but not immediately life-threatening; hospitalization or prolongation of hospitalization indicated; disabling; limiting self-care ADL (bathing, dressing and undressing, feeding self, using the toilet, taking medications, and not bedridden).
    - Grade 4: Life-threatening consequences; urgent intervention indicated.
    - Grade 5: Death related to AE.

7.5.A.3. PROTOCOL DEVIATIONS

Protocol deviations are changes that are made to the defined IRB approved protocol either accidentally or intentionally. Examples include:

- Enrolling an ineligible patient
- Switching a participant's randomization group
- Changing the content of the intervention
- Asking additional questions during the assessment

7.5.B. Recording of Unanticipated Problems and Adverse Events

The investigator will use active surveillance to seek information on adverse events. Any adverse events related to study procedures (including the research interview, blood draws, or the study drug) will be monitored using the ADOPT Adverse Events Log and reviewed at meetings of the study team.

Study staff report potential events when they occur and they are reviewed in real time and during weekly meetings, where the statistician, project manager and co-investigators assist with review.

The study will take place in a medical setting where standard procedures are in place to assist patients who experience acute events. If consequences arise due to research procedures (e.g., distress, anxiety, or suicidal thoughts) the physician will be available to assess participants and make appropriate interventions or referrals based on the clinical circumstances.

The PI and study physicians are available to respond to participant concerns with referrals as needed should the participant report new psychological distress as a result of the interview.

If an incarcerated participant becomes distressed during the interview, the research team will notify the Director of Social Services or other appropriate person at the facility. All other risks/discomforts remain the same.

7.5.C. Reporting of Unanticipated Problems to the IRB

We will follow guidelines set forth by the BUMC Institutional Review Board (IRB) regarding "unanticipated problems."

Unanticipated problems must be reported to the IRB <u>within 2 days of the investigator learning of the incident</u>. The report will explain why the incident is considered an unanticipated problem and how the protocol will be modified.

Incidents that do not meet all three of the criteria for "unanticipated problems" noted above will be considered adverse events or serious adverse events but not unanticipated problems. In these cases, they will be reported to the IRB at the time of the annual progress report. The report will provide information on cumulative incidents, and if in total the events suggest that participants or others are at greater risk, then the investigators will address whether or not the consent form needs to be changed.

7.5.D. Reporting of Adverse Events to the IRB

We will follow guidelines set forth by the BUMC IRB regarding "adverse events."

Any adverse events related to study procedures will be monitored by the study using a study adverse event form and reviewed at meetings of the study team. Hospitalization will be assessed at follow-up. This is an expected adverse event because of the nature of the population under study. As a result, while they will be reported in accordance with regulations as the study staff becomes aware of them during research interviews, the study will not usually have access

to reasons for hospitalization and causality, and will therefore not compare group rates until the end of the study when analyses of these outcomes are done.

Deaths and other serious adverse events reported to research staff will be reviewed by the PI and handled similarly. These events will be reported on a study adverse event form and reviewed by the project manager and PI as they occur.

The PI will review forms for new SAEs within 2 days of study staff learning of the event. If a new SAE is unresolved at the time we learn about the event, study staff will monitor the participant/event and complete a new report form (a "follow-up" SAE report) with any updated information if the status of the unresolved SAE changes, and the PI will review these follow-up forms in a timely manner. If the change in status of an unresolved SAE alters the original determination of the event's risk/relatedness, the PI will review the follow-up report form and report the event to NIAAA within 2 days. All new and follow-up reports will be included in annual AE summary reports to NIAAA and the IRB.

7.5.E. Blinding

There is no blinding in the ADOPT study.

7.6. Stopping Rules

Reasons we may need to discontinue naltrexone for individual participants include participants who become pregnant, start breastfeeding, or become incarcerated during the trial. Additionally, an increase in liver enzymes (assessed at the 1 month visit) will prompt an assessment to determine the relation to study medication. If it is related and substantial (e.g., a 3-5x increase in ALT or AST over last recorded levels, or ALT or AST levels in a range >5x the upper limit of normal), the medication will be discontinued. The study nurse will communicate this to the research pharmacist. If opioid analgesia is needed, this needs to be provided in a monitored setting. Participants will be informed of the risks of illicit opioid use and sensitivity to opioids after a course of treatment of naltrexone, and advised to consult a physician and disclose their naltrexone use should opioids become necessary. To assist with this issue, participants will also be given and advised to carry a wallet card with information about naltrexone to alert medical personnel in the event of an emergency. For less serious but more common adverse effects, such as injection site complications, nausea, headache, and fatigue, symptomatic therapies will be considered (e.g., bismuth subsalicylate, acetaminophen). Dosage reductions are not possible for XR-NTX, though the clinical team will assess whether to discontinue the study medication (i.e., forgo future monthly injections). Dose reductions of PO-NTX will be considered if side effects persist. All of these reports will be monitored by the PI and study team regularly to identify any serious trends and to assure procedures to minimize risk are appropriate.

Participants who discontinue naltrexone will continue to be followed for medical management and research visits.

8. Data Management

8.1. Data Collection

Data will be collected by interview via web-based data capture system on Surface tablets. The DCC will design, develop and maintain the electronic data collection forms, participant and data tracking, and underlying SQL database systems, and implement procedures for data quality control, including multiple checks for entered data. Electronic data collection forms will be designed to read easily, have clear instructions, preprogrammed skip patterns, real-time range checks and internal logic to minimize missing data, resulting in "cleaner" data at capture. The website and accompanying database will be located on secure, password-protected servers, behind the Boston University firewalls. The web and database servers use Secure Socket Layering (SSL) to ensure data security and confidentiality. Servers

incorporate RAID hard drives for data redundancy. A separate web server dedicated for Cold Fusion applications is also available.

The DCC will support the Boston University research team in designing, piloting, and implementing data collection forms by ensuring that the data fields are unambiguous, and the systems for recording information function smoothly. All forms will be designed to read easily, have clear instructions, and have pre-coded responses.

8.2. Quality Control Process

The Data Coordinating Center (DCC) will assure high quality forms, monitor data quality, and track and link the multiple data sources. Data will be linked and entered using multiple checks. The DCC will develop data collection forms, design the database management system for data entered and for participant tracking, implement procedures for quality control, and provide statistical programming and collaborate in-report writing and presentation of study results.

The quality control measures that the DCC shall implement and describe in the MOP include detailed and unambiguous specifications for completion of each of the data collection forms, including rules for coding skipped questions, missing data, refusals, etc., and interim incremental data reviews that compare data collectors to determine variations among observers in responses to questions on the data forms. Throughout the conduct of the study, the DCC team will be available to the research personnel by email, tele- and web-conference to clarify any questions regarding the data collection.

The DCC will monitor the quality of the data throughout the study, maintaining vigilance for outliers and other "blips" in precision, and, when found, exert prompt corrective action. Quality control measures will be: detailed and unambiguous specifications for completion of study forms, including rules for coding skipped questions, missing data, and refusals; checks for out-of-range codes and internal inconsistencies; data quality, and participant enrollment and status.

8.5. Data Security and Confidentiality

See Sections 6.3 and 8.2.

8.5.a. Coded Data

A unique ID will be used to identify individual records, and all data and samples will be labeled only with this study ID number (i.e., not the participant's name, date of birth, medical record number, etc.). The unique study ID will be linked to participant identifiers via a mastercode/key. Restricted access to participant contact information and the mastercode/key will be limited to members of the research staff as necessary to complete their duties. Locator/contact information and a master enrollment list will include identifiers, but participant names will not be entered into the analytical database. The mastercode/key that links study data to identifiers will be stored separately from the study data (i.e., in separate physical files and separate databases) and protected as described below.

8.5.b. SSL Encryption

Eligibility for the ADOPT Study will be verified by the web data capture application. The underlying scheme, developed by the DCC, relies on live access to data elements necessary for randomization stratification. Allocation is concealed from study staff and participants until the assignment is made to avoid interviewer bias. The web-based participant tracking system will create follow-up data collection schedules based on the participant's enrollment date. The DCC will maintain data management protocols and analytic plans, and will implement a participant tracking system that includes an innovative real-time crosscheck used in prior studies to prompt contact with participants when they

appear for care. Because the server is part of the BUMC network, only connections from users authenticated from the domain controller are accepted, thus providing a secure environment for all data. Specifically, the policies for computer systems security implemented at BUMC are as follows:

Provide physical security of data. The server resides in the same building as the Boston University Medical Center Office of Information Technology (OIT) servers. The lobby of the building in which the systems reside is under the security purview of the Boston University General Services Security Office and is under surveillance. All central systems are physically secured behind two card-access doors with access to the primary door restricted to key personnel in the OIT. Access through the primary door is also protected by a keypad alarm system that is tied directly into the on-site central emergency response security control center. Written policies exist for contingencies to provide access to the room to those not explicitly authorized. Provide virtual security via connectivity. Internal access to all systems is done via MicroSoft Challenge Handshake Authentication Protocol. With the exception of internet provider-based services, external client access must first gain access to the internal network before connecting to the systems. This connection is initiated via a Virtual Private Network connection using Point-to-Point Tunneling Protocol or through the University's modem pool which require Kerberos authentication. All web-based mail is encrypted with high-encryption domestic SSL.

8.6. Web Systems

The ADOPT project uses three web systems: a screening system, a tracking system, and Research Electronic Data Capture (REDCap) projects for research assessments and adverse events. The screening system collects eligibility information in a de-identified manner through trial enrollment, observational cohort enrollment, or ineligibility or refusal to participate. Subsequent to participant consent for either trial or observational cohort, the tracking system is used to enter participant contact information, schedule visits, track that all visit components were completed, and enter non-assessment contact logs. The REDCap system is used for participant randomization and collection of assessment data and adverse events.

Study staff will be provided with usernames and passwords for each of the websites. Each web system has a timeout built in.

8.6.A. Screening System

The screening system is used to collect and document eligibility for the trial and observational cohort, including consent dates and post-consent eligibility lab checks.

The screening website does not include personal identifiers, but includes an automatically-created numeric screening ID and a random word chosen by RAs which will be used to help identify each patient during the screening process.

The three screening forms (A, B, and C) will be used to determine eligibility for the trial and observational cohort, and the appropriate enrollment form will display based on the data collected in those three screening forms. Participants who are ineligible for the trial or refuse to participate in the trial will still be offered enrollment in the observational cohort, provided they are eligible (fluent in English, 18+ years old, with AUD as assessed using the AUDADIS). The website includes reports to help research staff easily access forms during screening and to track form completion during the screening process.

8.6.B. Tracking System

Once consent for the trial or observational cohort is obtained, protected health information and contact information may be entered into the tracking system. The tracking system is primarily used to schedule visits and track non-assessment contacts with each participant. The system includes a contact log and tracking forms to confirm all components of each visit are completed.

Although the screening ID will be entered into the tracking system so that screening records can be linked to each participant, the primary ID in the tracking system is the Study ID which is automatically assigned when a participant's information is first entered into the tracking system.

8.6.C. REDCap System

The research assessments and relevant clinical data (i.e., vitals, BAC) will be entered into the REDCap data collection system, as will any adverse events. The REDCap system also includes the randomization module that will be used to randomize trial participants to the oral medication (daily PO-NTX: 50 mg, up to 100 mg if heavy drinking continues) or injection arm (monthly XR-NTX: 380 mg).

The REDCap system will only be used for trial subjects, not observational cohort participants. It will rely on the Study ID automatically created in the tracking system as the unique participant identifier.

9. Statistical Analysis

Sections 9.1-9.3 are superseded by Appendix 1.0 ("Statistical Analysis Plan"). The Statistical Analysis Plan includes updates, further detail, and technical specifications as it relates to the primary analysis.

9.1. Outcomes

The primary outcome of the study (Aim #1) is percent heavy drinking days (%HDDs) over the past 30 days assessed at 3 month follow-up by the Timeline Follow-Back (TLFB). Other outcomes for Aim #1 are 90-day %HDDs, percent days any drinking, alcohol consequences (SIP score), 30-day drinks/drinking days, drinking risky amounts (>7 for women/elderly on average per week [>14 for men] or >3 or >4, respectively, in a day), percentage of participants with no heavy drinking days (PSNHDDs), and the biomarkers serum disialo-carbohydrate deficient transferrin (CDT) and gamma-glutamyl transferase (GGT) (continuous and dichotomized). The primary outcome for Aim #2 is any acute hospital utilization (emergency department visit or inpatient stay) over the past 90 days assessed at 3-month follow-up. Other outcomes for Aim #2 are number of emergency department visits at 30 and 90 days, any emergency department visits (90 days), number of hospitalizations at 30 and 90 days, hospitalizations (90 days), and any emergency department or inpatient stay at 30 days, and all of the noted constructs at 12 months (from the state data).

Medication adherence is not an outcome of the intervention of interest *per se*. But because adherence is likely to be associated with outcome and may be the mechanism by which XR-NTX is more effective than PO-NTX, we will assess whether the participant takes/receives the assigned medication. High adherence to XR-NTX will be defined as administration of XR-NTX by the study nurse 3 times. We will also assess the presence of detectable 6-β-naltrexol to confirm reports of recent tablet taking. We will also assess adherence to study RW-MM visits and exposure to mutual help (e.g., 12-step) groups and specialty alcohol treatment (Form 90). Self-report of outside specialty treatment will also be assessed using statewide utilization databases, but self-report is of primary interest because it captures a wider range of services (e.g., medication, mutual help groups, counseling from non-specialists, and employee assistance programs (EAPs)). Craving has been reduced in prior studies of naltrexone. We will assess this outcome for descriptive purposes but not as a primary analysis.

9.2. Hypotheses

The study will test the main hypothesis that XR-NTX will have greater effectiveness (and cost-effectiveness) than PO-NTX. In addition, factors (e.g., sex, abstinence) may be associated with medication effectiveness.

9.3. Preliminary Analyses

This study will use an intention-to-treat (ITT) analysis including all participants according to their randomized assignment. Descriptive statistics will be calculated for variables at baseline and 3 months. At baseline, all variables will be assessed to see whether there are any differences across treatment arms. Bivariate relationships will be examined using chi-square tests for categorical variables and t-tests or Wilcoxon Rank Sum tests, as appropriate, for continuous variables. Prior to any regression analyses, Spearman correlation coefficients will be obtained to identify variables that may be collinear (r > 0.5); those variables would not be included together in multivariable regression analyses. Using the 2 independent samples t-test and Fisher's exact test, we will compare enrolled participants to those eligible but not enrolled, and those who complete versus those who are lost to follow-up. Missing data patterns and mechanisms will be evaluated. A strength of our design is that administrative utilization data will be available for participants regardless of completion of interviews, data that can be used to assess differences in missingness patterns. We will consider multiple imputation methods and likelihood-based approaches, if needed. With 20% random non-informative loss to follow-up, the study will still have sufficient power.

Main analyses will use multiple regression models that include an indicator variable to represent study arm. To improve efficiency, the regression analyses will control for stratification factors (i.e., sex and race) and factors expected to be highly predictive of outcome (e.g., baseline value of outcome). Models will control for baseline characteristics that differ between groups—potential confounders of particular interest include 7-day abstinence, abstinence goal, readiness to change, number of alcohol use disorder criteria and alcohol consequences, drug use, medical and psychiatric comorbidity, hospital utilization and alcohol treatment. If the data are normally distributed (e.g., SIP scale), multiple linear regression models will be used. However, we anticipate % HDDs will follow a skewed distribution, in which case transformations of the data will be performed (e.g., log). If an appropriate transformation is not identified, a median regression model will be used. Binary outcomes (e.g., any acute healthcare utilization) will be analyzed using logistic regression models, and count data (e.g., number of hospitalizations) will be analyzed using overdispersed Poisson regression models.

10. Staff Training

All study staff will be trained on the study protocol prior to the start of enrollment. The study nurse will be trained in screening procedures, how to administer XR-NTX, as well as conducting RW-MM for participants. Research assistants will be trained in screening, consent, and assessment procedures. Designated prescribers will be trained according to BMC IPS standards.

11. NIAAA

11.1. Funding Statements

The ADOPT trial is funded by the National Institute on Alcohol Abuse and Alcoholism (NIAAA) (Grant Number 1R01AA021335-01A1).

11.2. Clinicaltrials.gov Registration

The ADOPT trial has been registered at clinicaltrials.gov (record number: NCT02478489).

APPENDIX 1.0: STATISTICAL ANALYSIS PLAN

Statistical Analysis Plan

Oral v Injection Naltrexone in Hospital: Comparative Effectiveness for Alcoholism

Alcohol Disorder hospital Treatment trial (ADOPT) Study Protocol

Sponsor: National Institute on Alcohol Abuse and Alcoholism
PI: Richard Saitz, MD, MPH
PI Affiliation: Boston University School of Public Health

This Statistical Analysis Plan (SAP) provides technical specifications and details that correspond tothe approved protocol (version, date and other information specified below). Although the full protocol has several secondary hypotheses, this SAP refers to the primary research hypotheses only. Any changes from the statistical analyses planned from the initial SAP signatory date, will be justified in the subsequent version of the SAP and require additional approval. This SAP is written according to the International Conference on Harmonization Guideline for Industry: Structure and Content of Clinical Study Reports[1] and Statistical Principles for Clinical Trials[2].

Signature Page

Protocol Number: IRB Protocol # H-32911
Study Title: Alcohol Disorder hOsPital Treatment (ADOPT) Study
Trial Registration Number:
Protocol Version Number:
Protocol Date:
SAP Version Date: May 3, 2021
SAP Version Number: 1.0

The undersigned have reviewed this analysis plan and approve of it in its entirety.

Signature	Date	Check if Not Approved
Kim Dukes Digitally signed by Kim Dukes Date: 2021.05.03 12:36:44		☐
Kimberly Dukes, PhD<br>Biostatistician	Date	
Debbie Cheng Digitally signed by Debbie Cheng Date: 2021.05.03 16:22:02 -04'00'		☐
Debbie Cheng, PhD<br>Biostatistician	Date	

Investigators

Richard Saitz Digitally signed by Richard Saitz DN: cn=Richard Saitz, o=Boston University School of Public Health, ou=Department of Community Health Sciences, email=rsaitz@bu.edu, c=US Date: 2021.05.03 14:51:02 -04'00'		☐
Richard Saitz, MD, MPH<br>Principal Investigator	Date	

Revision History

Version		Affected Section(s)	Summary of Revisions
Number	Date		
1.0		n/a	n/a
1.1			

List of Abbreviations:

Abbreviation	Definition
AE	Adverse events
ALT	Alanine Transaminase
AST	Asparate Transaminase
ASSIST	Alcohol, Smoking, and Substance Involvement Screening Test
AUC	Area under the curve
AUD	Alcohol Use Disorder
AUDADIS	The Alcohol Use Disorder and Associated Interview Schedule 5
BMC	Boston Medical Center
CDW	Clinical Data Warehouse
CONSORT	Consolidated Standards for Reporting of Trials
COVID-19	Coronavirus Disease
CRF	Case Report Form
dCDT	Carbohydrate-Deficient Transferrin
DM2	Reasons to Drink
ED	Emergency Department
EMR	Electronic Medical Record
EQ-5D-3L	EuroQol (5 dimensions, 3 levels)
FORM 90 AIR/ED	FORM 90: Alcohol Intake Revised/Economic Development
GAD-7	Generalized Anxiety Disorder
GGT	Gamma-Glutamyl Transferase
HDD	Heavy Drinking Day
ITT	Intent to Treat
MI	Multiple imputation
M-ITT	Modified Intention-to-treat
M2Q	Reasons to Quit
NIAAA	National Institute on Alcohol Abuse and Alcoholism
PACs:	Penn Alcohol Craving Scale
PC-PTSd	Primary Care Post Traumatic Stress Disorder
PEth	Phosphatidylethanol
PHQ8	Patient Health Questionnaire
PO-NTX	Oral Naltrexone
PSS4	Perceived Stress Scale
SAE	Serious Adverse Events
SAP	Statistical Analysis Plan
SES	Socioeconomic Status
SIP2R	Short Inventory of Problems
SOEE	Schedule of Evaluations and Events
TFL	Tables, figures, and listings
TLFB	Timeline Follow-Back
WHO	World Health Organization

WHOQOL-BREF	World Health Organization Quality of Life Assessment (Abbreviated)
XR-NTX	Extended-Release Naltrexone

Table of Contents

1. INTRODUCTION 7
1.1 BACKGROUND AND RATIONALE 7
1.2 OBJECTIVES 7
2. STUDY METHODS 7
2.1 DESIGN 7
2.2 DATA SOURCES 8
3. STUDY PARAMETERS 8
3.1 OUTCOME MEASURES 12
3.2 EXPOSURE MEASURES 12
3.3 CHARACTERISTICS, COVARIATES AND MODIFIERS (CONFOUNDERS AND EFFECT MODIFIERS) 12
3.4 SAFETY PARAMETERS 13
3.6 PARTICIPANT ACCOUNTABILITY AND DISPOSITION 14
3.7 PROTOCOL DEVIATIONS 14
4. STATISTICAL PRINCIPLES 14
4.1 OVERVIEW 14
4.1.1 General *14*
4.1.2. Descriptive *15*
4.1.3. Bivariable *15*
4.1.4. Multivariable *15*
4.2 ANALYSIS POPULATIONS 16
4.3. ANALYSIS OF PARTICIPANT CHARACTERISTICS 16
4.3.1 Participant Accountability. *16*
4.3.2 Baseline Characteristics *16*
4.4 ANALYSIS OF EXPOSURES 16
4.5 ANALYSIS OF OUTCOMES, EXPOSURE, COVARIATES AND MODIFIERS (TABLE 1A AND TABLE 2) 17
4.5.1 Handling of Missing Data *17*
4.6 ANALYSIS OF SAFETY PARAMETERS (TABLE 3) 18
4.7 MODIFICATIONS TO PLANNED ANALYSIS 18
5.0. TABLES, FIGURES AND LISTINGS (TFL) – APPENDIX 2 19
REFERENCES 19
APPENDIX 1: TABLES, LISTINGS AND FIGURES 21

1. Introduction

1.1 Background and Rationale.

As many as 40% of general medical hospital admissions are linked to alcohol, and alcohol use disorder. AUD is a risk factor for readmission, a marker of poor quality of care for which hospitals are financially penalized. Yet, most inpatients with AUD receive no effective treatment, leaving them vulnerable to both alcohol-specific consequences, and the deleterious effects of untreated AUD on medical conditions and related healthcare utilization. Pharmacotherapy for AUD (e.g. oral tablet naltrexone, PO-NTX; extended-release injectable naltrexone, XR-NTX) has efficacy but unlike treatments for other medical conditions routinely started at hospital discharge it is rarely prescribed. In addition, little is known about the effectiveness of pharmacotherapy under real-world conditions compared to other clinically available options, and clinicians have little evidence upon which to base decisions to prescribe a costly (per dose) monthly injection or a daily tablet (possibly lower adherence).

1.2 Objectives.

To test the research hypotheses that XR-NTX will have greater effectiveness than PO-NTX on drinking, consequences, and healthcare utilization. In addition, to test whether some factors may be associated with medication effectiveness.

2. Study Methods

2.1 Design.

This study aims to test the effectiveness of initiating XR-NTX and PO-NTX for AUD in hospitalized inpatients at the time of hospital discharge, anticipated to randomize 260 eligible and consenting participants, in a 3-month pragmatic comparative effectiveness randomized trial. Participants with AUD are recruited from BMC inpatient services and followed through in person visits for a period of three months. Participants with AUD are identified during hospitalization and are screened using a two-stage process (medical records and then interview). Screened eligible and consenting participants complete a baseline assessment and are randomized at the time of hospital discharge to one of two treatment arms: monthly XR-NTX and daily PO-NTX; there is no

control (non-medication) group, all participants will receive naltrexone. Participants are randomized in a 1:1 fashion to treatment group, using permuted block strategy (random block sizes of 2 and 4) stratified by sex and race (African American/Black vs not). Participants, researchers, and nurses performing participant evaluations or real world medical management are not blind to treatment group. Medication is distributed/administered at the baseline, 1- and 2-month visits. All participants will receive monthly real world medical management by a nurse (RW-MM) and all will complete a baseline and 3-month research interview (Figure 1). Adverse events will be collected via self-report at scheduled research contacts and when reported during unscheduled contacts. Utilization information will be obtained through self-report and from the BMC electronic medical record (EMR) through one year post enrollment.

2.2 Data Sources.

Participant information captured via self-report or laboratory results will be recorded either on Case Report Forms (CRF) and then entered into REDCap or will be entered directly into REDCap. EMR data will be sent in electronic format or will be abstracted and entered into REDCap from the BMC clinical data warehouse. Laboratory results will be provided in electronic format from each laboratory vendor or obtained by study staff from the EMR and entered into REDCap. Costs will be derived from utilization data using standard methods.

3. Study Parameters

Figure 1 provides the Schedule of Evaluations and Events (SOEE) categorized by domain for the purpose of the data definition documentation and this SAP. Measurement domains or constructs include participant characteristics; health-related characteristics, utilization and costs; substance use, motives, consequences, craving, readiness and goals; intervention; medication adherence; adverse events and laboratory tests. Data Definition documentation has been developed and organized by each measurement domain provided in Figure 1. The Data Definition documentation provides details regarding the derivation of each measure, reference documentation and evidence of quality control. Study parameters are categorized by study function according to role (e.g., outcome, exposure, covariates, confounders, effect modifiers) in planned analysis described below. These parameters sometimes have more than one role. Note

that not all of the measures or time points for which measures are collected listed in the SOEE will be used in the primary analysis.

Figure 1. Schedule of Evaluations and Events (SOEE)

Construct/Domain	Measures	Visit (Days from Hospital Discharge Date)				
		SCRN	BASE (1-14)	1 Mo (28-55)	2 Mo (56-83)	3 Mo (84-168)
Participant Characteristics						
Eligibility/Consent	Date of Consent, Inclusion/Exclusion Criteria including toxicology (urine) and safety (blood)	X				
Demographics & SES	Age, Sex, Race, Marital/Partner Status, Health Insurance** and Living Situation (homeless)	X	X			X
Health Related Characteristics, Utilization and Costs						
Anthropometrics	Vital Signs, Height, Weight	X	X	X	X	X
Co-Morbidities	Charlson Index (EMR**)		X	X	X	X
Mental Health	Anxiety (GAD-7)<br>PTSD (PC-PTSD)<br>Objective (stressful life events scale/Perceived Stress (PSS_4)<br>Depression (PHQ)<br>AUDADIS	 <br><br><br><br>X	X<br>X<br>X<br><br>X			X
Well-being	Health-related Quality of life (EQ-5D, WHOQOL, Visual Analog Scale)		X			X
Health care Utilization and Concomitant Treatments	Hospitalization, ED Visit, Mutual help groups, alcohol use disorder treatment, substance related nights in hospital, ED visits, outpatient visits, mutual help groups and medication (Form 90 - AIR/ED, BMC CDW**)		X	X	X	X
Injury	Gunshot or stab wound, accidents or falls*		X			X
Pain	Bodily, interfere with daily activities, meds*		X			X
Substance Use, Motives, Consequences, Cravings, Readiness and Goals						
Alcohol Use Disorder Severity	AUDADIS	X				
Alcohol Use	TLFB		X			X
Cigarette and Other Drug Use	EMR, ASSIST		X			X
Craving	PACS		X			X
Consequences	SIP-2R		X			X
Readiness	Readiness to change		X			X
Goals	Abstinence as a Goal		X			
Motives	Reasons to Quit (M2Q)<br>Reasons to drink (DMQ)		X<br>X			X
Intervention						
NTX Route	Randomization Group (XR-NTX – PO-NTX)		X			
Medication Adherence						
Medications	# of NTX injections (nurse, tracking system) or # days taking PO-NTX (participant, Form 90)		X	X	X	X
Visit	Research Visits		X			X
Adverse Events						
Adverse Events	Patient reported side effects, symptoms, alcohol consequences. Also reported during RW-MM visits.	X	X	X	X	X
Laboratory Tests						
Labs	*Biomarkers: 6-β-naltrexol (at 1 month NTX-PO reporting adherence only)@,* dCDT@, PEth@ and plasma/serum (3 Mo only)<br>Safety: Liver Tests (ALT, AST, GGT)*, Pregnancy Test (SCRN as indicated)*	X		X	X	X

*Indicates Adverse Event

@ Indicates assessed for concordance with self-report;
** Indicates information that was collected from enrollment through 12 months past enrollment by the CDW

List of abbreviations: (AE) Adverse Events, (ALT) Alanine Transaminase, (AST) Asparate Transaminase, ASSIST (Alcohol, Smoking, and Substance Involvement Screening Test), AUD (Alcohol Use Disorder), AUDADIS (The Alcohol Use Disorder and Associated Interview Schedule 5), BMC (Boston Medical Center), CDW (Clinical Data Warehouse), dCDT (Carbohydrate-Deficient Transferrin), DM2 (Reasons to Drink), ED (Emergency Department), EMR (Electronic Medical Record), EQ-5D-3L (EuroQol (5 dimensions, 3 levels)), FORM 90 AIR/ED (FORM 90: Alcohol Intake Revised/Economic Development), GAD-7 (Generalized Anxiety Disorder), GGT (Gamma-Glutamyl Transferase), HDD (Heavy Drinking Day) MQ2 (Reasons to Quit), PACS (Penn Alcohol Craving Scale), PC-PTSD (Primary Care Post Traumatic Stress Disorder), PEth (Phosphatidylethanol), PHQ-8 (Patient Health Questionnaire), PO-NTX (Oral Naltrexone), PSS4 (Perceived Stress Scale), SAE (Serious Adverse Events), SES (Socioeconomic Status), SIP2R (Short Inventory of Problems), TLFB (Timeline Follow-Back), WHOQOL-BREF (World Health Organization Quality of Life Assessment (Abbreviated)), XR-NTX (Extended-Release Naltrexone)

3.1 *Outcome Measures*

Primary. Self-reported percent heavy drinking days (HDDs) over the last 30 days will be derived from the validated timeline follow-back (TLFB). The primary outcome is the difference in HDD between baseline and 3 month time points.

Secondary. Secondary outcomes include 1) any acute care hospitalization or emergency department visit determined at 3 months by self-report, 2) alcohol use disorder-related treatment utilization, 3) medication adherence, 4) alcohol consequences, and 5) utilization-related costs.

Utilization measures captured by self-report will be derived using the Form 90 and the EMR include: hospitalization and ED visits, alcohol-related nights in hospital, alcohol related ED visits, alcohol related nights spent at a treatment facility and alcohol related outpatient visits, mutual help (e.g., 12-step) groups and pharmacotherapy for AUD (e.g., disulfiram/antabuse, acamprosate, topiramate and other medicatons). Medication adherence will be classified as low, medium and high, defined using self-reported days taking medication (PO-NTX group) and the number of naltrexone injections administered by the study nurse (XR-NTX group). The metabolite, 6-β naltrexol, assessed at the 1 and 3 month visits will be used to assess medication adherence to naltrexone. Participant report of consequences of drinking will be derived using the validated short inventory of problems (SIP-2R) measure. Utilization-related costs will be derived using national and local sources. Secondary outcomes will be created for baseline (or 1-month where applicable) and the 3- month time points as well as difference scores.

3.2 *Exposure Measures.*

Participants are randomized to monthly injection (XR-NTX - 380 mg) or daily oral (PO-NXT - 50 mg, up to 100 mg if participant continues to report of heavy drinking) naltrexone groups.

3.3 *Characteristics, Covariates and Modifiers (confounders and effect modifiers)*

The following measures will be derived using data captured at baseline. In general, baseline measures will be used for descriptive purposes and for adjustment in the primary analysis (specifications are provided in the Tables, Listings and Figures (TLF- Appendix 2). As noted above, details regarding the derivation of each measure are provided in the Data Definition documentation and organized by each measurement domain provided in Figure 1.

Participant Characteristics. Participant report of baseline demographics includes age, sex, race/ethnicity, education and marital status. Baseline socioeconomic status includes health insurance, living situation (e.g., nights homeless) and incarceration in the past 3 months.

Health Related Characteristics. The Charlson co-morbidity score will be derived from the EMR. Participant report of comorbidities will be obtained through validated instruments and includes: depression (PHQ-8), anxiety (GAD-7), objective (stressful life events scale) and perceived stress (PSS_4), post-traumatic stress disorder (PC-PTSD), injury and pain. Self-reported quality of life measures include EQ-5D and Visual Analog Scale and WHOQOL. Laboratory biomarkers include ALT and AST to measure liver damage obtained at the screening and the 3-month visit (See Laboratory Tests Domain- Figure 1).

Substance Use, Motives, Consequences, Cravings and Readiness. In addition to the primary and secondary outcomes described above, additional measures will be derived and may be included in the primary or subsequent exploratory analysis. Alcohol use disorder severity at baseline will be determined based on DSM 5 criteria (AUDADIS) classified as mild, moderate and severe. Participant report of alcohol use, specficially seven-day abstinence in the past 30 days, % days abstinent from heavy drinking in the past 30 days, % days drinking in the past 30 and number of drinks per drinking day will be derived using the TLFB. Substance use risk scores will be classified using the ASSIST and WHO and NIAAA Drinking Risk Levels, as well as recent substance use with select questions from the ASSIST. Participant report of craving (PACS), readiness to change, abstinence as a goal and motives for and reasons to quit (M2Q) and drink (DMQ) will be derived primarily using standardized, validated instruments. Laboratory biomarkers will be obtained to assess substance use and include PEth (alcohol exposure up to four weeks prior), Gamma-Glutamyl Transferase (GGT) (liver activity sometimes from heavy alcohol exposure) and dCDT (elevated after repeated heavy alcohol exposure) at the 3-month visit (See Laboratory Tests Domain- Figure 1).

3.4 Safety Parameters

Section 7.5 of the ADOPT Protocol describes the definitions of adverse events, serious adverse events and, unanticipated problems and provides a listing of the most common adverse effects (nausea, vomiting, depressive symptoms). Definitions regarding relatedness and severity

are provided. Adverse events are provided at the time of participant visit (or on an additional ad hoc basis as they occur) and recorded in the study Adverse Event log. Adverse events may be reported by the participant, a family member, the medical record, captured as a result of abnormal laboratory values (e.g. ALT, AST) or noted if the participant is reporting opioid use and taking naltrexone.

3.6 Participant Accountability and Disposition

Indicator variables that designate whether the participant was: pre-screened, eligible, consented (See study protocol for definitions), randomized, provided study drug and discontinued as well as whether the participant completed each scheduled visit (baseline, 1-month, 2-month and 3- month) will be created. Reasons for study ineligibility, non-consent, and discontinuation will be summarized. Once enrolled, eligible and consenting participants may choose to discontinue study participation at any time. In addition, participants may be discontinued from the study due to safety concerns (pregnancy, breast feeding, abnormal lab values, concomitant opioid use).

3.7 Protocol Deviations.

Indicator variables that designate modifications to the approved IRB protocol (i.e., ineligible participants enrolled or randomized, treatment received differing from that assigned, content of intervention modified) will be categorized and considered protocol violations/deviations.

4. Statistical Principles

4.1 Overview

4.1.1 General.

This SAP must be approved prior to presentation of results by treatment group to study researchers involved in the analysis. Primary analysis will use the Intent to Treat (ITT) Population (See 4.2). Sensitivity analysis will include the modified ITT Population. Where possible, subgroup analysis will be performed by race and sex randomization strata. Interim analysis, early stopping and adjustment for experiment wise alpha were not planned. Results will be summarized using tables, figures and listings (TFL). Means, medians and model parameter estimates will be

reported to one additional decimal place than the original unit of measure; standard deviations will be reported to two decimal places. In the case of statistical inference, 95% confidence intervals and p-values will be based on a two-sided test where $\alpha=0.05$ (2-sided). Data summaries and statistical analysis will be produced using SAS version 9.4 for Windows.

4.1.2. Descriptive.

Descriptive statistics will be generated for continuous variables and include mean, standard deviation, minimum, 25th percentile, median, 75th percentile, maximum, and number of observations. Descriptive statistics generated for categorical variables include number and percent. The distributional characteristics of variables included in analysis, particularly outcomes will be assessed. For continuous measures, box plots and normal probability (Quantile-Quantile) plots will be generated to determine whether transformations (e.g., Log_e, Log_{10}) will be required to promote normality. For categorical measures, a determination will be made as to whether there are a sufficient number of responses within each response category, and whether subsequent combining of response options will be appropriate or required.

4.1.3. Bivariable.

Associations between treatment group and outcomes (crude model), baseline characteristics, covariates and modifiers will be examined using chi-square or Fisher's Exact tests for categorical variables, and 2 independent sample t-tests, Wilcoxon Rank Sum tests, and Pearson/Spearman correlation coefficients ($r > 0.5$ signifies collinearity), as appropriate, for combinations of categorical and continuous variables. Relationships between measures that will serve as independent variables in multivariable models for primary or exploratory analysis will be assessed for clinical significance as potential confounders or effect modifiers.

4.1.4. Multivariable.

For the primary analysis, statistical adjustment will control for stratification factors (i.e., sex and race) and clinically significant baseline characteristics that appear to differ between groups. Model fit will be assessed using statistics such as r^2 and AUC, as well as plots of actual and predicted outcomes to assess calibration (outcome variable distribution dependent).

4.2 Analysis Populations

Intention-to-treat (ITT): All participants who completed a baseline visit and randomized.

Modified Intention-to-treat (M-ITT): All participants randomized, who received at least one dose of study drug and completed the 3-month research visit.

Safety Population: All participants randomized and received at least one treatment (study drug or counseling)

4.3. Analysis of Participant Characteristics.

4.3.1 Participant Accountability.

Consolidated Standards for Reporting of Trials (CONSORT) Flow Diagram [http://www.consort-statement.org/consort-statement/flow-diagram]. Disposition will be summarized for all participants approached. The number and percent of participants who were screened (2-stage process), eligible, consented, randomized, received study drug, completed 1, 2 and 3 month visits, analysis populations and early withdrawals will be provided (Figure 1). Reasons for ineligibility, refusals, discontinuation and protocol deviations/violations will be classified and summarized and included as an appendix.

4.3.2 Baseline Characteristics.

Participant descriptive characteristics, including randomization strata will be summarized overall and by treatment group; tests for statistical significance will not be provided (Table 1a). To assess whether those screened are significantly different than those enrolled and randomized, baseline characteristics will be assessed using bivariable methods described above.

4.4 Analysis of Exposures

As noted above the exposure measure is an indicator variable as to whether the participant was randomized to either PO-NTX or XR-NTX. The laboratory biomarker 6-β-naltrexol was collected as one measure of adherence to taking medications at the 1- and 3-month visits. Concordance between self-reported adherence and 6-β-naltrexol will be assessed using interclass

correlation coefficients, Kappa and weighted Kappa statistics overall and by treatment group. Adherence is also an outcome measure.

4.5 *Analysis of Outcomes, Exposure, Covariates and Modifiers (Table 1a and Table 2)*

Primary Analyses: Intervention Effects. Multivariable regression methods will be used to test the association between exposure and primary and secondary outcomes. To improve efficiency, the regression analysis will include stratification factors (i.e., sex and race) and clinically important baseline characteristics that appear to differ between groups. If the outcome is normally distributed or has been transformed to promote normality, multiple linear regression will be used. If linear transformations for an outcome measure are not appropriate, non-parametric approaches will be investigated. Binary outcomes will be analyzed using logistic regression, ordinal outcomes will be analyzed using negative binomial or ordinal logistic regression and count data will be analyzed using over dispersed Poisson regression models.

Exploratory Analyses. Confounding will be explored by the comparison of parameter estimates from crude models to those from the multivariable model. Effect modification will be assessed using model interaction terms and graphs to visually depicit these interactions and if clininically meaningful, stratification will be performed. Measures include primary and secondary outcomes (baseline, 3-month and change from baseline to 3-month visits), treatment group by sex, and group by race interactions, other clinically significant interactions and baseline characteristics such as: AUD severity/number of disorder criteria, 7-day abstinence, readiness to change, alcohol consequences, other drug use, medical and psychiatric comorbidity, acute/hospital utilization and alcohol treatment history.

The biomarkers GGT, dCDT and PEth were collected to assess alcohol exposure at 3 months. Concordancebetween self reported drinking (using the TLFB) and GGT, dCDT and PEth will be assessed using interclass correlation coefficients, Kappa and weighted Kappa statistics overall and by treatment group. Exploratory analyses will replace the primary outcome with GGT, PEth, and dCDT.

Sensitivity analysis will use the M-ITT population.

4.5.1 *Handling of Missing Data*

Missing items from Likert-based, standard validated batteries will be imputed as specified in the scoring documentation. If information for imputation is not provided in the scoring documentation, the following approach will be followed: Cronbach's alpha will be generated for each scale and subscale. If the Cronbach's alpha exceeds 0.70, then the mean value of the missing item will be imputed within the scale of sub-scale, provided that no more than 50% of the items within the sub-domain are missing. If more than 50% of the items are missing the subscale or scale will be considered missing. If the scale or measure does include missing data as per instructions and includes summing items, a flag variable will be created for assessment.

Multiple imputation (MI) will be used in the case of missing data at random for baseline characteristics, primary outcome and secondary outcomes to reduce potential bias and increase power (see Appendix 1 for details).

4.6 Analysis of Safety Parameters (Table 3)

The Safety population will be used in the analysis of safety parameters and will include participant report, EMR-detected events, and elevated GGT and liver enzyme tests. The total number of and the number of participants with an AE, SAE and SAE resulting in hospitalization or death will be provided overall and by treatment group. The number of AE, SAE and participants with an AE, SAE attributable to medication will be provided overall and by treatment group. The number of AEs reported with more than 5 per category will be provided overall and by treatment group. Findings will also be examined removing injection site reactions.

4.7 Modifications to Planned Analysis

There was a change to the number of participants randomized from 260 planned to 248 due to the onset of COVID-19. The primary outcome for the originally planned study has not changed: % heavy drinking days. The primary outcome measure for which the power and sample size computations were based is the % Heavy Drinking Days at 3 months (%HDD 3Mo). After enrolling 248 participants but before analysis, we investigated the overall (not by treatment group) distribution of the primary outcome and reviewed whether the assumptions made (e.g., standard deviation of %HDD at the 3-month visit) in the protocol aligned with the data collected. The actual standard deviation of %HDD at the 3-month visit was 35%, our original power

computation assumed 26%. We investigated the distribution of %HDD at baseline and at the 3-month visit, and the distribution of the change in %HDD from baseline to 3 months, and based on this investigation, we decided to use change in %HDD from baseline to 3 months as the primary outcome measure. Power computations were generated using the original assumptions (n=208 M-ITT, stddev=26, estimated 80% power to detect a 12% difference in %HDD) and actual stddev=35, as per the protocol. Different scenarios were run varying the outcome variable measures (i.e., %HDD at 3mo, %HDD Chg Score), sample size (M-ITT: n=208 and n=218 and ITT:n=248 and n=260) using the actual standard deviation. Based on the power analysis the decision was made to discontinue enrollment at 248. With respect to power, under the the most conservative scenario, using the difference score between 3mo %HDD and baseline %HDD, with n=208 evaluable (248 randomized), we will have 80% power to detect a difference of at least - 16.0% or lower in difference scores between treatment groups, using actual stddev=41 with alpha=0.05 and a two-sided test. In the same scenario, with n=260 ITT, we would have 80% power to detect a difference of at least 14.3% or lower in difference scores between treatment groups , using actual stddev=41 with alpha=0.05 and a two-sided test.

5.0. Tables, Figures and Listings (TFL) – Appendix 2

- Figure 1. CONSORT Diagram
- Table 1a. Characteristics of the Study Sample at Baseline
- Table 1b. Baseline and 3-Month Alcohol Use, Other Substance Use and Risk, LaboratoryTests and Health Care Utilization
- Table 2. Association between Treatment and Outcomes
- Table 3. Adverse and Serious Adverse Events

Note: Exploratory and other analysis may be performed to describe the sample and used in the text of the manuscript or provided in appendices

REFERENCES

1. ICH Harmonized Tripartite Guideline E 3. Structure and Content of Clinical Study Reports, November 1995. (www.ich.org; Guidelines; "Efficacy" Topics)

2. ICH Harmonized Tripartite Guideline E 9. Statistical Principles for Clinical Trials, February 1998. (www.ich.org; Guidelines; "Efficacy" Topics)

Appendix 1: Tables, Listings and Figures

Appendix 2: Multiple imputation for missing data

What is multiple imputation? Multiple imputation is a useful strategy to fill in missing values. It generates several different plausible imputed data sets which represent the uncertainty of the missing data, and appropriately combines the results calculated from each of those imputed datasets.

Why we use multiple imputation for missing data? Under the assumption of data are missing at random, we may have an unbiased and statistically more powerful analyses (comparing with analyses based on completed cases) when we include those incomplete data via multiple imputation. We can possibly reduce the effect of withdrawals in the intent-to-treat (ITT) analysis ofrandomized clinical trials.

When we use multiple imputation? Although multiple imputation is a popular approach dealing with missing data, there are still some considerations before its application: 1) the proportions ofmissing data is not negligible (<5%); 2) the proportions of missing data is not substantial (>40%);and 3) data are missing at random. We also need to consider data missing pattern, and auxiliary variables (variables that are correlated with a missing variable or associated with missing) to use for multiple imputation.

How to do multiple imputation (by SAS)? Multiple imputation involves three steps: 1) The missing data are filled in *m* times to generate *m* complete data sets. An imputation method should be specified in this phase. For an arbitrary missing data pattern, a Markov chain Monte Carlo (MCMC)method that assumes multivariate normality can be used. For monotone missing data pattern (e.g., in longitudinal studies with drop-out), either a parametric regression method that assumes multivariate normality or a nonparametric method that uses propensity scores is appropriate. 2) The *m* complete data sets are analyzed by using standard procedures (e.g., linear regression). 3) The results from the *m* complete data sets are combined for the inference. We have two examplesfor conducting multiple imputation by SAS under a monotone missing pattern:

	Continuous outcome (out1)	Binary outcome (out2)
Step 1	proc mi data=analytic out=imputed nimpute=20 seed=7816;<br>class x1 x2 x3;<br>monotone regression(out1 = x1 x2 x3 x4 x5);<br>var x1 x2 x3 x4 x5 out1;<br>run;<br>proc sort data=imputed; by _imputation_; run;	proc mi data=analytic out=imputed nimpute=20 seed=7816;<br>class x2 x3 x6;<br>monotone logistic(out2 = x2 x3 x4 x5 x6);<br>var x2 x3 x4 x5 x6 out2;<br>run;<br>proc sort data=imputed; by _imputation_; run;
Step 2	proc genmod data=imputed;<br>class x1 x2 x3/param=ref;<br>model out1= x1 x2 x3 / dist=normal link=identity type3 covb;<br>by _imputation_;<br>ods output ParameterEstimates=outparms ParmInfo=outparminfo CovB=outcovb;<br>run;	proc genmod data=imputed;<br>class x2 x3 x6/param=ref;<br>model out1= x2 x3 x6/ dist=bin link=logit type3 covb;<br>by _imputation_; ods output ParameterEstimates=outparms ParmInfo=outparminfo CovB=outcovb;<br>run;

Step 3	proc mianalyze parms=outparms parminfo=outparminfo covb=outcovb; modeleffects x1 x2 x3; run;	proc mianalyze parms=outparms parminfo=outparminfo covb=outcovb; modeleffects x2 x3 x6; run;
Programs explanation	A data set with missing values named "analytic", with 20 times imputation by monotone regression (or monotone logistic) method (x1, x2, x3, and x6 are categorical, while x4 and x5 are continuous), we have 20 imputed data sets named "imputed", then in Step 2 we do the linear (or logistic) regression of out1(or out2) on independent variables, and save parameter estimates and covariance. Step 3 generates the combined estimates of effect sizes, confidence intervals, and test statistics.	

We also include a guideline for reporting analysis of missing data by multiple imputation as anappendix (cited from *Sterne Jonathan A C, et al. BMJ 2009; 338: b2393*).

Guidelines for reporting any analysis potentially affected by missing data

- Report the number of missing values for each variable of interest, or the number of cases with complete data for each important component of the analysis. Give reasons for missing values if possible, and indicate how many individuals were excluded because of missing data when reporting the flow of participants through the study. If possible, describe reasons for missing data in terms of other variables (rather than just reporting a universal reason such as treatment failure)
- Clarify whether there are important differences between individuals with complete and incomplete data—for example, by providing a table comparing the distributions of key exposureand outcome variables in these different groups
- Describe the type of analysis used to account for missing data (e.g., multiple imputation), and the assumptions that were made (e.g., missing at random)

For analyses based on multiple imputation

- Provide details of the imputation modelling:
    - Report details of the software used and of key settings for the imputation modelling
    - Report the number of imputed datasets that were created (Although five imputed datasets have been suggested to be sufficient on theoretical grounds, a larger number(at least 20) may be preferable to reduce sampling variability from the imputation process)
    - What variables were included in the imputation procedure?
    - How were non-normally distributed and binary/categorical variables dealt with?
    - If statistical interactions were included in the final analyses, were they also included in imputation models?
- If a large fraction of the data is imputed, compare observed and imputed values
- Where possible, provide results from analyses restricted to complete cases, for comparison with results based on multiple imputation. If there are important differences between the results, suggest explanations, bearing in mind that analyses of complete cases may suffer more chance variation, and that under the missing at random assumption multiple imputation shouldcorrect biases that may arise in complete cases analyses
- Discuss whether the variables included in the imputation model make the missing at random assumption plausible
- It is also desirable to investigate the robustness of key inferences to possible departures from the missing at random assumption, by assuming a range of missing not at random mechanismsin sensitivity analyses.

References:

1. Yang C. Yuan, and Rockville. Multiple Imputation for Missing Data: Concepts and NewDevelopment (Version 9.0). SAS Institute Inc.
2. Jakobsen et al. When and how should multiple imputation be used for handling missing data in randomised clinical trials – a practical guide with flowcharts. *BMC Medical ResearchMethodology* 2017; 17:162.

Sterne Jonathan A C, White Ian R, Carlin John B, et al. Multiple imputation for missing datain epidemiological and clinical research
