# Supplementary material for: Oral vs Extended-Release Injectable Naltrexone for Hospitalized Patients With Alcohol Use Disorder: A Randomized Clinical Trial
Source: JAMA Intern Med. 2025 Apr 21;185(6):635–45. doi: 10.1001/jamainternmed.2025.0522 (PMC12013356; doi:10.1001/jamainternmed.2025.0522)
Supplement: Supplement 2. — eMethods 1. Detailed Methods for the Assessment of Health-Related Characteristics of Participants in the Alcohol Disorder Hospital Treatment (ADOPT) Trial eMethods 2. Details of Real-World Medical Management Provided to Participants in the Alcohol Disorder Hospital Treatment (ADOPT) Trial eMethods 3. Detailed Methods for the Calculation of Health Care Costs During the 3-Month Study Duration for Participants in the Alcohol Disorder Hospital Treatment (ADOPT) Trial eTable 1. Comparison of Self-Reported Acute Health Care Utilization (Hospitalization or Emergency Department Visit) and Data Obtained From Participants’ Electronic Medical Records From the Hospital System at Which the ADOPT Study Occurred eTable 2. Sensitivity Complete Case Analysis of the Primary Outcome and the Secondary Outcome for Oral Naltrexone Compared With Injectable Naltrexone Among Patients With Alcohol Use Disorder in the ADOPT Trial eTable 3. Self-Reported Heavy Drinking Days (TLFB) and Alcohol Biomarkers (PEth, dCDT, and GGT) Among Participants With Alcohol Use Disorder in the Alcohol Disorder Hospital Treatment (ADOPT) Trial eTable 4. Associations Between Treatment Group and Alcohol Biomarkers for Participants With Alcohol Use Disorder in the ADOPT Trial: Exploratory Analyses Replacing the Primary Outcome (Self-Reported %HDDs) With PEth, dCDT, GGT eTable 5. Adverse Events and Serious Adverse Events in the ADOPT Trial eReferences. [file jamainternmed-e250522-s002.pdf]

Supplemental Online Content

Magane KM, Dukes KA, Fielman S, et al. Oral vs extended-release injectable naltrexone for hospitalized patients with alcohol use disorder: a randomized clinical trial. *JAMA Intern Med*. Published online April 21, 2025.
doi:10.1001/jamainternmed.2025.0522

eMethods 1. Detailed Methods for the Assessment of Health-Related Characteristics of Participants in the Alcohol Disorder Hospital Treatment (ADOPT) Trial
eMethods 2. Details of Real-World Medical Management Provided to Participants in the Alcohol Disorder Hospital Treatment (ADOPT) Trial
eMethods 3. Detailed Methods for the Calculation of Health Care Costs During the 3-Month Study Duration for Participants in the Alcohol Disorder Hospital Treatment (ADOPT) Trial
eTable 1. Comparison of Self-Reported Acute Health Care Utilization (Hospitalization or Emergency Department Visit) and Data Obtained From Participants' Electronic Medical Records From the Hospital System at Which the ADOPT Study Occurred
eTable 2. Sensitivity Complete Case Analysis of the Primary Outcome and the Secondary Outcome for Oral Naltrexone Compared With Injectable Naltrexone Among Patients With Alcohol Use Disorder in the ADOPT Trial
eTable 3. Self-Reported Heavy Drinking Days (TLFB) and Alcohol Biomarkers (PEth, dCDT, and GGT) Among Participants With Alcohol Use Disorder in the Alcohol Disorder Hospital Treatment (ADOPT) Trial
eTable 4. Associations Between Treatment Group and Alcohol Biomarkers for Participants With Alcohol Use Disorder in the ADOPT Trial: Exploratory Analyses Replacing the Primary Outcome (Self-Reported %HDDs) With PEth, dCDT, GGT
eTable 5. Adverse Events and Serious Adverse Events in the ADOPT Trial
eReferences.

This supplementary material has been provided by the authors to give readers additional information about their work.

eMethods 1. Detailed Methods for the Assessment of Health-Related Characteristics of Participants in the Alcohol Disorder Hospital Treatment (ADOPT) Trial

Health-related characteristics included self-reported depressive symptoms assessed via the 8-item Patient Health Questionnaire (PHQ-8)[1] (dichotomized as moderate or severe depressive symptoms, score ≥10), anxiety symptoms assessed via the 7-item Generalized Anxiety Disorder (GAD-7)[2] (dichotomized as moderate or severe anxiety symptoms, score ≥10), post-traumatic stress disorder symptoms assessed via the PC-PTSD scale[3], injury or accident requiring medical attention in the past three months, quality of life (general health status, overall quality of life, and specific domain scores for physical, psychological and environmental health) assessed using the WHO Quality of Life Questionnaire (WHOQOL-BREF)[4] and the European Quality of Life Questionnaire 5-Dimension 3-Level (EQ-5D-3L)[5] (summarized using the EQ-5D Index and visual analogue scale (EQ VAS) scores), pain intensity and pain interference over the past 3 months using two questions adapted from the Pain, Enjoyment, General Activity scale[6], Stressful Life Events Scale[7], and the Perceived Stress Scale (PSS-4)[8]. Tobacco use and primary health insurance coverage (Medicaid, Medicare, Private, None or Uninsured) were collected from the electronic medical record at study entry along with diagnoses (utilized to generate Charlson Comorbidity Score[9] with higher scores representing a greater burden of illness, or poorer health status).

© 2025 Magane KM et al. *JAMA Internal Medicine*.

eMethods 2. Details of Real-World Medical Management Provided to Participants in the Alcohol Disorder Hospital Treatment (ADOPT) Trial

Real world medical management (RWMM) was based on concepts of medical management, and was adapted from "Medical Management Treatment Manual: A Clinical Guide for Researchers and Clinicians Providing Pharmacotherapy for Alcohol Dependence (Generic Version; 2010 edition)"[10]. We chose to use RWMM because clinicians to support AUD pharmacotherapy often exist in hospitals and primary care patient-centered medical homes. During RWMM, the study nurse conducted a clinical assessment asking the participant about their alcohol use and related consequences, medication use, medication adherence, side effects, and provided support and recommendations for abstinence or drinking less, for medication adherence and addressing side effects, and for accessing mutual help groups based on patient evaluation. Referrals to more specialized treatment were provided on request. The study nurse collected vital signs, clinically indicated labs, obtained breath alcohol concentration, dispensed/administered study medication (except at the 3-month RWMM visit, wherein no additional study drug was provided due to study conclusion) according to study arm, and assessed for adverse events and alcohol-related medical conditions. During the 3-month RWMM visit, the study nurse offered participants various transition plans, including referral to outpatient addiction treatment or the patient's primary care clinician for continuity prescription and/or other AUD-related care. Standard progress notes were recorded at visits. A randomly selected 5% of RWMM sessions were audio recorded for ongoing supervision by a clinical psychologist and to describe the delivery of RWMM.

© 2025 Magane KM et al. *JAMA Internal Medicine*.

eMethods 3. Detailed Methods for the Calculation of Health Care Costs During the 3-Month Study Duration for Participants in the Alcohol Disorder Hospital Treatment (ADOPT) Trial

Costs were calculated by converting self-reported healthcare utilization episodes (i.e., hospitalizations, ED visits, outpatient visits, and inpatient/residential alcohol treatment), clinical services provided by the study (study nurse time and laboratory test costs), and study medication (PO-NTX or XR-NTX), into cost estimates in US Dollars (USD). Cost of hospitalizations was estimated for each participant by dividing the number of self-reported nights spent in the hospital by the median length of hospital stay for each age group, and multiplying this by the median cost of a hospitalization (by age) as reported in the 2018 hospital inpatient national statistics from the Agency for Healthcare Research and Quality (AHRQ) Healthcare Costs and Utilization Project[11]. Cost estimates for ED visits were calculated by multiplying the number of ED visits reported by a participant by the average total payment for an ED visit in 2018 as reported by the Medical Expenditure Panel Survey from AHRQ[12]. Cost estimates for outpatient visits were calculated by multiplying the number of outpatient visits reported by a participant by the 2019 Medicare fee-for-service non-facility payment per visit (for the locality where this trial took place) for an established patient with moderate complexity[13]. Cost estimates for inpatient/residential alcohol treatment services were calculated using by multiplying the number of days a participant reported spending in a facility by the average cost per day in a treatment facility from the 2016 Department of Agriculture, Trade, and Consumer Protection (DATCAP) data[14]. Cost estimates for PO-NTX were obtained from the 2019 Center for Medicare and Medicaid Services (CMS) Part D Spending by Drug data[15], and were calculated by multiplying the number of pills dispensed per participant during the 3 month study period by the average spending per dosage unit (50mg); XR-NTX cost estimates were obtained from the 2019 CMS Part B Spending by Drug data[16] and calculated by multiplying the number of injections administered during the 3 month study period by the average spending per dose. Cost estimates for clinically indicated laboratory tests, ALT and AST at two timepoints, were calculated by multiplying the cost of the lab test (as listed in the CMS Medicare Fee for Service Clinical Laboratory Fee Schedule[17]) for each completed clinically indicated lab test per participant. Last, cost estimates for registered nurse time was calculated by multiplying hourly wage and fringe benefits for the study nurse by the estimated time the study nurse spent conducting RWMM for each completed RWMM visit per participant.

© 2025 Magane KM et al. *JAMA Internal Medicine*.

eTable 1. Comparison of Self-Reported Acute Health Care Utilization (Hospitalization or Emergency Department Visit) and Data Obtained From Participants' Electronic Medical Records From the Hospital System at Which the ADOPT Study Occurred

	**No Hospitalization or ED Visit Observed in the EMR of the study's hospital system[a] N (%)**	**Hospitalization or ED Visit Observed in EMR of the study's hospital system[a] N (%)**
**Self-Reported Hospitalization or ED Visit[b]**		
No	69 (31.8)	23 (10.6)
Yes	35 (16.1)	90 (41.5)

Abbreviations: ADOPT=Alcohol Disorder hOsPital Treatment study, ED=Emergency Department, EMR=Electronic Medical Record

[a]Participants' EMRs at the hospital system within which the ADOPT study occurred were reviewed for any hospitalizations or ED visits that occurred in the 3-month window between baseline and the 3-month follow-up assessment.

[b]Any self-reported acute healthcare utilization (ED visits or hospitalizations) over the prior 3 months as assessed by research interview at 3-month follow-up.

© 2025 Magane KM et al. *JAMA Internal Medicine*.

eTable 2. Sensitivity Complete Case Analysis of the Primary Outcome and the Secondary Outcome for Oral Naltrexone Compared With Injectable Naltrexone Among Patients With Alcohol Use Disorder in the ADOPT Trial

	**Baseline (n=248)**		**3-Month Follow-Up (n=217)**		**Point Estimate P value**	
	**PO-NTX (n=125)**	**XR-NTX (n=123)**	**PO-NTX (n=109)**	**XR-NTX (n=108)**	**Unadjusted (95% CI)**	**Adjusted[b] (95% CI)**
	**n (%)**	**n (%)**	**n (%)**	**n (%)**		
**Primary Outcome[a]**						
Change in %HDDs at 3 months			-38.4 (43.3)	-46.4 (38.5)	-7.98 (-18.83, 2.87)	-7.70 (-18.47, 3.08)
**Secondary Outcome[a]**						
Hospitalization or ED Visit	75 (60.0)	75 (61.0)	59 (54.1)	66 (61.1)	1.33 (0.78, 2.29)	1.38 (0.80, 2.40)

Abbreviations: ADOPT=Alcohol Disorder hOsPital Treatment study, PO-NTX=Oral Naltrexone, XR-NTX=Injectable Naltrexone, SD=Standard Deviation, %HDDs=percent of heavy drinking days of the prior 30 days, ED=Emergency Department

[a] Models for this complete case analysis use the evaluable population (n=217).
[b] Adjusted for gender and race.

Reference group = PO-NTX

© 2025 Magane KM et al. *JAMA Internal Medicine*.

eTable 3. Self-Reported Heavy Drinking Days (TLFB) and Alcohol Biomarkers (PEth, dCDT, and GGT) Among Participants With Alcohol Use Disorder in the Alcohol Disorder Hospital Treatment (ADOPT) Trial

	**0 HDDs in prior 30 days N (%)**	**≥1 HDDs in prior 30 days N (%)**
**PEth[a]**		
<20 ng/mL	37 (22.8)	2 (1.2)
≥20 ng/mL	35 (21.6)	88 (54.3)
**dCDT[b]**		
Normal (%dCDT<1.7%)	54 (34.4)	29 (18.5)
Abnormal (%dCDT≥1.7%)	15 (9.6)	59 (37.6)
**GGT[c]**		
GGT≤35 U/L	35 (21.5)	20 (12.3)
GGT>35 U/L	37 (22.7)	71 (43.6)

Abbreviations: ADOPT=Alcohol Disorder hOsPital Treatment study, TLFB=Timeline Follow-Back, PEth=phosphatidylethanol, dCDT=Carbohydrate-deficient Transferrin, GGT=Gamma-Glutamyl Transferase, HDDs=Heavy Drinking Days

[a] Denominator is n=162 (80 in PO-NTX arm, 82 in XR-NTX arm) due to 55 participants with missed/incomplete labs.

[b] Denominator is n=157 (77 in PO-NTX arm, 80 in XR-NTX arm) due to 60 participants with missed/incomplete labs.

[c] Denominator is n=163 (79 in PO-NTX arm, 84 in XR-NTX arm) due to 55 participants with missed/incomplete labs.

© 2025 Magane KM et al. *JAMA Internal Medicine*.

eTable 4. Associations Between Treatment Group and Alcohol Biomarkers for Participants With Alcohol Use Disorder in the ADOPT Trial: Exploratory Analyses Replacing the Primary Outcome (Self-Reported %HDDs) With PEth, dCDT, GGT

	**Estimate**		**P value**
	**Unadjusted point estimate (95% CI)**	**Adjusted[a] point estimate (95% CI)**	
**PEth[b]**	0.74 (0.36, 1.52)	0.74 (0.36, 1.54)	0.42
**dCDT[c]**	0.68 (0.36, 1.28)	0.68 (0.36, 1.29)	0.24
**GGT[d]**	0.67 (0.35, 1.29)	0.64 (0.33, 1.26)	0.20

Abbreviations: ADOPT=Alcohol Disorder hOsPital Treatment study, TLFB=Timeline Follow-Back, PEth=phosphatidylethanol, dCDT=Carbohydrate-deficient Transferrin, GGT=Gamma-Glutamyl Transferase, %HDDs=Percentage of Heavy Drinking Days of the past 30, XR-NTX=Extended-release injectable naltrexone

[a] models adjusted for gender and black/African American race

[b] modeling the probability of PEth ≥ 20

[c] modeling the probability of abnormal dCDT (%dCDT≥1.7%)

[d] modeling the probability of GGT >35 U/L

Reference Group = XR-NTX

© 2025 Magane KM et al. *JAMA Internal Medicine*.

eTable 5. Adverse Events and Serious Adverse Events in the ADOPT Trial

		PO-NTX (n=125)		XR-NTX (n=123)	
	**Overall (n=248)**	**Number of Participants Affected**	**Number of Events**	**Number of Participants Affected**	**Number of Events**
Total SAEs	87	44	77	43	66
Hospitalizations	84	42	73	42	63
Deaths	6	4	4	2	2
SAEs related/possibly related to research[a]	6	4	4	2	2
Hospitalizations related to research	5	4	4	1	1
Total AEs (not including serious adverse events)	164	76	204	88	257
**Organ Category[b]**	**AE Term**	**n (%)**		**n (%)**	
Cardiac Disorders<br>AE<br>SAE	Chest Pain	6 (4.8)<br>4 (3.2)	7<br>4	2 (1.6)<br>1 (0.8)	2<br>1
	Hypertension	8 (6.4)<br>0 (0.0)	8<br>0	5 (4.1)<br>1 (0.0)	8<br>2
Gastrointestinal Disorders<br>AE<br>SAE	Abdominal Pain	11 (8.8)<br>4 (3.2)	20<br>8	3 (2.4)<br>1 (0.8)	3<br>1
	Nausea	11 (8.8)<br>0 (0.0)	11<br>0	3 (2.4)<br>0 (0.0)	3<br>0
	Vomiting	8 (6.4)<br>3 (2.4)	9<br>4	5 (4.1)<br>2 (1.6)	7<br>2
	Fatigue	6 (4.8)<br>0 (0.0)	6<br>0	10 (8.1)<br>1 (0.8)	12<br>1
Hepatobiliary Disorders<br>AE<br>SAE	Elevated LFTs	32 (25.6)<br>0 (0.0)	38<br>0	19 (15.4)<br>0 (0.0)	27<br>0
Injury, Poisoning and Procedural Complications<br>AE<br>SAE	Fall	5 (4.0)<br>1 (0.8)	6<br>1	7 (5.7)<br>2 (1.6)	8<br>3
	Intoxication	12 (9.6)<br>0 (0.0)	30<br>0	16 (13.0)<br>3 (2.4)	38<br>3
Nervous System Disorders<br>AE<br>SAE	Dizziness	7 (5.6)<br>0 (0.0)	7<br>0	3 (2.4)<br>1 (0.8)	3<br>1
	Headache	11 (8.8)<br>0 (0.0)	11<br>0	7 (5.7)<br>0 (0.0)	8<br>0
	Seizure	2 (1.6)<br>2 (1.6)	8<br>3	9 (7.3)<br>5 (4.1)	16<br>7
Psychiatric Disorders<br>AE<br>SAE	Alcohol Withdrawal	10 (8.0)<br>10 (8.0)	14<br>12	11 (8.9)<br>6 (4.9)	13<br>7
	Seeking Detox	9 (7.2)<br>0 (0.0)	12<br>0	10 (8.1)<br>1 (0.8)	11<br>1
	Suicidal Ideation	7 (5.6)<br>2 (1.6)	8<br>2	3 (2.4)<br>1 (0.8)	3<br>1
Skin and Subcutaneous Tissue Disorders<br>AE<br>SAE	Injection Site Discomfort	N/A	N/A	36 (29.3)<br>0 (0.0)	52<br>0

Abbreviations: ADOPT=Alcohol Disorder hOsPital Treatment study, PO-NTX=Oral Naltrexone, XR-NTX=Injectable Naltrexone, AE=Adverse Event, SAE=Serious Adverse Events, LFTs=Liver Function Tests, N/A=Not Applicable

[a] SAEs related or possibly related to the research in PO-NTX were suicide attempt, alcohol withdrawal, palpitations, and urinary tract infection and in XR-NTX were suicide attempt and injection site abscess.
[b] Non-serious adverse events by organ category are only included in this table if they occurred at a frequency of 5 or greater in either arm.

© 2025 Magane KM et al. *JAMA Internal Medicine.*

eReferences.

1. Kroenke K, Strine TW, Spitzer RL, Williams JBW, Berry JT, Mokdad AH. The PHQ-8 as a measure of current depression in the general population. *Journal of Affective Disorders*. 2009;114(1-3):163-173. doi:10.1016/j.jad.2008.06.026

2. Löwe B, Decker O, Müller S, et al. Validation and Standardization of the Generalized Anxiety Disorder Screener (GAD-7) in the General Population. *Medical Care*. 2008;46(3):266-274. doi:10.1097/MLR.0b013e318160d093

3. Prins A, Ouimette P, Kimerling R, et al. The primary care PTSD screen (PC–PTSD): development and operating characteristics. *Prim Care Psych*. 2004;9(1):9-14. doi:10.1185/135525703125002360

4. WHOQOL-BREF| The World Health Organization. Accessed October 19, 2022. https://www.who.int/tools/whoqol/whoqol-bref

5. Shaw JW, Johnson JA, Coons SJ. US Valuation of the EQ-5D Health States: Development and Testing of the D1 Valuation Model. *Medical Care*. 2005;43(3):203-220. doi:10.1097/00005650-200503000-00003

6. Krebs EE, Lorenz KA, Bair MJ, et al. Development and Initial Validation of the PEG, a Three-item Scale Assessing Pain Intensity and Interference. *J GEN INTERN MED*. 2009;24(6):733-738. doi:10.1007/s11606-009-0981-1

7. Slopen N, Williams DR, Fitzmaurice GM, Gilman SE. Sex, stressful life events, and adult onset depression and alcohol dependence: Are men and women equally vulnerable? *Social Science & Medicine*. 2011;73(4):615-622. doi:10.1016/j.socscimed.2011.06.022

8. Cohen S, Kamarck T, Mermelstein R. Perceived Stress Scale. Published online February 10, 2014. doi:10.1037/t02889-000

9. Quan H, Sundararajan V, Halfon P, et al. Coding Algorithms for Defining Comorbidities in ICD-9-CM and ICD-10 Administrative Data. *Medical Care*. 2005;43(11):1130-1139. doi:10.1097/01.mlr.0000182534.19832.83

10. Helen M. Pettinati, Roger D. Weiss, William R. Miller, Dennis Donovan, Denise B. Ernst, Bruce J. Rounsaville. Medical Management Treatment Manual: A Clinical Research Guide for Medically Trained Clinicians Providing Pharmacotherapy as Part of the Treatment for Alcohol Dependence. *DHHS Publication No (NIH) 04–5289 Bethesda, MD: NIAAA*. 2004;2. https://pubs.niaaa.nih.gov/publications/combine/

11. HCUPnet Data Tools | AHRQ Data Tools. Accessed January 19, 2023. https://datatools.ahrq.gov/hcupnet

12. Medical Expenditure Panel Survey: Restricted Data Files Available at the Data Centers. Agency for Health Research and Quality. Accessed January 19, 2023. https://www.meps.ahrq.gov/mepsweb/data_stats/onsite_datacenter.jsp

13. Physician Fee Schedule. Centers for Medicare and Medicaid. Accessed January 19, 2023. https://www.cms.gov/medicare/physician-fee-schedule/search/license-agreement?destination=/medicare/physician-fee-schedule/search%3FY%3d2%26T%3d4%26HT%3d0%26CT%3d2%26H1%3d99214%26C%3d107%26M%3d5

14. Murphy SM, McCollister KE, Leff JA, et al. Cost-Effectiveness of Buprenorphine–Naloxone Versus Extended-Release Naltrexone to Prevent Opioid Relapse. *Ann Intern Med*. 2019;170(2):90. doi:10.7326/M18-0227

© 2025 Magane KM et al. *JAMA Internal Medicine*.

15. Medicare Part D Spending by Drug. Centers for Medicare & Medicaid Services Data. Accessed January 19, 2023. https://data.cms.gov/summary-statistics-on-use-and-payments/medicare-medicaid-spending-by-drug/medicare-part-b-spending-by-drug/data?query=%7B%22filters%22%3A%7B%22rootConjunction%22%3A%7B%22label%22%3A%22And%22%2C%22value%22%3A%22AND%22%7D%2C%22list%22%3A%5B%5D%7D%2C%22keywords%22%3A%22vivitrol%22%2C%22offset%22%3A0%2C%22limit%22%3A10%2C%22sort%22%3A%7B%22sortBy%22%3Anull%2C%22sortOrder%22%3Anull%7D%2C%22columns%22%3A%5B%5D%7D

16. Medicare Part B Spending by Drug. Centers for Medicare & Medicaid Services Data. Accessed January 19, 2023. https://portal.cms.gov/MSTR2021/servlet/mstrWeb?evt=2048001&src=mstrWeb.2048001&documentID=203D830811E7EBD800000080EF356F31&visMode=0&currentViewMedia=1&ru=1&share=1&hiddensections=header,path,dockTop,dockLeft,footer&Server=v343069p&Port=0&Project=OIPDA-BI_Prod&

17. Clinical Laboratory Fee Schedule Files. Center for Medicare and Mediaid. Accessed January 19, 2023. https://www.cms.gov/Medicare/Medicare-Fee-for-Service-Payment/ClinicalLabFeeSched/Clinical-Laboratory-Fee-Schedule-Files

© 2025 Magane KM et al. *JAMA Internal Medicine*.
