# Supplementary material for: Oral vs Extended-Release Injectable Naltrexone for Hospitalized Patients With Alcohol Use Disorder: A Randomized Clinical Trial
Source: JAMA Intern Med. 2025 Apr 21;185(6):635–45. doi: 10.1001/jamainternmed.2025.0522 (PMC12013356; doi:10.1001/jamainternmed.2025.0522)
Supplement: Supplement 3. — Data Sharing Statement [file jamainternmed-e250522-s003.pdf]

Data Sharing Statement

Magane. Oral vs Extended-Release Injectable Naltrexone for Hospitalized Patients With Alcohol Use Disorder. *JAMA Intern Med*. Published April 21, 2025.
doi:10.1001/jamainternmed.2025.0522

Data
Additional Information: ClinicalTrials.gov https://clinicaltrials.gov/study/NCT02478489?term=NCT02478489&rank=1 NCT02478489
Data available: Yes
Data types: Deidentified participant data
How to access data: Data will be shared with researchers who request to use the data for proposed meritorious analyses, obtain appropriate regulatory approvals (including IRB approval), execute a signed data sharing agreement and cover costs of data sharing. Requests to access data should be sent to jsamet@bu.edu
When available: With publication

Supporting Documents
Document types: None

Additional Information
Who can access the data: Researchers whose proposed use of the data has been approved.
Types of analyses: For a specified purpose.
Mechanisms of data availability: After approval of a proposal and with a signed data access agreement.
